# Supplementary material for: Stall in Canonical Autophagy-Lysosome Pathways Prompts Nucleophagy-Based Nuclear Breakdown in Neurodegeneration
Source: Curr Biol. 2017 Dec 4;27(23):3626–3642.e6. doi: 10.1016/j.cub.2017.10.054 (PMC5723708; doi:10.1016/j.cub.2017.10.054)
Supplement: Document S1. Figures S1–S7 [file mmc1.pdf]

Current Biology, Volume *27*

Supplemental Information

Stall in Canonical Autophagy-Lysosome Pathways Prompts Nucleophagy-Based Nuclear Breakdown in Neurodegeneration

Olga Baron, Adel Boudi, Catarina Dias, Michael Schilling, Anna Nölle, Gema Vizcay-Barrena, Ivan Rattray, Heinz Jungbluth, Wiep Scheper, Roland A. Fleck, Gillian P. Bates, and Manolis Fanto

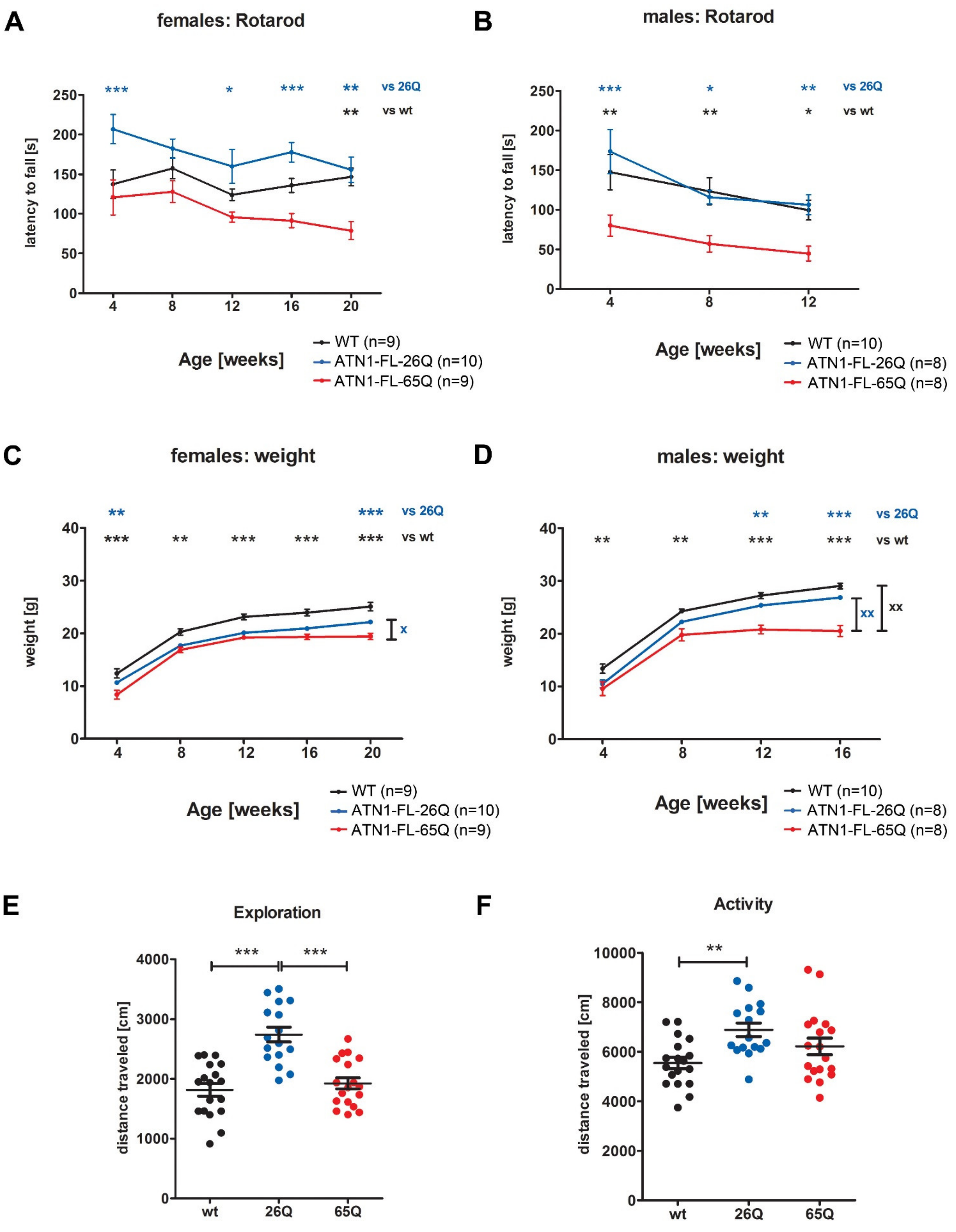

Figure S1. Related to Figure 1 – Behavioral assessment of DRPLA mice

(A – B) Rotarod performance was assed every four weeks starting at four weeks of age. Average values for two experimental days with three trial each were analysed per animal (n) at the given time point. Statistical analysis using repeated measures two-way ANOVA did not show any interaction between genotype (v2) over time (v1) assigning no progressive decrease of rotarod performance. The individual values for females (A) and males (B) at each time point are given as mean ± SEM, showing significantly decreased rotarod performance for the ATN1-FL-65Q line (red) compared to ATN1-FL-26Q (blue) and wild type (WT, black): $^{*}p<0.05$, $^{**}p<0.01$, $^{***}p<0.001$; comparison ATN1-FL-65Q versus wild type (vs wt, black), and ATN1-FL-65Q vs ATN1-FL-26Q (vs 26Q, blue).

(C – D) Weight was assessed every four weeks starting at four weeks of age. Females (C) ATN1-FL-65Q mice (black) showed progressive lack of weight gain only compared to ATN1-FL-26Q (26Q; blue). Male (D) ATN1-FL-65Q mice (black) showed a progressive lack of weight gain compared to ATN1-FL-26Q (26Q; blue) line and wild type mice (red). Repeated measures two-way ANOVA: interaction between genotypes (v2) with age (v1): $^{X}p<0.05$, $^{XX}p<0.01$. The individual values for females (C) and males (D) at each time point are given as mean ± SEM. $^{**}p<0.01$, $^{***}p<0.001$; comparison ATN1-FL-65Q versus wild type (vs wt, black), and ATN1-FL-65Q vs ATN1-FL-26Q (vs 26Q, blue).

(E) Explorative behaviour was assessed in males and females at the age 10 weeks by measuring the activity for the first 5 min after introduction to the open field. ATN1-FL-26Q (26Q; blue) was significantly more active compared to wild type mice (wt; black) and the ATN1-FL-65Q line (65Q; red). The ATN1-FL-65Q mice showed no significant differences compared to their wt littermates. Mean ± SEM, One-way ANOVA, $^{***}p<0.001$.

(F) General activity for females and males showing the total distance travelled over the interval from 5 to 25 min after introduction to the open field. ATN1-FL-26Q (26Q; blue) was significantly more active compared to wild type mice (wt; black) and unchanged compared to ATN1-FL-65Q (65Q, red) at 10 weeks. Mean ± SEM, one way ANOVA, $^{**}p<0.01$.

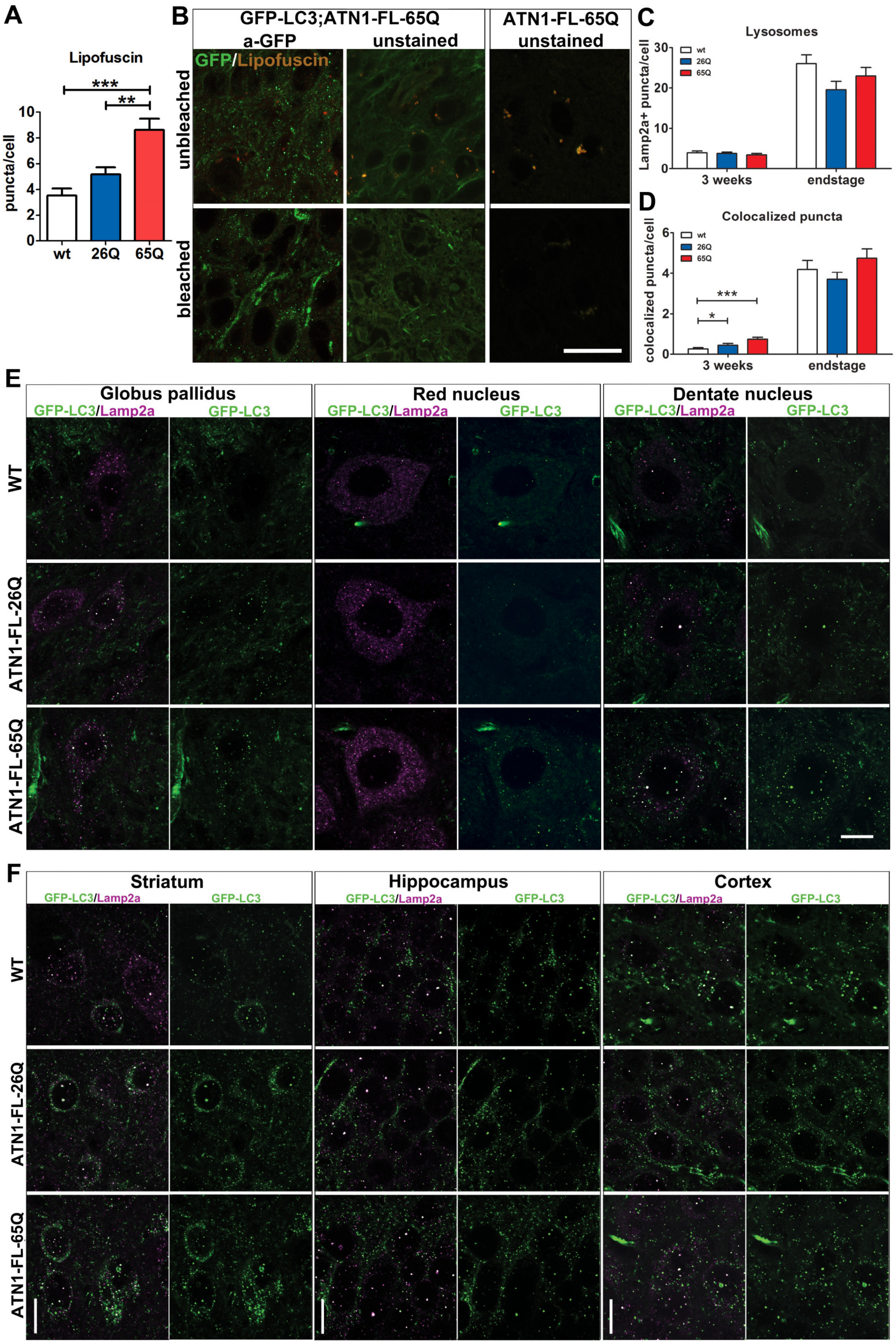
A
Lipofuscin
***
**
puncta/cell
wt
26Q
65Q
B
GFP-LC3;ATN1-FL-65Q
a-GFP
unstained
ATN1-FL-65Q
unstained
GFP/Lipofuscin
unbleached
bleached
C
Lysosomes
Lamp2a+ puncta/cell
wt
26Q
65Q
3 weeks
endstage
D
Colocalized puncta
colocalized puncta/cell
*
***
3 weeks
endstage
E
Globus pallidus
Red nucleus
Dentate nucleus
GFP-LC3/Lamp2a
GFP-LC3
WT
ATN1-FL-26Q
ATN1-FL-65Q
F
Striatum
Hippocampus
Cortex

Figure S2. Related to Figure2 – Anatomical representation of autophagic markers in the brains of DRPLA mice.

(A) Quantification of autofluorescent lipofuscin accumulation in DN neurons in endstage mice. ATN1-FL-65Q mice displayed a significant increase of lipofuscin dots in comparison to ATN1-FL-26Q and WT controls. Mean ± SEM, One-way ANOVA, ***p<0.001, **p<0.01.

(B) Bleaching efficiency of lipofuscin autofluorescence shown on the example of cortical slices form the ATN1-FL-65Q line. Confocal images of lipofuscin-like fluorescence shown in two different channels (red+green=orange) before (top panel) and after (bottom panel) bleaching in non-GFP ATN1-FL-65Q line (right); as well as ATN1-FL-65Q;GFP-LC3 double mutant line in unstained (middle) with native GFP (green) signal and after the staining with mouse α-GFP antibody (left). Scale bar 10 µm.

(C) Statistical analysis of LAMP2a positive puncta in dentate nucleus cells of wt;GFP-LC3 (wt), ATN1-FL-26Q;GFP-LC3 (26Q) and ATN1-FL-65Q;GFP-LC3 (65Q) mice at the presymptomatic stage of 3 weeks (wt, (n=83 cells,3 animals), 26Q (n=77 cells,3 animals), 65Q (n=96 cells,4 animals) and endstage (wt (n=53 cells,3 animals), 26Q (n=49 cells,3 animals), 65Q (n=52 cells, 3 animals). One-way ANOVA, mean ± SEM, not significant.

(D) Statistical analysis of GFP-LC3 positive puncta co-localised with LAMP2a positive puncta in dentate nucleus cells of wt;GFP-LC3 (wt), ATN1-FL-26Q;GFP-LC3 (26Q) and ATN1-FL-65Q;GFP-LC3 (65Q) mice at the presymptomatic stage of 3 weeks (wt (n=83 cells,3 animals), 26Q (n=77 cells,3 animals), 65Q (n=96 cells,4 animals) and endstage (wt (n=53 cells,3 animals), 26Q (n=49 cells,3 animals), 65Q (n=52 cells, 3 animals). One-way ANOVA, mean ± SEM, *p<0.05, ***p<0.001.

(E) Representative images of the three main regions that degenerate in DRPLA patients – globus pallidus (left), red nucleus (middle) and dentate nucleus (right) - from wt;GFP-LC3 (WT, top), ATN1-FL-26Q;GFP-LC3 (middle) and ATN1-FL-65Q;GFP-LC3 (bottom) endstage mice evaluated for GFP-LC3 (green) and LAMP2a (magenta) positive puncta as well as co-localised (white) puncta. Images were taken with the confocal laser scanning microscope. Scale 5 µm.

(F) Representative images of 3 forebrain regions – striatum (left), dentate gyrus of the hippocampus (middle) and cortex (right) - from wt;GFP-LC3 (top), ATN1-FL-26Q;GFP-LC3 (middle) and ATN1-FL-65Q;GFP-LC3 (bottom) endstage mice evaluated for GFP-LC3 (green) and LAMP2A (magenta) positive puncta as well as co-localised (white) puncta. Images were taken with the confocal laser scanning microscope. Scale 5 µm.

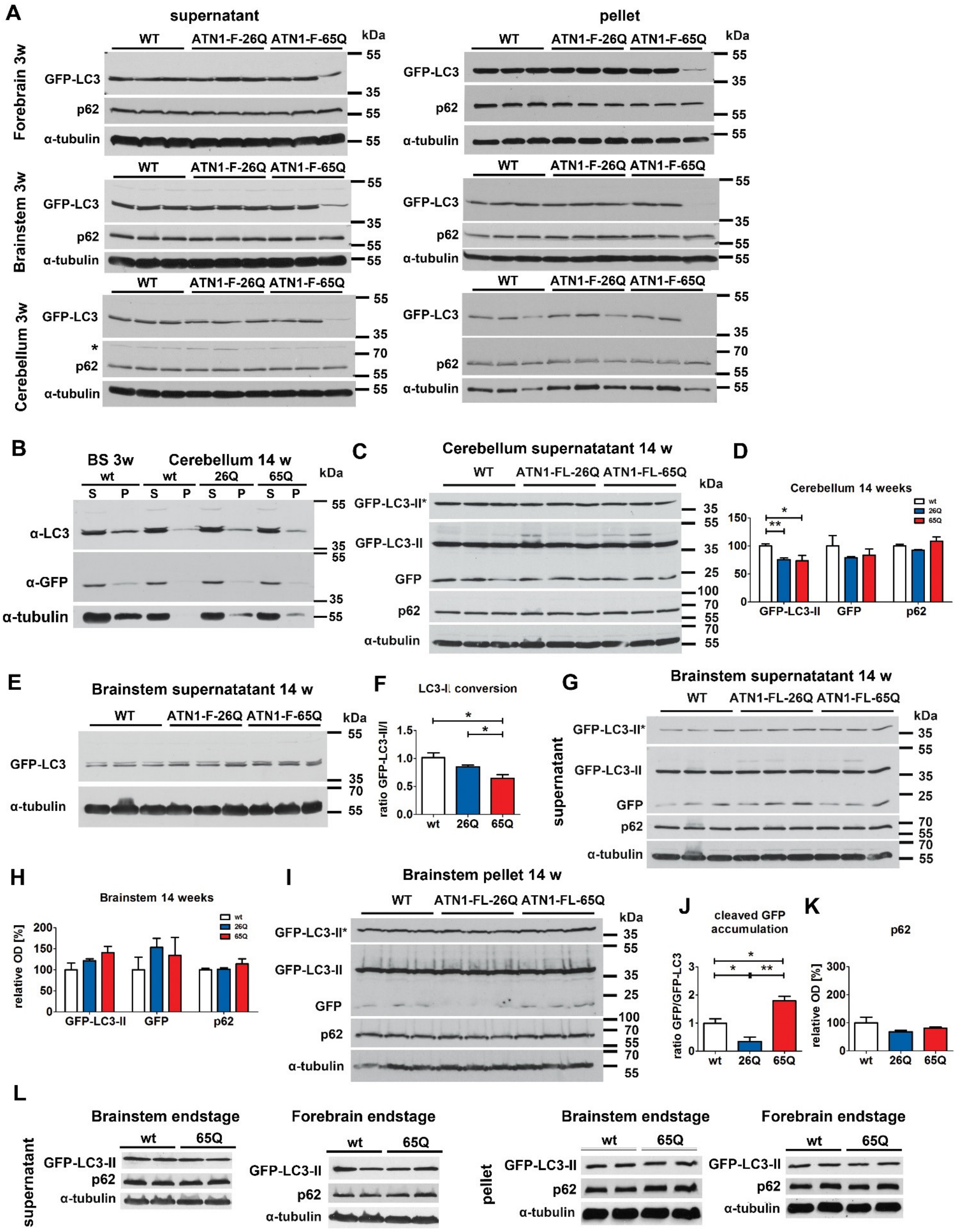

A
supernatant
pellet
WT
ATN1-F-26Q
ATN1-F-65Q
kDa
Forebrain 3w
Brainstem 3w
Cerebellum 3w
GFP-LC3
p62
α-tubulin
*
55
35
70
B
BS 3w
Cerebellum 14 w
wt
26Q
65Q
S
P
α-LC3
α-GFP
α-tubulin
C
Cerebellum supernatatant 14 w
ATN1-FL-26Q
ATN1-FL-65Q
GFP-LC3-II*
GFP-LC3-II
GFP
25
100
D
Cerebellum 14 weeks
150
100
50
0
E
Brainstem supernatatant 14 w
F
LC3-II conversion
ratio GFP-LC3-II/I
1.5
1.0
0.5
0.0
G
Brainstem supernatatant 14 w
H
Brainstem 14 weeks
relative OD [%]
200
I
Brainstem pellet 14 w
J
cleaved GFP accumulation
ratio GFP/GFP-LC3
3
2
1
0
K
p62
300
L
Brainstem endstage
Forebrain endstage

Figure S3. Related to Figure 3 – Autophagy flux analysis in DRPLA mice and patient fibroblasts.

(A) No differences in GFP-LC3 (α-LC3 antibody), and p62 were observed in supernatant (left panel) or pellet (right panel) fractions of forebrain (top), brainstem (middle) and cerebellum (bottom) lysates from three weeks old wt;GFP-LC3, ATN1-FL-26Q;GFP-LC3 and ATN1-FL-65Q;GFP-LC3 mice, except for one outlier sample for ATN1-FL-65Q (last lane), which behaved differently as compared to the eight sister samples. α-tubulin was used as a loading control. * indicates an additional p62 band running at about ~85 kDa besides the expected band at ~62 kDa in the supernatant fraction of the cerebellum only. Molecular weight in kDa (left).

(B) Western blot analysis of GFP-LC3 in supernatant (S) and pellet (P) fractions of the wt;GFP-LC3 brainstem (BS) at three weeks (3w) as well as cerebellum at 14 weeks from wt;GFP-LC3 (wt), ATN1-FL-;GFP-LC3 (26Q) and ATN1-FL-65Q;GFP-LC3 (65Q) mice. α-tubulin was used as a loading control showing a lower abundance in the pellet fractions. The anti-LC3 antibody recognised a GFP-LC3 doublet (GFP-LC3-I corresponds to the upper band, GFP-LC3-II to the lower band) in the supernatant fraction, while only one band appeared in the pellet fraction corresponding to the lower GFP-LC3-II band. The mouse anti GFP antibody recognised only the lower band GFP-LC3-II in both supernatant and pellet fraction.

(C – D) Western blot analysis of GFP-LC3-II, cleaved GFP and p62 in the supernatant fraction of the cerebellar lysates at 14 weeks. Anti-GFP antibody recognises GFP-LC3II and cleaved GFP after longer exposure. *- shorter exposure of anti-GFP-LC3 signal (D). Densitometric analysis of relative abundance of GFP-LC3-II, cleaved GFP and p62 normalised to α-tubulin in supernatant of cerebellum in wt;GFP-LC3 mice (wt), ATN1-FL-26Q;GFP-LC3 (26Q) and ATN1-FL-65Q;GFP-LC3 (65Q) mice. Student's t-test, mean ± SEM, *$p<0.05$, **$p<0.01$ (E).

(E - F) Western blot analysis of full-length GFP-LC3 in the supernatant fraction of the brainstem lysates at 14w of age (E). Densitometric analysis shows decreased relative abundance of cleaved GFP-LC3-II to full-length GFP-LC3-I (F) in ATN1-FL-65Q;GFP-LC3 (65Q) compared to wt;GFP-LC3 (wt) and ATN1-FL-26Q;GFP-LC3 (26Q) mice. Student's t-test, mean ± SEM, *$p<0.05$.

(G – H) Western blot analysis of GFP-LC3II, cleaved GFP and p62 in the supernatant fraction of the brainstem lysates at 14 weeks of age. Anti-GFP antibody recognises GFP-LC3-II and cleaved

GFP after longer exposure. *- shorter exposure of anti-GFP-LC3 signal (G). Densitometric analysis of the relative abundance of GFP-LC3-II, cleaved GFP and p62 relative to α-tubulin in supernatant of brainstem in wt;GFP-LC3 (wt), ATN1-FL-26Q;GFP-LC3 (26Q) and ATN1-FL-65Q;GFP-LC3 (65Q) mice. Student's t-test, mean ± SEM, *p<0.05, **p<0.01 (H).

(I – K) Western blot analysis of GFP-LC3-II, cleaved GFP and p62 in the pellet fraction of the brainstem lysates at 14w (I). Densitometric analysis of relative abundance of cleaved GFP to GFP-LC3-II (J) revealed an increase in ATN1-FL-65Q;GFP-LC3 (65Q) mice compared to wt;GFP-LC3 (wt) and ATN1-FL-26Q;GFP-LC3 The abundance of p62 relative to α-tubulin was unchanged (K). Student's t-test, mean ± SEM, *p<0.05, **p<0.01.

(L) Western blot analysis of GFP-LC3-II and p62 in the supernatant (top) or pellet (bottom) fractions of brainstem (left) and forebrain (right) lysates from endstage wt;GFP-LC3 (wt) and. α-tubulin was used as a loading control.

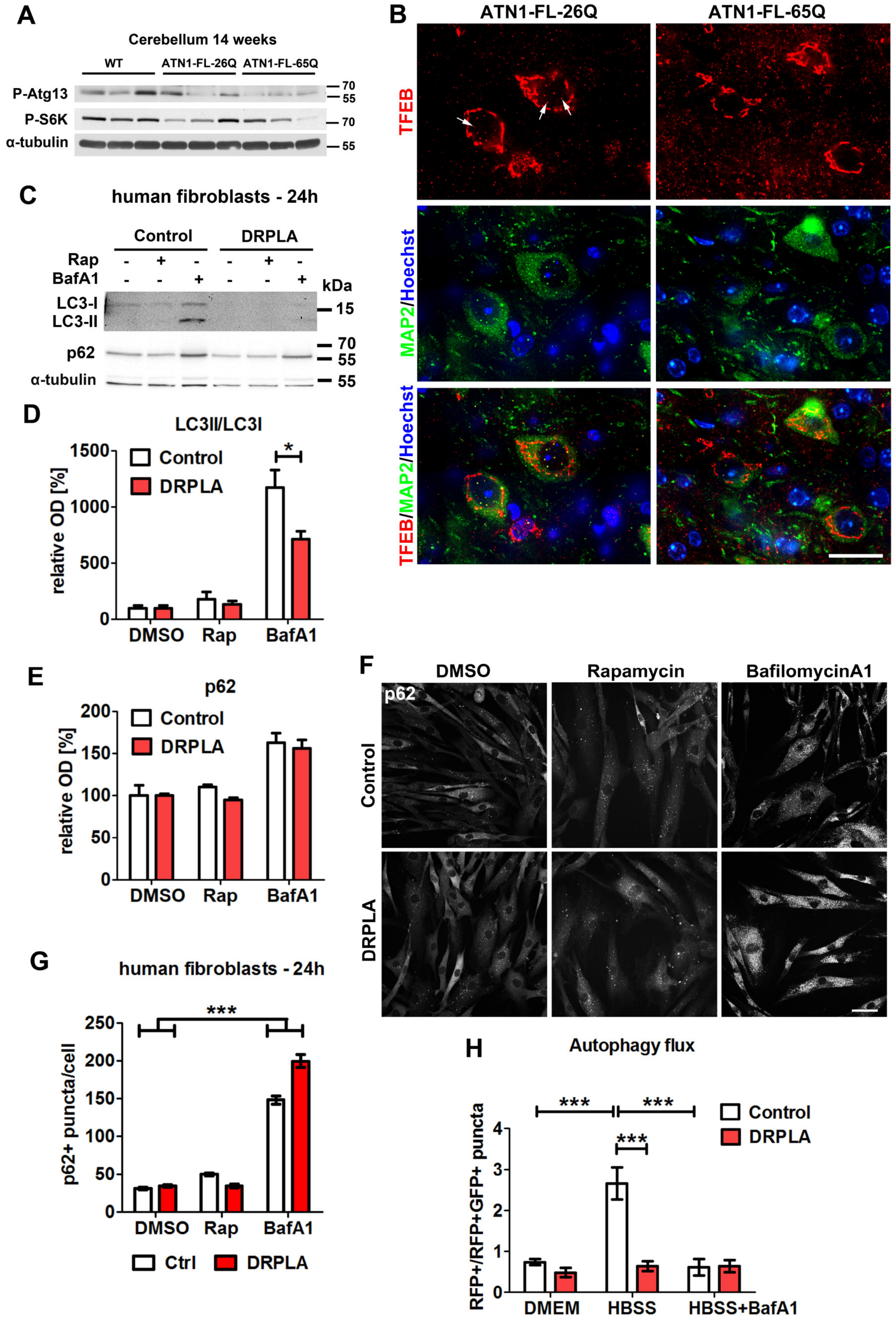
A
Cerebellum 14 weeks
WT
ATN1-FL-26Q
ATN1-FL-65Q
P-Atg13
P-S6K
α-tubulin
70
55
70
55
B
ATN1-FL-26Q
ATN1-FL-65Q
TFEB
MAP2/Hoechst
TFEB/MAP2/Hoechst
C
human fibroblasts - 24h
Control
DRPLA
Rap
BafA1
kDa
LC3-I
LC3-II
p62
α-tubulin
15
70
55
55
D
LC3II/LC3I
relative OD [%]
Control
DRPLA
DMSO
Rap
BafA1
E
p62
relative OD [%]
Control
DRPLA
DMSO
Rap
BafA1
F
DMSO
Rapamycin
BafilomycinA1
p62
Control
DRPLA
G
human fibroblasts - 24h
p62+ puncta/cell
DMSO
Rap
BafA1
Ctrl
DRPLA
H
Autophagy flux
RFP+/RFP+GFP+ puncta
Control
DRPLA
DMEM
HBSS
HBSS+BafA1

Figure S4. Related to Figure 3. – Autophagy flux analysis in DRPLA patients' fibroblasts

(A) Western blot analysis of the phosphorylation status of Atg13 at S318 and p70S6K (phospho-S6K), a target of mTOR kinase in the supernatant fraction of the cerebellar lysates from 14 weeks old wt;GFP-LC3, ATN1-FL-26Q;GFP-LC3 and ATN1-FL-65Q;GFP-LC3 mice. The ATN1-FL-65Q;GFP-LC3 mice show a reduction compared to wt;GFP-LC3. α-tubulin was used as a loading control. Molecular weight in kDa (left).

(B) Representative images of Tfeb staining in DN neurons from endstage mice. MAP2 marks specifically neuronal cells. Tfeb is present in discrete puncta inside the nucleus in ATN1-FL-26Q;GFP-LC3 in contrast to atrophic Map2-positive DN cells in ATN1-FL-65Q;GFP-LC3 mice. Scale bar 20 µm.

(C – E) Supernatants from human fibroblasts from DRPLA (17) patients and age matched healthy control were subjected to western blot analysis for endogenous LC3I and LC3II as well as p62. Block of autophagy with BafA1 for 24h resulted in increase of LC3II as well as p62 compared to DMSO control conditions in control fibroblasts. Densitometry reveals that DRPLA patient samples showed a mild response with a decrease significant decrease in LC3I to II conversion when treated with BafA1 (D). Student's t-test, mean ± SEM, *$p<0.05$, **$p<0.01$.

(F – G) Analysis of p62 puncta in the cytoplasm in DRPLA (17) patient fibroblasts and age matched control after 24 hrs treatment with Rap and BafA1. Representative images show cells after 24 hrs treatment stained with anti-p62 antibody. Scale bar 50 µm. (F). Upon BafA1 treatment both control and DRPLA fibroblasts show an increase in p62 positive puncta, hereby significantly more in DRPLA cells ($p<0.001$). Automated quantification of nuber of p62-positive puncta in the cytoplasm was performed using Opera Phenix high content screening system and Columbus software. Mean ± SEM, two way-ANOVA ***$p<0.001$, v1 – genotype, v2 – treatment.(G).

(H) Ratio of autolysosomes (RFP+) to autophagosomes (GFP+RFP+) as a measure of autophagy flux progression in control and DRPLA fibroblasts transfected with the tandem RFP-GFP-LC3B reporter (shown in Fig 3. M,N). Acute starvation of control cells in HBSS for 2 hours induces a significantly greater transition towards autolysosomes, which is blocked by BafA1 treatment. DRPLA fibroblasts do not display similar formation and maturation of autophagosomes reflected

in significant difference from controls in HBSS. Two-way ANOVA, v1 – genotype, v2 – treatment, mean ± SEM, ***$p<0.001$.

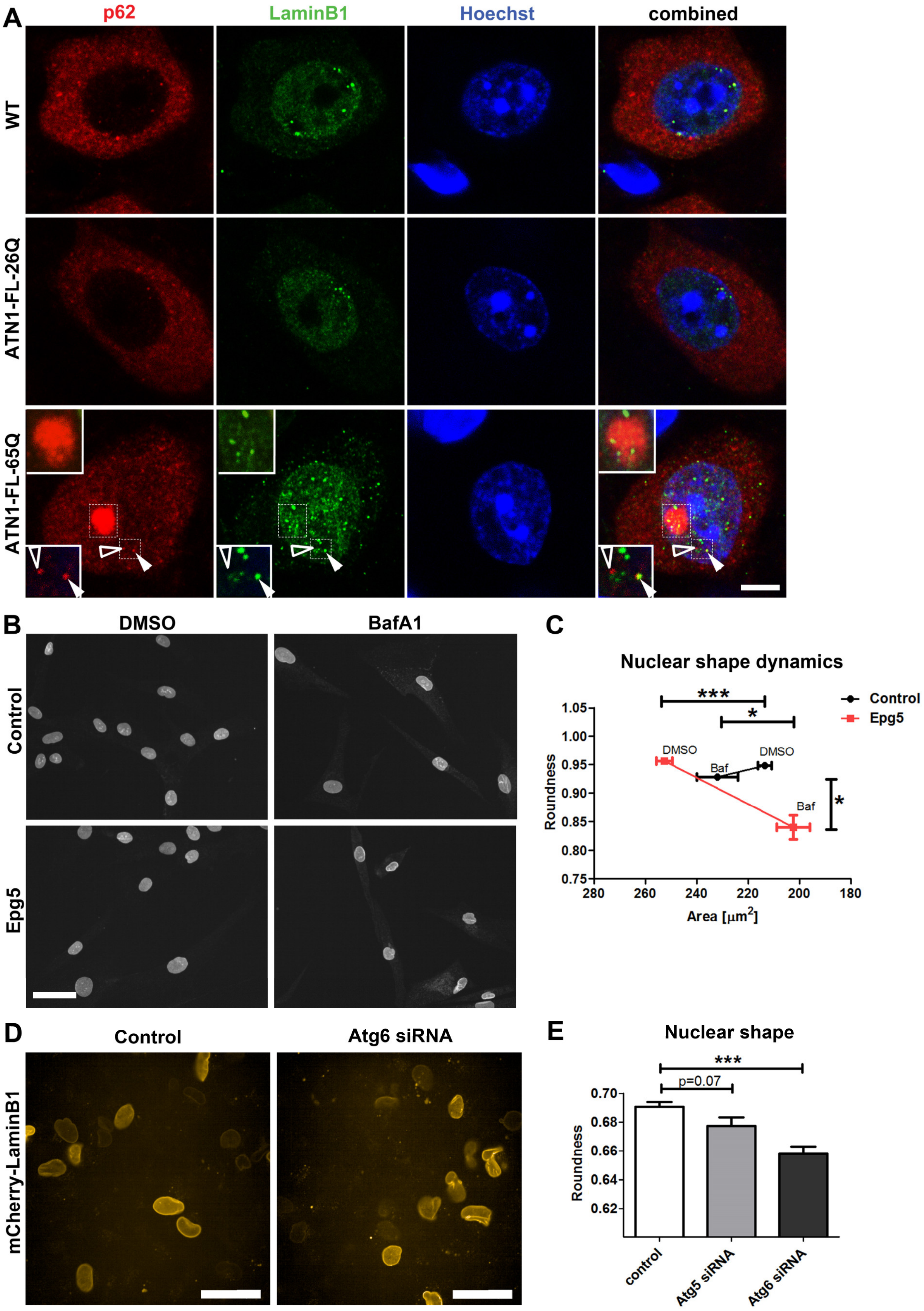

A
p62
LaminB1
Hoechst
combined
WT
ATN1-FL-26Q
ATN1-FL-65Q
B
DMSO
BafA1
Control
Epg5
C
Nuclear shape dynamics
***
*
Control
Epg5
DMSO
DMSO
Baf
Baf
Roundness
Area [μm2]
D
Control
Atg6 siRNA
mCherry-LaminB1
E
Nuclear shape
***
p=0.07
Roundness
control
Atg5 siRNA
Atg6 siRNA

Figure S5. Related to Figure 4 – Nuclear shape disruption upon defective autophagy flux.

(A) Confocal fluorescence microscopy images of the dentate nucleus from endstage wild type (WT), ATN1-FL-26Q and ATN1-FL-65Q mice. Diffuse nuclear Lamin-B1 staining (green) is observed along with speckled staining which is more pronounced in ATN1-FL-65Q mice in the nucleus as well as in the cytoplasm. Lamin-B1 is in close proximity of (framed arrowhead), or co-localises with, p62 (red) puncta (full arrowhead) and the p62 inclusion (inserts). Scale bar 5 µm.

(B – C) Analysis of nuclear shape dynamics in fibroblasts from Vici patient with mutation in autophagy gene EPG5 (p.Phe1604Glyfs*20) and age matched healthy individual. Representative images show nuclei revealed by the LaminB1 antibody upon treatment with BafA1 or DMSO as a control for 48 hrs (B). BafA1 treatment resulted in structural changes in cells carrying mutation in Epg5 gene, reflected by a significant decrease in nuclear size and roundness (D). The plot of roundness versus area shows an opposite trend and convergence of control and Epg5 defective nuclei upon BafA1 treatment (C). Automated quantification was performed using Opera Phenix high content screening system and Columbus software. Mean ± SEM, n=5, two-way-ANOVA ***p<0.001, * p<0.05, v1 – genotype, v2 – treatment. Scale bar 50 µm.

(D – E) Representative images of nuclei in SK-N-BE(2) human neuroblastoma cells expressing mCherry-LaminB1 and Atg6 siRNA or non-specific control siRNA (A). Quantification of nuclear roundness marked by mCherry-LaminB1 reveals a significantly increased irregularity of the nuclear shape in cells overexpressing Atg6 siRNA (B). Student's t-test, mean ± SEM, ***p<0.001. Scale bar 20 µm.

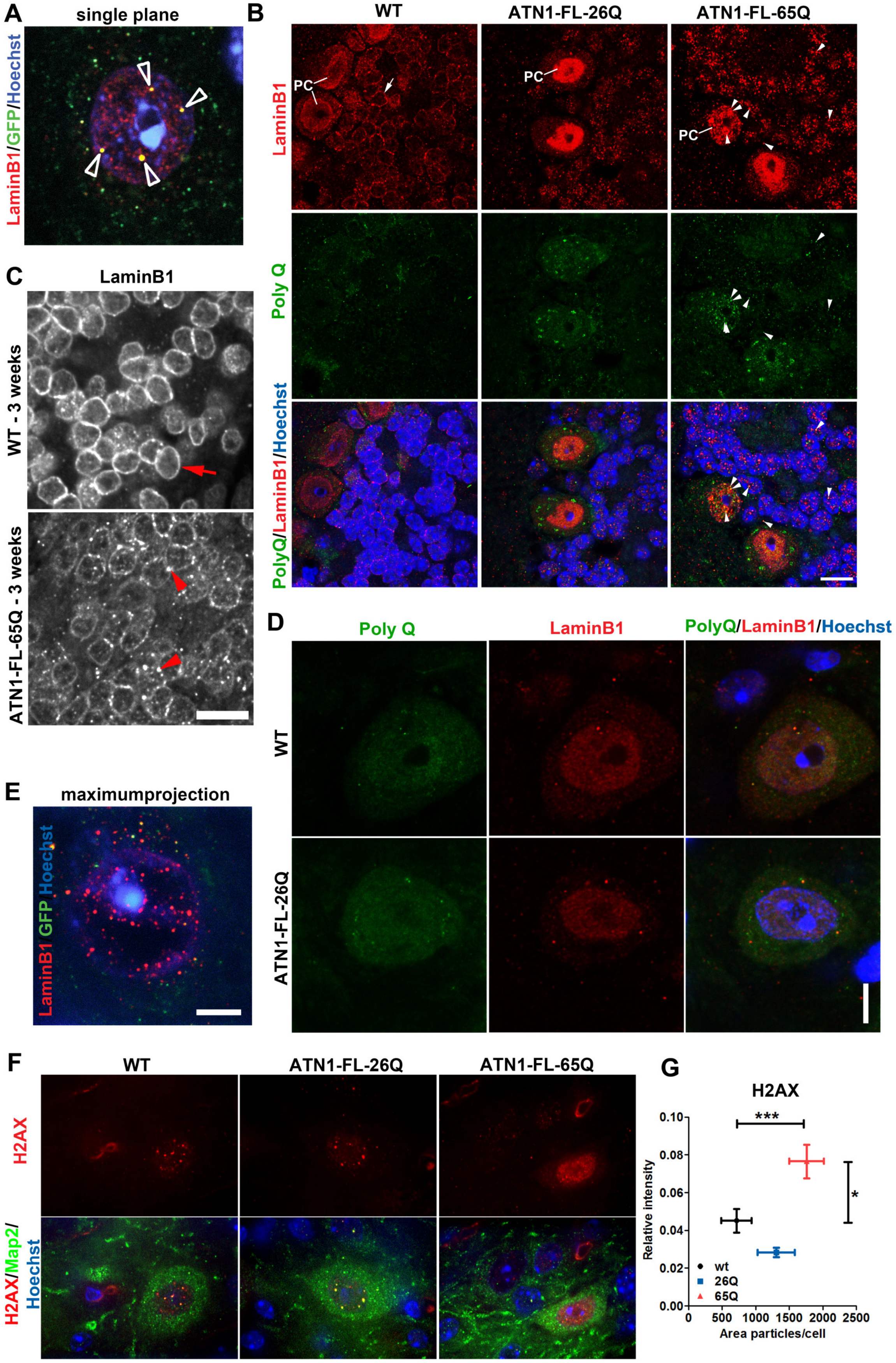

A
single plane
LaminB1/GFP/Hoechst
B
WT
ATN1-FL-26Q
ATN1-FL-65Q
LaminB1
PC
Poly Q
PolyQ/LaminB1/Hoechst
C
LaminB1
WT - 3 weeks
ATN1-FL-65Q - 3 weeks
D
Poly Q
LaminB1
PolyQ/LaminB1/Hoechst
WT
ATN1-FL-26Q
E
maximumprojection
LaminB1 GFP Hoechst
F
WT
ATN1-FL-26Q
ATN1-FL-65Q
H2AX
H2AX/Map2/Hoechst
G
H2AX
***
*
Relative intensity
0.10
0.08
0.06
0.04
0.02
0.00
wt
26Q
65Q
0
500
1000
1500
2000
2500
Area particles/cell

Figure S6. Related to Figure 5 – Nuclear LaminB1 pathology, LaminB1 association with native PolyQ and terminal stage for nuclei in DRPLA mice.

(A) LaminB1 (red) shows a remarkable co-localisation (arrow heads) with GFP-LC3 (green) inside the nucleus (blue) of ATN1-FL-65Q;GFP-LC3 Dentate Nucleus cells. Scale bar 5 μm.

(B) LaminB1 shows a classical perinuclear ring structure (arrow) representing the nuclear lamina cortex in the cerebellar granule cells in wild type (WT) mice, while the Purkinje cells (PC) show, in addition to the lamina ring, a diffuse intranuclear matrix staining. ATN1-FL-26Q mice show reorganization of LaminB1 into speckled matrix in the granule cell nuclei and mostly diffused intranuclear staining in PC. This is more pronounced in the cerebellar cortex of ATN1-FL-65Q mice, in which large LaminB1 speckles form in both cell types colocalise with PolyQ inclusions (arrowheads) in particular in PC. Scale bar 5 μm.

(C) At 3 weeks LaminB1 shows a classical perinuclear ring structure (red arrow) in granule cells of ATN1-FL-65Q mice similarly as in wild type (WT), albeit there is tendency to forming dense LaminB1 punctae in the periphery of the nuclear cortex in ATN1-FL-65Q granule cells (red arrowhead). Scale bar 10 μm.

(D) The α-PolyQ IC2 antibody immunized against polyQ fragment of human TBP also recognises native PolyQ in wild type (WT) and ATN1-FL-26Q mice as distinct focal puncta in the nuclear periphery and cytoplasm. Scale bar 5 μm.

(E) Some of the dentate nucleus cells in ATN1-FL-65Q;GFP-LC3 endstage mice show a severely disrupted nuclear morphology with weak and partially disrupted nuclear staining (blue,), devoid of LaminB1. Maximum projection from confocal z-Stack. Scale bar 5 μm.

(F-G) Analysis of γH2AX levels in DN neurons of endstage mice. Map2 is used to mark neuronal cells. In representative images (F) atrophic cells of ATN1-FL-65Q;GFP-LC3 endstage mice γH2AX is strongly upregulated throughout the nucleus of Map2-positive neurons (green). Scale bar 10 μm. Quantification (G) displays a significant increase in both the area occupied by γH2AX foci and their intensity in ATN1-FL-65Q;GFP-LC3 mice compared to ATN1-FL-26Q;GFP-LC3 and wt;GFP-LC3 controls. The most degenerated DN cells, with hollow nucleus (as in panel B) were not included in the quantification. Kruskal-Wallis multiple comparison analysis, mean ± SEM, ***$p<0.001$, *$p<0.05$.

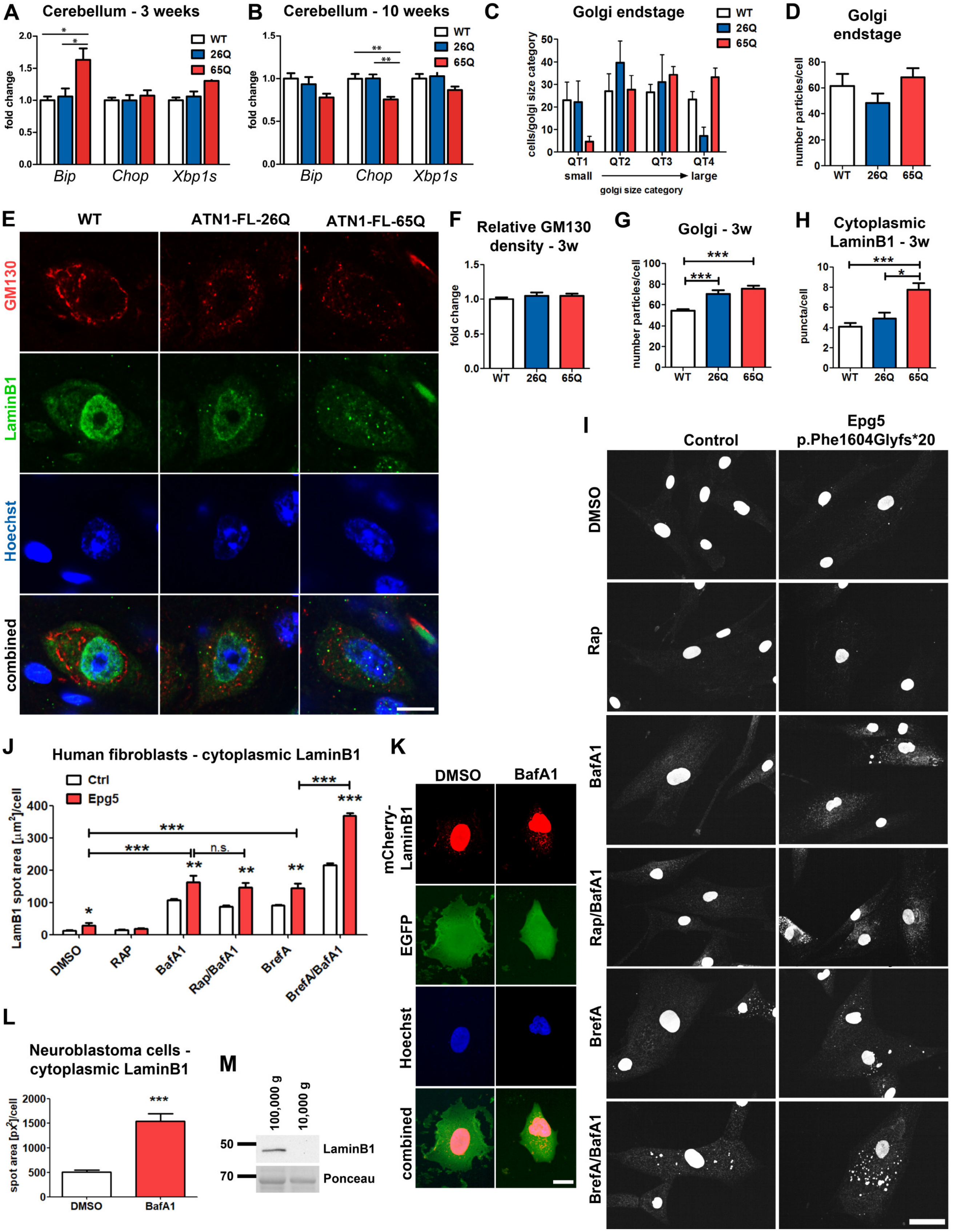
A
Cerebellum - 3 weeks
WT
26Q
65Q
fold change
Bip
Chop
Xbp1s
B
Cerebellum - 10 weeks
C
Golgi endstage
cells/golgi size category
QT1
QT2
QT3
QT4
small
large
golgi size category
D
Golgi endstage
number particles/cell
E
WT
ATN1-FL-26Q
ATN1-FL-65Q
GM130
LaminB1
Hoechst
combined
F
Relative GM130 density - 3w
G
Golgi - 3w
H
Cytoplasmic LaminB1 - 3w
puncta/cell
I
Control
Epg5 p.Phe1604Glyfs*20
DMSO
Rap
BafA1
Rap/BafA1
BrefA
BrefA/BafA1
J
Human fibroblasts - cytoplasmic LaminB1
Ctrl
Epg5
LamB1 spot area [µm²]/cell
n.s.
DMSO
RAP
BafA1
Rap/BafA1
BrefA
BrefA/BafA1
K
DMSO
BafA1
mCherry-LaminB1
EGFP
Hoechst
combined
L
Neuroblastoma cells - cytoplasmic LaminB1
spot area [px²]/cell
M
100,000 g
10,000 g
LaminB1
Ponceau

Figure S7. Related to Figure 6 and 7. – Transient ER stress activation correlates with differentially progressing structural Golgi alterations in DRPLA mice. Golgi dependent LaminB1 degradation.

(A – B) ER stress activation was assessed in the cerebellum of 3 weeks (A) and 10 weeks (B) old ATN1-FL-65Q mouse (65Q, red), ATN1-FL-26Q (26Q, blue) and wild type (wt; white) mice. The levels of the UPR target mRNAs Bip, Chop and Xbp1s were determined by qPCR. *Hprt1* was used as reference gene. Values were normalised to wt and statistically analysed by one-way ANOVA (*$p<0.05$ and **$p<0.01$). Graph represents mean values ± SEM (n = 6 animals).

(C – D) Quantification of Golgi size in endstage mice according to average size of GM130-positive structures in the cells distributed in 4 categories QT1 to QT4 (small to large) defined according to values at 25‰ intervals in WT mice). ATN1-FL-65Q mice show more cells in the DN which have on average larger GM130-positive structures, while ATN1-FL-26Q show a shift towards on average smaller GM130-positive structures (C). On average, the number of GM130 positive structures was unchanged. Kruskal-Wallis multiple comparison anlalysis, Mean ± SEM, not significant.

(E-H) Representative spinning disc confocal images of GM130-positive Golgi structures and LaminB1 immunoreactivity in the DN cells of 3 weeks old animals. Scale bar 10 µm (E). Quantification of GM130-positive structures showed no significant changes with respect to the total area occupied by GM130-positive staining in the cells between genotypes (F). The significantly increased number of GM130-positive particles (G) correlates well with decreased average size of GM130-positive structures (data not shown) in ATN1-FL-26Q and ATN1-FL-65Q mice, indicating Golgi fragmentation at an early presymptomatic stage of 3 weeks. DN cells ATN1-FL-65Q mice show an increased number of LaminB1 positive puncta, some colocalising with GM130, in the cytoplasm already at presymptomatic stage of 3 weeks compared to wild type and ATN1-FL-26Q mice. Kruskal-Wallis multiple comparison analysis, Mean ± SEM, *$p<0.05$, **$p<0.01$ and ***$p<0.001$.

(I-J) Analysis of LaminB1 redistribution into the cytoplasm in fibroblasts from healthy controls and Vici Syndrom patients carrying mutations in the autophagy gene Epg5. Cells were treated for 48 hours with DSMO as a control or Rap, BafA1, BrefA, as well as combination of BafA1/Rap, and BafA1/BrefA. BrefA was only added for the last 24 hrs. BafA1 and BrefA treatment stimulate localisation of LaminB1 into the cytoplasm. Scale bar 50 µm (I). Automated quantification of area occupied by LaminB1 positive punctae

in the cytoplasm was performed using Opera Phenix system and Columbus softwarec (J). Fibroblasts with Epg5 mutation showed increased cytoplasmic LaminB1 levels in control condition and were more sensitive to all treatments with respects to control. Significance levels above the column, mean ± SEM, two-way-ANOVA ***p<0.001, **p<0.01, v1 – genotype, v2 - treatment. Treatment with BafA1/BrefA combination resulted in synergistic effect showing a significant increase compared to BafA1 and BrefA. In contrast BafA/Rap did not increase cytoplasmic LaminB1 levels in comparison to the BafA treatment alone: significance levels shown above the horizontal bars were determined by one-way-ANOVA ***p<0.001.

(K – L) Representative images of SK-N-BE(2) human neuroblastoma cells expressing EGFP and mCherry-LaminB1 and treated with BafA1 or DMSO as a control for 48 hrs (K). Automated quantification of area occupied by mCherry positive punctae in the cytoplasm was performed using Opera Phenix system and Columbus software BafA1 induced significant cytoplasmic accumulation of ectopically expressed mCherry-LaminB1 in the cytoplasm of EGFP positive cells (L). Note also the misshapen nucleus in the BafA1 treated cell compared to the control. Student’s t-test, mean ± SEM, ***p<0.001. Scale bar 5 µm.

(M) Medium supernatant from DRPLA fibroblasts fractionated by ultracentrifugation. Truncated LaminB1 is specifically found in the fraction collected after ultracentrifucgation at 100,000g, known to contain extracellular vesicles. No LaminB1 is found in the fraction collected upon 10,000g ultracentrifugation, known to contain apoptotic bodies. A ponceau ban is used as a control for loading. Its molecular weight is consistent with that of BSA, a major component of the medium supernatant.
