# Supplementary material for: Stall in Canonical Autophagy-Lysosome Pathways Prompts Nucleophagy-Based Nuclear Breakdown in Neurodegeneration
Source: Curr Biol. 2017 Dec 4;27(23):3626–3642.e6. doi: 10.1016/j.cub.2017.10.054 (PMC5723708; doi:10.1016/j.cub.2017.10.054)
Supplement: Document S2. Article plus Supplemental Information [file mmc6.pdf]

Article

Current Biology

Stall in Canonical Autophagy-Lysosome Pathways Prompts Nucleophagy-Based Nuclear Breakdown in Neurodegeneration

Highlights

- Progressive stall in canonical autophagy in disease-specific areas in DRPLA
- Chronic autophagy block activates alternative degradation pathways *in vivo*
- Nucleophagy-associated LaminB1 accumulates in the cytoplasm and is then excreted

Authors

Olga Baron, Adel Boudi, Catarina Dias, ..., Roland A. Fleck, Gillian P. Bates, Manolis Fanto

Correspondence

manolis.fanto@kcl.ac.uk

In Brief

Golgi-mediated degradation and excretion of LaminB1 promote cell atrophy and death. Baron et al. demonstrate that a block in canonical autophagy signaling leads to activation of alternative clearance routes. Golgi-mediated degradation and excretion of nuclear LaminB1 finally result in terminal nuclear breakdown, cell atrophy, and death.

Baron et al., 2017, Current Biology *27*, 3626–3642
December 4, 2017 © 2017 The Authors. Published by Elsevier Ltd.
https://doi.org/10.1016/j.cub.2017.10.054

Current Biology
Article

Stall in Canonical Autophagy-Lysosome Pathways Prompts Nucleophagy-Based Nuclear Breakdown in Neurodegeneration

Olga Baron,[1] Adel Boudi,[1] Catarina Dias,[1] Michael Schilling,[1] Anna Nölle,[2,3] Gema Vizcay-Barrena,[4] Ivan Rattray,[5] Heinz Jungbluth,[1,6,7] Wiep Scheper,[2,3] Roland A. Fleck,[4] Gillian P. Bates,[5,8] and Manolis Fanto[1,9,*]
[1]Department of Basic and Clinical Neuroscience, King's College London, 125 Coldharbour Lane, SE5 9NU London, UK
[2]Department of Clinical Genetics and Alzheimer Center, VU University Medical Center, Amsterdam, the Netherlands
[3]Department of Functional Genome Analysis, VU University, Amsterdam, the Netherlands
[4]Centre for Ultrastructural Imaging, King's College London, SE1 1UL London, UK
[5]Department Medical and Molecular Genetics, School of Basic and Biomedical Sciences, King's College London, SE1 9RT London, UK
[6]Department of Paediatric Neurology, Neuromuscular Service, Evelina's Children Hospital, Guy's & St. Thomas' Hospital NHS Foundation Trust, London, UK
[7]Randall Division for Cell and Molecular Biophysics, Muscle Signaling Section, King's College London, London, UK
[8]Sobell Department of Motor Neuroscience, UCL Institute of Neurology, WC1N 3BG London, UK
[9]Lead Contact
*Correspondence: manolis.fanto@kcl.ac.uk
https://doi.org/10.1016/j.cub.2017.10.054

SUMMARY

The terminal stages of neuronal degeneration and death in neurodegenerative diseases remain elusive. Autophagy is an essential catabolic process frequently failing in neurodegeneration. Selective autophagy routes have recently emerged, including nucleophagy, defined as degradation of nuclear components by autophagy. Here, we show that, in a mouse model for the polyglutamine disease dentatorubral-pallidoluysian atrophy (DRPLA), progressive acquirement of an ataxic phenotype is linked to severe cerebellar cellular pathology, characterized by nuclear degeneration through nucleophagy-based LaminB1 degradation and excretion. We find that canonical autophagy is stalled in DRPLA mice and in human fibroblasts from patients of DRPLA. This is evidenced by accumulation of p62 and downregulation of LC3-I/II conversion as well as reduced Tfeb expression. Chronic autophagy blockage in several conditions, including DRPLA and Vici syndrome, an early-onset autolysosomal pathology, leads to the activation of alternative clearance pathways including Golgi membrane-associated and nucleophagy-based LaminB1 degradation and excretion. The combination of these alternative pathways and canonical autophagy blockade, results in dramatic nuclear pathology with disruption of the nuclear organization, bringing about terminal cell atrophy and degeneration. Thus, our findings identify a novel progressive mechanism for the terminal phases of neuronal cell degeneration and death in human neurodegenerative diseases and provide a link between autophagy block, activation of alternative pathways for degradation, and excretion of cellular components.

INTRODUCTION

Nuclear homeostasis has recently been the focus of attention in aggregation-prone neurodegenerative disorders. In particular, defects in nucleo-cytoplasmic transport and deregulation of nuclear matrix have been identified as potential pathomechanisms in several conditions [1–4]. Mutation in genes encoding nuclear lamina constituents have been associated with degradation of nuclear components by autophagy [5], in a process further defined as nucleophagy.

Autophagy affects onset and progression of several human neurodegenerative diseases, reflecting its key role as a regulator of neuronal proteostasis and organelle quality control [6]. Bulk or selected cargos are recruited by autophagy receptors, like p62, to forming double-membrane vesicles, the autophagosomes, marked by the lipid-conjugated form of LC3, LC3-II. Mature autophagosomes fuse with lysosomes to form autolysosomes where the cargo is digested by lysosomal enzymes and basic molecules are recycled back to the cytoplasm [7]. Nucleophagy, a selective autophagy mechanism, has been linked to LaminB1 degradation through direct interaction with LC3 as a mechanism of protection from oncogenesis and of reinforcement toward cellular senescence [8]. However, the importance of nucleophagy and its relationship with neuronal degeneration has not been established.

Polyglutamine (polyQ) diseases are neurological conditions due to an expanded CAG repeat resulting in polyQ stretches in the encoded protein. This family of disorders includes Huntington's disease, dentatorubral-pallidoluysian atrophy (DRPLA), and several spinocerebellar ataxias. DRPLA is caused by the expansion of a CAG stretch in the

 © 2017 The Authors. Published by Elsevier Ltd.
This is an open access article under the CC BY license (http://creativecommons.org/licenses/by/4.0/).

CellPress

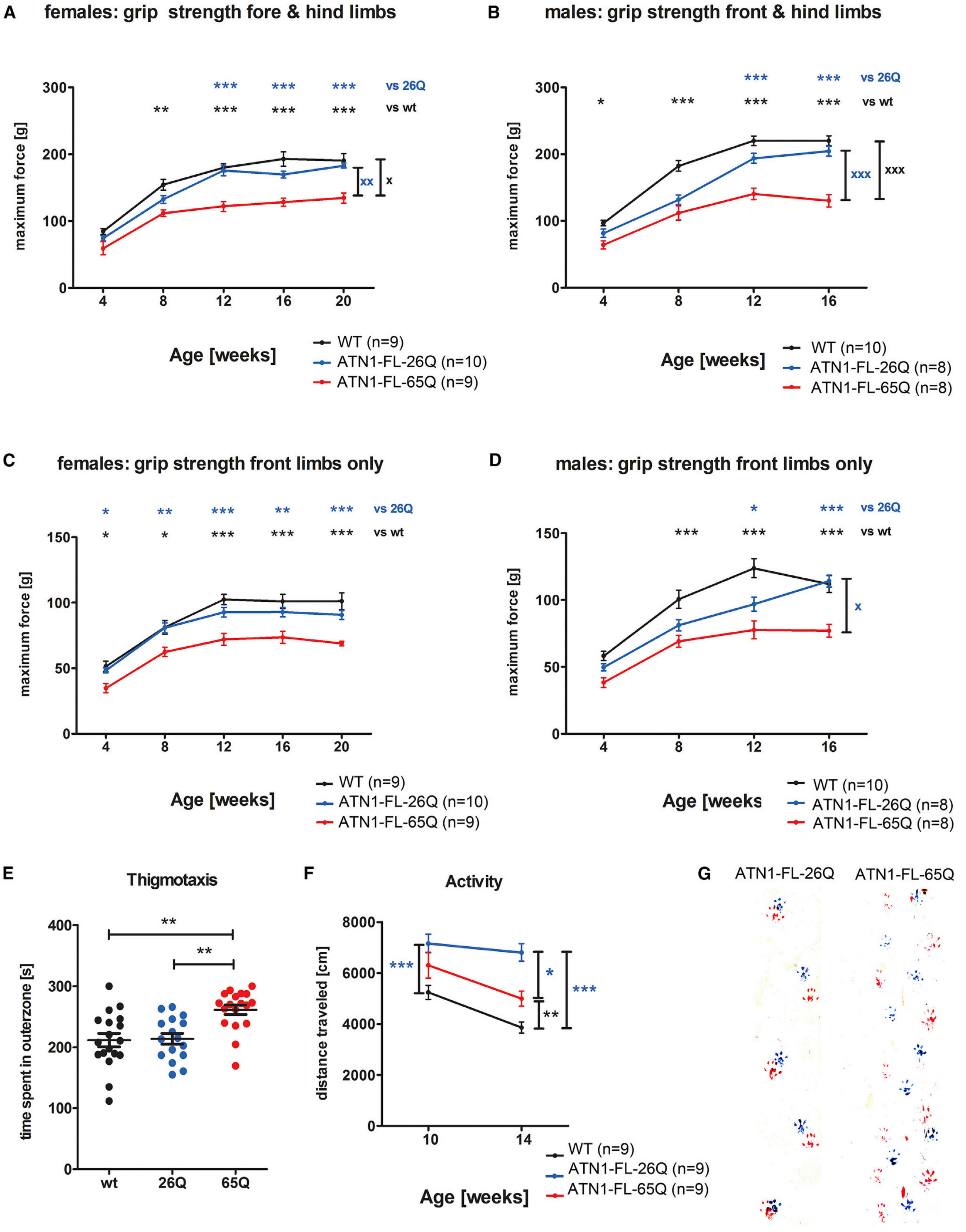

(legend on next page)

ATROPHIN-1 (*ATN1*) gene [9]. Patients display ataxic and choreoathetoid symptoms as well as myoclonus, generalized epilepsy, and dementia with extensive cellular degeneration found in the basal ganglia (e.g., the globus pallidus, GP), brainstem (e.g., the red nucleus, RN), and cerebellum (primarily in the dentate nucleus, DN) [10].

Several DRPLA mouse models have been previously generated, all recapitulating important aspects of the disease [11–13]. We have predicted dysfunctional autophagy from previous *Drosophila* studies on DRPLA [14, 15]. Here, we show that progressive development of an ataxic phenotype in DRPLA mice is linked to severe cellular pathology in relevant neuroanatomical regions. We reveal that neurodegeneration is associated with a stall in canonical autophagy and the activation of alternative pathways of Golgi-dependent and nucleophagy-based degradation and excretion of LaminB1, leading to disruption of nuclear integrity and to cell atrophy.

RESULTS

Progression of Motor Behavior Defects in DRPLA Mice

The behavioral phenotypes of ATN1-FL-26Q-84 (ATN1-FL-26Q) and ATN1-FL-65Q-105 (ATN1-FL-65Q) mouse lines were evaluated in greater detail than previously reported. Compared to both wild-type (WT) mice and the ATN1-FL-26Q-84 (ATN1-FL-26Q) line, the ATN1-FL-65Q-105 (ATN1-FL-65Q) line showed clear decline in the rotarod (Figures S1A and S1B) and grip strength tests (Figures 1A–1D). This was also reflected in the earlier onset of jerky movements, tremors, hind limb clasping, seizures, and a stronger progressive lack of weight gain (Figures S1C and S1D; Movie S1).

Furthermore, the ATN1-FL-26Q and ATN1-FL-65Q lines seemed to be hyperactive compared to WT. ATN1-FL-26Q mice showed increased explorative behavior (Figure S1E; Movie S2), while the ATN1-FL-65Q mice appeared rather aggressive and anxious, as reflected in increased thigmotaxis (Figure 1E) [16]. The ATN1-FL-26Q line also showed a significant increase in general activity as compared to WT mice (Figures 1F and S1F). ATN1-FL-65Q, but not ATN1-FL-26Q, displayed a severely altered, distinctively ataxic gait (Figure 1G).

Stall in Autophagy in DRPLA

Previous *Drosophila* studies indicated a blockage of autophagic clearance in DRPLA [14].

Lipofuscin accumulates during aging and also in lysosomal storage disorders with dysfunctional autophagy [17]. We found a significant accumulation of lipofuscin in DRPLA mouse models in several brain regions (Figures 2A–2C, S2A, and S2B).

This was confirmed in transmission electron microscopy (TEM) micrographs from DN cells (Figures 2D–2F) showing electron-dense tertiary lysosomes, characterized as lipofuscin, especially in the ATN1-FL-65Q mice (Figures 2F and 2G). Furthermore, in ATN1-FL-65Q we observed accumulations of multilamellar bodies (Figure 2I), and of double-membrane autophagic vesicles, often containing partially preserved debris (Figures 2J–2L), suggesting incomplete digestion.

The autophagy flux was evaluated *in vivo* using GFP-LC3 transgenic mice [18]. DN cells in ATN1-FL-65Q mice showed a significant increase in GFP-LC3 puncta, as early as 3 weeks of age, with further increase observed in end-stage animals (Figures 2M and 2N). Similar effects were observed in the RN in the brainstem (Figure S2E), but not in the GP (Figure S2F) and in any other forebrain region (Figure S2E). The ATN1-FL-26Q line showed an initial increase of GFP-LC3 puncta in the DN at 3 weeks but not at end-stage (Figures 2M and 2N) in any of the brain areas analyzed (Figures S2E and S2F).

The number of LAMP2A positive lysosomes did not differ across genotypes (Figure S2C); however, the number of GFP-LC3/LAMP2A double-positive autolysosomes was increased in ATN1-FL-65Q (and partially in ATN1-FL-26Q) DN cells at 3 weeks (Figure S2D). At end-stage, normalizing the GFP-LC3/LAMP2A double-positive puncta, over the total of GFP-LC3 puncta (reflecting the ratio of autolysosomes over the total of autophagic vesicles) the number of autolysosomes had actually decreased in the ATN1-FL-65Q and ATN1-FL-26Q lines (Figure 2O). This contrasts with the initial increase at 3 weeks of age in the ATN1-FL-65Q, suggesting a change in the status of autophagy with pathology progression.

We also biochemically analyzed autophagy on a global scale. The extraction process separated a liquid supernatant from a pellet fraction. No changes were detected at 3 weeks of age for GFP-LC3 and p62 in any brain area (Figure S3A). Thus, the mild autophagic flux alterations observed at this stage are limited

Figure 1. Behavioral Assessment of DRPLA Mice

(A–D) Grip strength analysis revealed the progression of degenerative decline in ATN1-FL-65Q mice (red) compared to wild-type mice (WT, black) and ATN1-FL-26Q (blue) over time as measured by repeated-measures two-way ANOVA. This was evidenced by significant interaction between age (v1) and genotype (v2) ($^{X}p < 0.05$, $^{XX}p < 0.01$, $^{XXX}p < 0.001$) when measuring both limbs (A and B). Hereby the progression was stronger in males signified by stronger interaction in both limbs (B) compared to females (A). In addition, males showed progression when only forelimb grip strength was measured (D). In contrast, females showed overall decreased non-progressive grip strength levels for fore limbs (C). Individual values are given as mean ± SEM and significance levels for individual time points are assigned above with $^{*}p < 0.05$, $^{**}p < 0.01$, and $^{***}p < 0.001$.

(E) Thigmotaxis as a measure of anxiety was evaluated for the first 5 min after introduction to the open field by assessing the time 10-week-old males and females spent in the outer zone. The ATN1-FL-65Q (65Q, red) line showed a significantly higher tendency to remain close to the walls of the arena as compared to the wild-type (wt; black) and ATN1-FL-26Q (26Q; blue) mice. Automatic quantification using EthoVision 7XT software. One-way ANOVA, $^{**}p < 0.01$.

(F) General activity was assessed in females at 10 and 14 weeks evaluating the distance traveled from 5 to 25 min after introduction to the open field. ATN1-FL-26Q (blue) mice were significantly more active compared to the wild-type (WT; black) at 10 weeks. This difference was more pronounced at 14 weeks of age with both the ATN1-FL-26Q and ATN1-FL-65Q (red) lines being more active than the wild-type, while ATN1-FL-65Q mice were less active than ATN1-FL-26Q. An interaction with age was not observed (repeated-measures two-way ANOVA, v1, genotype; v2, age). Automatic quantification using EthoVision 7XT software. One-way ANOVA $^{*}p < 0.05$, $^{**}p < 0.01$, and $^{***}p < 0.001$.

(G) In gait analysis at 18 weeks of age, ATN1-FL-26Q shows a typical coordinated and regular footprint placing the hind paws (red) close to front paws (blue). In contrast, ATN1-FL-65Q shows an uncoordinated pattern typical for ataxia.

See also Figure S1.

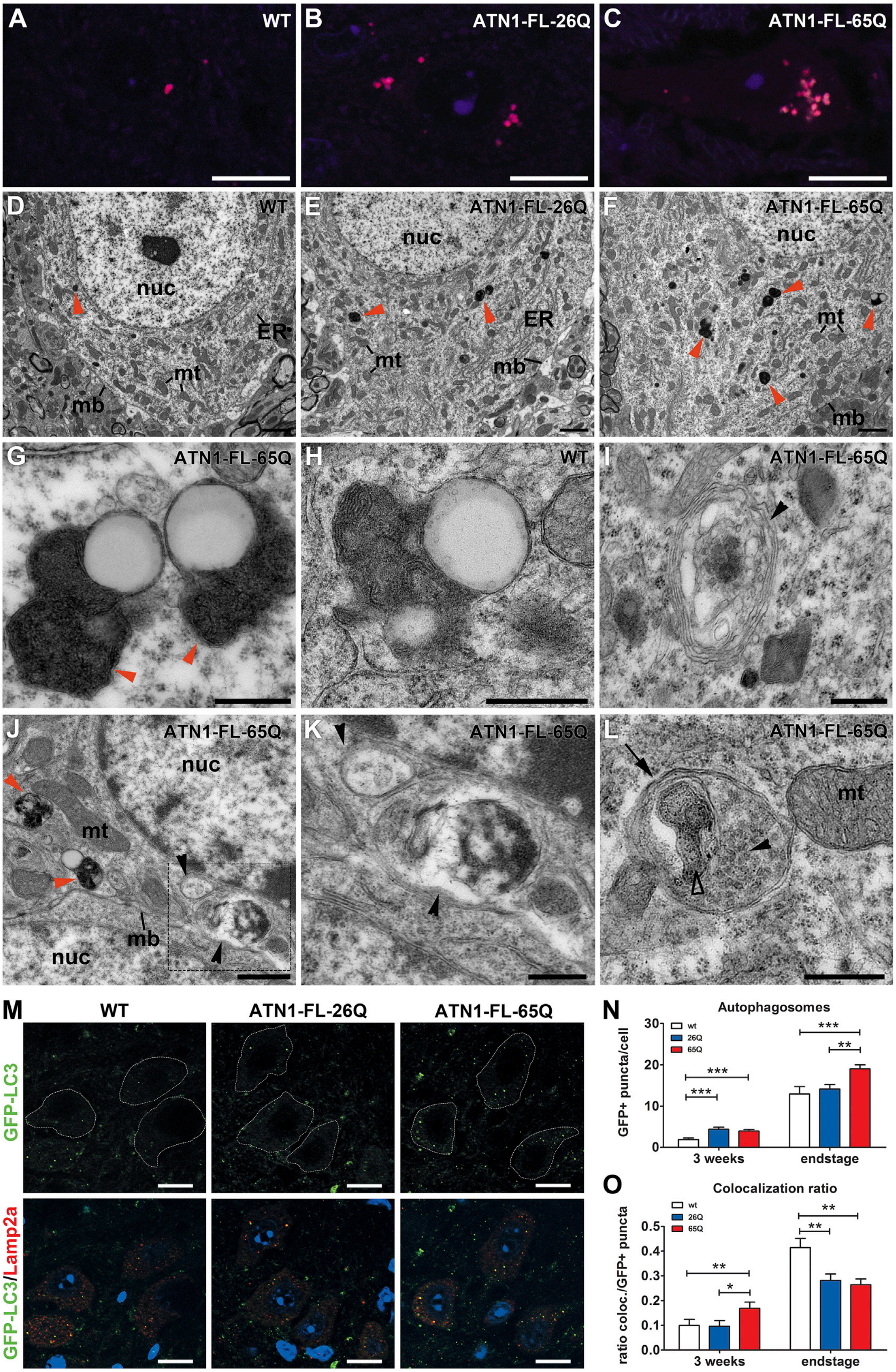

(legend on next page)

to the DN of DRPLA mice. However, pronounced global defects become apparent at the symptomatic stage. In the 14 weeks cerebellum, there was a significant downregulation of the Atg5-12 conjugate formation and of the GFP-LC3-II/-I ratio (Figures 3A–3C), as well as of GFP-LC3-II (Figures S3C and S3D) in ATN1-FL-65Q in the supernatant fraction, which was accompanied by a significant increase in the cleaved GFP/GFP-LC3 ratio and of the levels of p62 in the pellet (Figures 3D–3F). These changes were also maintained at end-stage (Figure 3G).

A similar, albeit somewhat delayed, effect to that in the cerebellum was visible in the brainstem lysates of the ATN1-FL-65Q line (Figures S3E–S3K). The ATN1-FL-26Q line appeared comparable to WT, except for a decrease in the cleaved GFP/GFP-LC3 ratio at 14 weeks of age (Figures S3I and S3J). Finally, no autophagy alteration was detected in the forebrain at any time point (Figure S3L).

Despite robust similarities indicating block at lysosomal level as observed in *Drosophila* DRPLA models, in DRPLA mice we detected additional events that indicate the presence of feedback inhibition on autophagy signaling.

The alterations in the cerebellum at the symptomatic stage of 14 weeks are consistent with a block in autophagic clearance, combined with a reduction in signaling required for the formation of new autophagosomes, perhaps result of a feedback loop. In agreement with this, we detected a reduction in phosphorylation of Atg13 on S318 (Figure S4A) and a significant decrease in mRNA and protein levels of Tfeb (Figures 3H–3J), the master regulator of the autophagosome-lysosome system [19]. The feedback loop did not appear to be mediated by activation of mTOR, which displayed rather a trend toward inactivation, as shown by phosphorylation of p70S6 kinase (Figure S4A). The repression of Tfeb in ATN1-FL-65Q mice is also indicated by absence from the nucleus of DN neurons (Figure S4B), and decreased transcription of at least two of its target genes (Figure 3K). The ATN1-FL-26Q line showed almost no difference from WT with the exception of a mild reduction in the GFP-LC3-II/-I ratio (Figures 3A and 3B) and GFP-LC3-II (Figures S3I and S3J) at 14 weeks in the cerebellum, as well as a tendency to reduction in mTOR signaling (Figure S4A).

To validate whether the stall in autophagy signaling in DRPLA is present also in human cells, we used fibroblast lines from two DRPLA patients. The human fibroblasts failed to respond to acute 6-hr treatment with the autophagy inducer Rapamycin (Rap) or with the autophagy clearance blocker Bafilomycin A1 (BafA1), whereas a control fibroblast line displayed the typical increase in LC3I to LC3II conversion, upon Rap treatment, as well as increased accumulation of LC3II, upon treatment with BafA1 (Figure 3L). Chronic 24-hr treatment with BafA1 elicited a modest but reproducible response from the DRPLA human fibroblasts, including significant reduction in LC3I/II conversion and accumulation of p62 (Figures S4C–S4G), suggesting a block in autophagosomal biogenesis. We further confirmed the block in autophagosome formation in DRPLA cells with the tandem reporter RFP-GFP-LC3B (Figures 3M and 3N). The ratio between RFP^+ and RFP^+/GFP^+ positive puncta in DRPLA cells did not increase with starvation and was also not affected by additional treatment with BafA1, suggesting also impaired progression and clearance of the few autophagosomes present (Figure S4H). Thus, patients' cells display the same phenotype detected at late stages in the progression of cellular pathology in DRPLA mice, i.e., canonical autophagy signaling, the formation of new autophagosomes, and their clearance are stalled.

Figure 2. Accumulation of Undigested Autophagic Structures in Cells of the Dentate Nucleus at End-Stage in DRPLA Mice

(A–C) Increased accumulation of lipofuscin-like autofluorescence in the dentate nucleus of ATN1-FL-65Q (C) and a lower level of increase in ATN1-FL-26Q (B) mice compared to wild-type (A). Confocal laser scanning microscope image λex = 514 and 633. Scale bar, 10 μm. For quantification, see Figure S1G; for examples of autofluorescence bleaching, see Figure S2A.

(D–F) Low-magnification TEM images of DN cells show an accumulation of tertiary lysosomes as electron dense vesicular structures with transparent lipid inclusions, known as lipofuscin (red arrowheads) in ATN1-FL-65Q mice (F) compared to wild-type (WT, D) and ATN1-FL-26Q (E). nuc, nucleus; mt, mitochondria; ER, endoplasmatic reticulum; mb, plasma membrane. Scale bar, 1 μm. Higher-magnification image of (F) is shown in Figure 5B.

(G and H) High-magnification TEM images of tertiary (late) lysosomes resemble the typical structure of lipofuscin-packed electron dense lamellar matrix containing intravesicular lipid inclusion as an electron transparent circular structure. (G) ATN1-FL-65Q and (H) wild-type (WT). mt, mitochondria. Scale bar, 500 nm.

(I) High-magnification TEM image shows dense accumulation surrounded by multiple membranes (black arrowhead) as observed in ATN1-FL-65Q dentate nucleus cells, often referred to as a multilamellar body. Scale bar, 500 nm

(J) Example of a cell showing multiple accumulations of tertiary lysosomes (red arrowheads) and double-membrane autophagic vesicles (black arrowheads) containing undigested debris frequently observed in ATN1-FL-65Q dentate nucleus cells. Scale bar, 1 μm. Lower-magnification representation of the nucleus is shown in Figure 5A.

(K) Magnification of the inset in (J) showing two double-membrane vesicular structures (black arrowheads), containing undigested material. Scale bar, 500 nm.

(L) High-magnification image of an autophagosome (arrow), as a double-membrane vesicular structure containing several endosomes (full arrowhead), and an undigested former mitochondrion (framed arrowhead). Scale bar, 500 nm.

(M) Representative images of dentate nucleus cells from WT;GFP-LC3, ATN1-FL-26Q;GFP-LC3, and ATN1-FL-65Q;GFP-LC3 end-stage mice evaluated for GFP (green) and LAMP2a (red) positive as well as colocalized (yellow) puncta. Puncta were counted within the soma of cells, showing typical morphology for DN neurons with large low-intensity nuclei (blue) surrounded by a relatively large cytoplasm and a high LAMP2a positive background. Images were taken at the nuclear level of the cell with Axiovert epifluorescence microscope using Apotome optical sectioning with high grid at 100× objective magnification. Scale bar, 10 μm.

(N) Quantification of GFP-positive puncta in dentate nucleus cells of WT;GFP-LC3 (wt), ATN1-FL-26Q;GFP-LC3 (26Q), and ATN1-FL-65Q;GFP-LC3 (65Q) mice at the presymptomatic stage of 3 weeks (wt, n = 83 cells, 3 animals; 26Q, n = 77 cells, 3 animals; 65Q, n = 96 cells, 4 animals) and end-stage (wt, n = 53 cells, 3 animals; 26Q, n = 49 cells, 3 animals; 65Q, n = 52 cells, 3 animals). One-way ANOVA, mean ± SEM, ***$p < 0.001$, **$p < 0.01$.

(O) Relative co-localization ratio of puncta positive for both GFP-LC3 and LAMP2a to puncta positive for only GFP-LC3 in dentate nucleus cells of 3 week and end-stage WT;GFP-LC3 (wt), ATN1-FL-26Q;GFP-LC3 (26Q) and ATN1-FL-65Q;GFP-LC3 (65Q) mice. One-way ANOVA, mean ± SEM, **$p < 0.01$, *$p < 0.05$.

See also Figure S2.

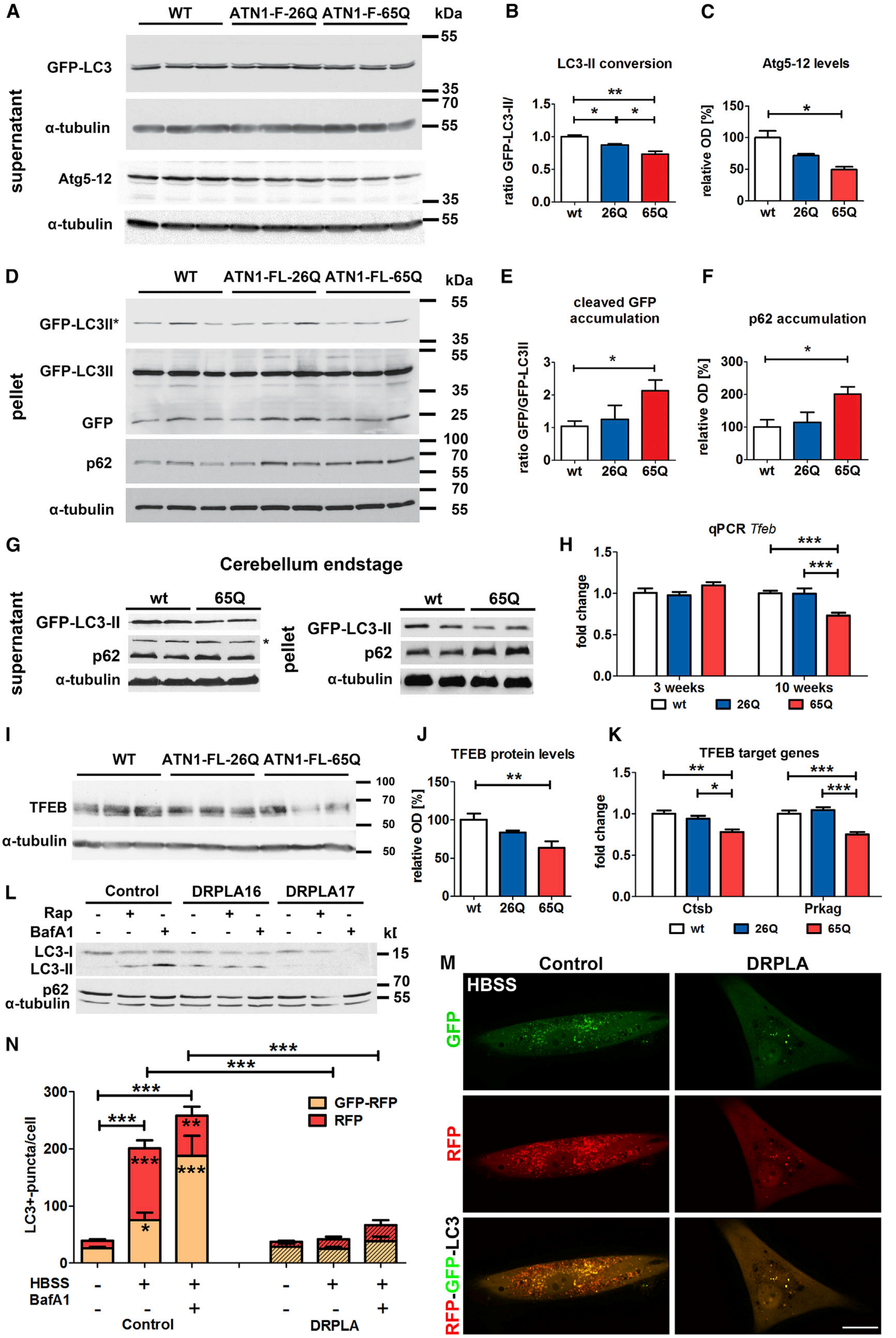

(legend on next page)

Nuclear Degeneration through Nucleophagy-Based Cytoplasmic Displacement of LaminB1 following Block in Canonical Autophagy

In parallel with autophagy stalling, we detected prominent nuclear pathology in DRPLA mice. In DN neurons, there was a striking accumulation of p62 inside the nucleus of the ATN1-FL-65Q mice with the formation of a large inclusion (Figure 6Aiii), which appeared to be composed of smaller aggregates at end-stage (Figure S5A). The abnormal nuclear accumulation of p62 suggests a specific pathological defect in the nucleus. TEM analysis of DN neurons revealed that at end-stage these cells have a strikingly deformed nucleus (Figure 4A; see also Figure 6Ai), with parts bulging out, especially those rich in heterochromatin, and with the appearance of vacuolar-like structures at the periphery. Reviewing previous TEM data, we noticed that nuclear deformation was also present in *Drosophila* DRPLA models and *fat* mutants [14, 15]. Indeed, also in the *Drosophila* central brain, the normal circular organization of LaminB in the cortex of neuronal nuclei is significantly altered and irregular in aged DRPLA flies, with some cells showing ruffles and gaps (Figures 4B and 4C). Most interestingly, human control fibroblasts lose the normal LaminB1 organization and display inward ruffles of the lamin layer when treated with BafA1 for 48 hr (Figure 4D). DRPLA fibroblasts also display LaminB1 inward ruffles when treated with BafA1 and, remarkably, also when treated with Rap for 48 hr (Figure 4D). In addition, whereas both control and DRPLA cells reduce their nuclear size when treated with Rap and BafA1 (Figure 4E), only control fibroblasts increase the roundness of their nucleus, opposite to DRPLA fibroblasts (Figure 4F). This indicates that human DRPLA cells respond differently than control cells to Rap and BafA1 and that chronic blockage of the autophagy-lysosome clearance by BafA1 makes control and patient cells converge toward parameters characteristic of DRPLA nuclei (Figure 4G).

Next, we have tested nuclear shape dynamics in fibroblasts from Vici syndrome (VS) patients. VS is an early-onset infantile multisystem disorder with well-characterized autolysosomal blockage due to mutations in the *EPG5* gene [20, 21], which is required for the autophagosome-lysosome fusion [22]. VS fibroblasts displayed similar nuclear shape dynamics as the DRPLA fibroblasts upon 48-hr BafA1 treatment (Figures S5B and S5C). Interestingly, the age-matched (3 years old) control cells were hardly affected by this treatment, in contrast to the age-matched (51 years old) DRPLA controls, probably reflecting different resistance and plasticity, due to the age difference.

Furthermore, in human neuroblastoma cells SK-N-BE(2) transfected with mCherry-LaminB1, genetic block of autophagy by Atg6 small interfering RNA (siRNA) decreased the roundness of the nucleus (Figures S5D and S5E). A similar trend was displayed after Atg5 knockdown.

We therefore reasoned that defects in nuclear shape dynamics may be linked to the recently reported role of autophagy

Figure 3. Inhibition of Autophagy Flux at Lysosomal Level and Decrease in Autophagy Initiation Signaling in DRPLA

(A–C) The ratio of LC3II to LC3I was used to quantify autophagic flux in western blot analysis of full-length GFP-LC3 in the supernatant fraction of cerebellar lysates at 14 weeks of age (A). The anti-LC3 antibody recognizes a doublet between 35 and 55 kDa (Figure S4B), consistent with GFP-LC3-I (upper) and cleaved GFP-LC3-II (lower). The level of Atg5-12 conjugate was used to quantify the events of autophagy initiation. Densitometric analysis shows a decreased relative abundance of cleaved GFP-LC3-II to full-length GFP-LC3-I (B) in ATN1-FL-65Q;GFP-LC3 (65Q) mice compared to ATN1-FL-26Q;GFP-LC3 (26Q) and WT;GFP-LC3 (wt) mice. Atg5-12 conjugate (C) is also decreased in ATN1-FL-65Q;GFP-LC3 (65Q) compared to WT;GFP-LC3 (wt). Student's t test, mean ± SEM, $^{**}p < 0.01$, $^{*}p < 0.05$.

(D–F) The accumulation of GFP cleavage product and autophagy receptor p62 was analyzed as a measure of autophagy flux blockage in western blot assay of the cerebellar lysates at 14 weeks of age (D). Mouse anti-GFP antibody recognizes only GFP-LC3-II (Figure S4B) and cleaved GFP after longer exposure. *Shorter exposure of anti-GFP signal. Densitometric analysis of the relative abundance of cleaved GFP to GFP-LC3-II (E) as well as the abundance of p62 relative to α-tubulin (F) in WT;GFP-LC3 mice (wt), ATN1-FL-26Q;GFP-LC3 (26Q) and ATN1-FL-65Q;GFP-LC3 (65Q) mice. Student's t test, mean ± SEM, $^{*}p < 0.05$.

(G) Accordingly, western blot analysis of autophagy shows a stall in autophagy flux in end-stage ATN1-FL-65Q mice compared to wild-type (WT) as evidenced by relative decrease of GFP-LC3-II as well as increase of p62 in the pellet fraction. *Anti-p62 antibody revealed an additional band 20 kDa above the expected band at around ~60 kDa in the supernatant fractions of the cerebellum in end-stage mice.

(H) qPCR analysis of *Tfeb* mRNA levels in the cerebellum of wild-type (wt, white), ATN1-FL-26Q (26Q, blue) and ATN1-FL-65Q mice (65Q, red) at presymptomatic (3 weeks) and early symptomatic (10 weeks) time points. Relative levels normalized to β*-actin* and *Hprt1* are given as a fold change of wild-type. Two-way ANOVA, v1, genotype; v2, age, mean ± SEM (n = 6), $^{***}p < 0.001$.

(I and J) Levels of Tfeb protein in the supernatant fraction of cerebellar lysates at 14 weeks of age (I). Densitometric analysis (J) reveals a significant decrease in ATN1-FL-65Q;GFP-LC3 (65Q) compared to WT;GFP-LC3 (wt). One-way ANOVA, mean ± SEM, $^{**}p < 0.05$.

(K) qPCR analysis of *Ctsb and Prkag* mRNA levels in the cerebellum of wild-type (wt, white), ATN1-FL-26Q (26Q, blue) and ATN1-FL-65Q mice (65Q, red) at an early symptomatic (10 weeks) time point. Relative levels normalized to *Hprt1* are given as a fold change of wild-type. One-way ANOVA, mean ± SEM (n = 6), $^{*}p < 0.05$, $^{**}p < 0.01$, $^{***}p < 0.001$.

(L) Whole-cell lysates of human fibroblasts from healthy control and DRPLA patients were subjected to western blot analysis for endogenous LC3-I and LC3-II as well as p62. Induction of autophagy with Rap and block with BafA1 for 6 hr resulted in increase of LC3II compared to DMSO in control fibroblasts, while there was no acute response observed in DRPLA patient samples. No changes in p62 levels were evident after 6 hr acute treatment.

(M and N) Analysis of the autophagy flux in control and DRPLA fibroblasts (DRPLA 17) transfected with the tandem RFP-GFP-LC3B reporter. Starvation in Hank's balanced salt solution (HBSS) medium was used to induce autophagy, BafA1 treatment to inhibit lysosomal degradation. Autophagosomes are marked by yellow signal as a result of combined RFP and GFP double fluorescence. Due to quenching of the GFP signal in acidic environment autolysosomes show RFP fluorescence only. Representative images acquired with Nikon spinning disc confocal microscope display a greater amount of autophagosomes and autolysosomes in control fibroblasts compared to DRPLA after starvation in HBSS for 3 hr (M). Quantification in (N) demonstrates a significant increase in both autophagosomes and autolysosomes in control but not in DRPLA cells after starvation. Addition of BafA1 to starvation medium resulted in a greater number of $GFP^{+}RFP^{+}$ puncta as compared to starvation only in control cells. No changes were significant in DRPLA patient fibroblasts (see also Figure S6H). Significance values in the columns show differences for fed versus starved condition and starved versus +BafA1 condition for RFP^{+} or $GFP^{+}RFP^{+}$ puncta. One-way ANOVA, mean ± SEM, $^{*}p < 0.05$, $^{***}p < 0.001$. Significance values between control and DRPLA cells for overall puncta are shown above the horizontal bars, two-way ANOVA $^{***}p < 0.001$; v1, genotype; v2,- total GFP^{+} and $GFP^{+}RFP^{+}$ puncta. Scale bar, 20 μm.

See also Figures S3 and S4.

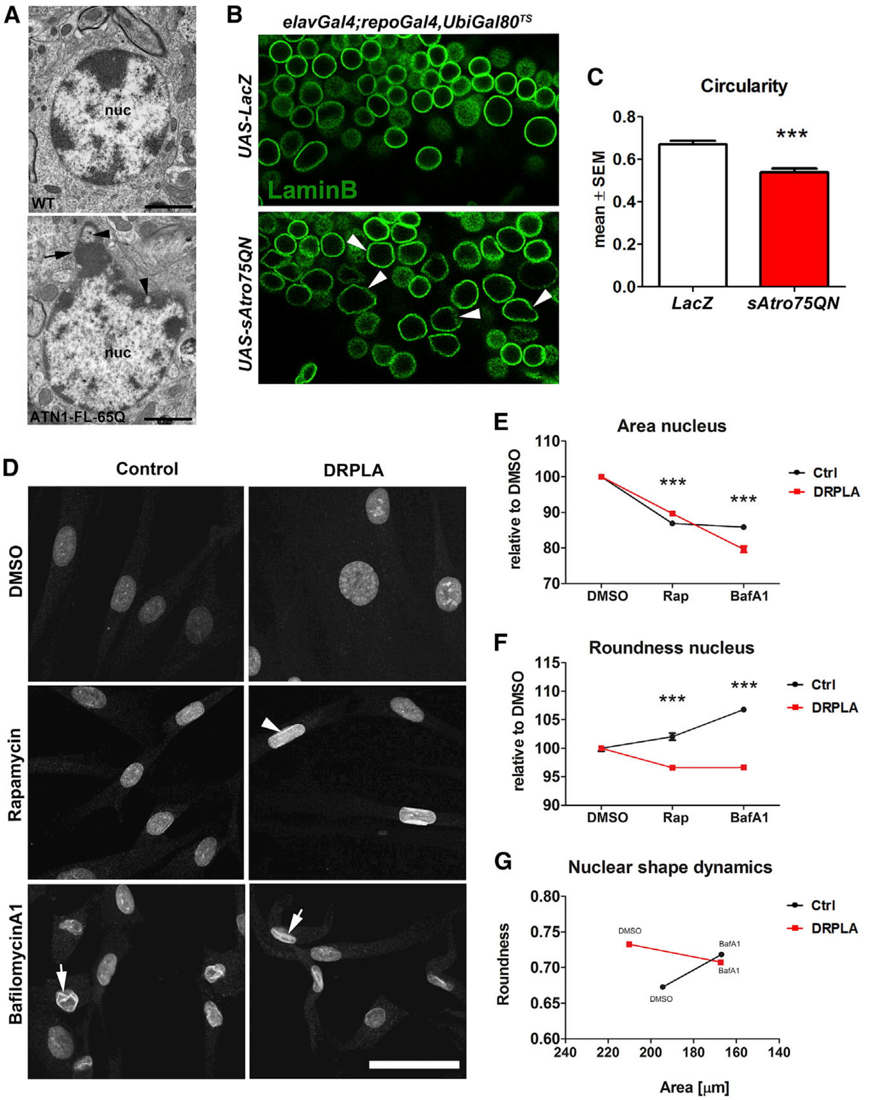

Figure 4. Loss of Nuclear Integrity in Mouse and *Drosophila* Models for DRPLA and in DRPLA Patients' Fibroblast

(A) TEM images of dentate nucleus cells in wild-type (WT, top) and ATN1-FL-65Q (bottom) mice. The latter is displaying irregular shaped nuclei, where the electron dense structures (heterochromatin) are bulging out at the periphery (arrow) with electron-lucent vacuolar-like structures (arrowheads). nuc, nucleus. Scale bar, 5 μm.

(B and C) Overexpression of *Drosophila* polyQ Atrophin and *LacZ* as a control using the UAS-Gal4 system specifically in adult glial and neuronal cells driven by *repo* and *elav* promotors. The expression was induced in fully developed adult flies for 14 days by utilizing ubiquitously expressed temperature sensitive *Gal4* repressor *UbiGal80^{TS}* and inactivated at 29°C. The confocal images of *Drosophila* LaminB show irregular lamina (arrowheads) in *sAtro75QN* overexpressing flies compared to *LacZ* expressing flies (B), which is reflected in significant loss of circularity (ImageJ, particle analysis): mean ± SEM, n = 5, Student's t test ***p < 0.001 (C).

(D–G) Analysis of nuclear shape dynamics in DRPLA (17) patient fibroblasts and age-matched control after 48-hr treatment with Rap and BafA1. Representative images show folding of nuclear envelope revealed by the aLaminB1 antibody upon treatment with BafA1 in both control and DRPLA (arrows), while Rap induced folding (arrowhead) in DRPLA fibroblasts (D). The structural changes were reflected by a decrease in nuclear size after treatment in both control and DRPLA cells (E). While the nuclear roundness decreased in DRPLA cells, it increased in controls (F). The plot of roundness versus area shows an opposite trend and convergence of controls and DRPLA nuclei upon BafA1 treatment (G). Automated quantification was performed using Opera Phenix high-content screening system and Columbus software. Mean ± SEM, n = 5, two-way-ANOVA ***p < 0.001, *p < 0.05; v1, genotype; v2, treatment. Scale bar, 50 μm.

See also Figure S5.

in the degradation of nuclear lamina [8] and have analyzed LaminB1 interaction with the autophagy machinery. In DN neurons, LaminB1 is distributed in speckles of puncta throughout the nucleus. In ATN1-FL-65Q mice, we detected a significant accumulation of larger LaminB1 dots in the cytoplasm (Figures 5A and 5B), which was one of the earliest cellular phenotypes detected at 3 weeks in these mice (Figures S7E and S7H). At end-stage, many of the LaminB1 puncta co-localized with GFP-LC3 (Figures 5A, 5C, and S6A) in particular in the ATN1-FL-65Q line (Figure 5C). While some of the brighter dots localize in close proximity or co-localize directly with p62, also in WT and ATN1-FL-26Q lines, the majority displayed a segregated distribution (Figure S5A). In particular, many more puncta were detected in ATN1-FL-65Q mice, but there was no overall overlap with the intranuclear p62 inclusion (Figure S5A). In contrast, many LaminB1 puncta co-localized with polyQ protein aggregates, mostly inside the nucleus, but also in the cytoplasm in DN neurons (Figure 5D) and Purkinje and granule cells (Figure S6B). Despite the reduced number of polyQ dots in WT and ATN1-FL-26Q lines, some co-localization could be observed also in these cases (Figures S6B and S6C). In granule cells, ATN1-FL-65Q induced complete disorganization of nuclear LaminB1 at end-stage (Figure S6B), whereas at 3 weeks only early signs of incomplete circular organization were detectable (Figure S6C). Interestingly, overexpression of ATN1-FL-26Q at end-stage was sufficient to induce a disorganization of LaminB1 in some cells (e.g., granule cells, Figure S6B), but not in others (e.g., DN neurons).

Neuronal cell degeneration in the ATN1-FL-65Q mice correlates with the increased localization of LaminB1 into the cytoplasm early on. This likely relates to the disruption of the nuclear shape (Figure 5A), the severe alterations in DNA and chromatin appearance (Figure S6E) and the increased levels of γH2AX (Figures S6F and S6G), a marker of senescence and DNA damage [23].

We used human DRPLA fibroblasts for further functional analysis. Here, we also detected a significantly higher number of

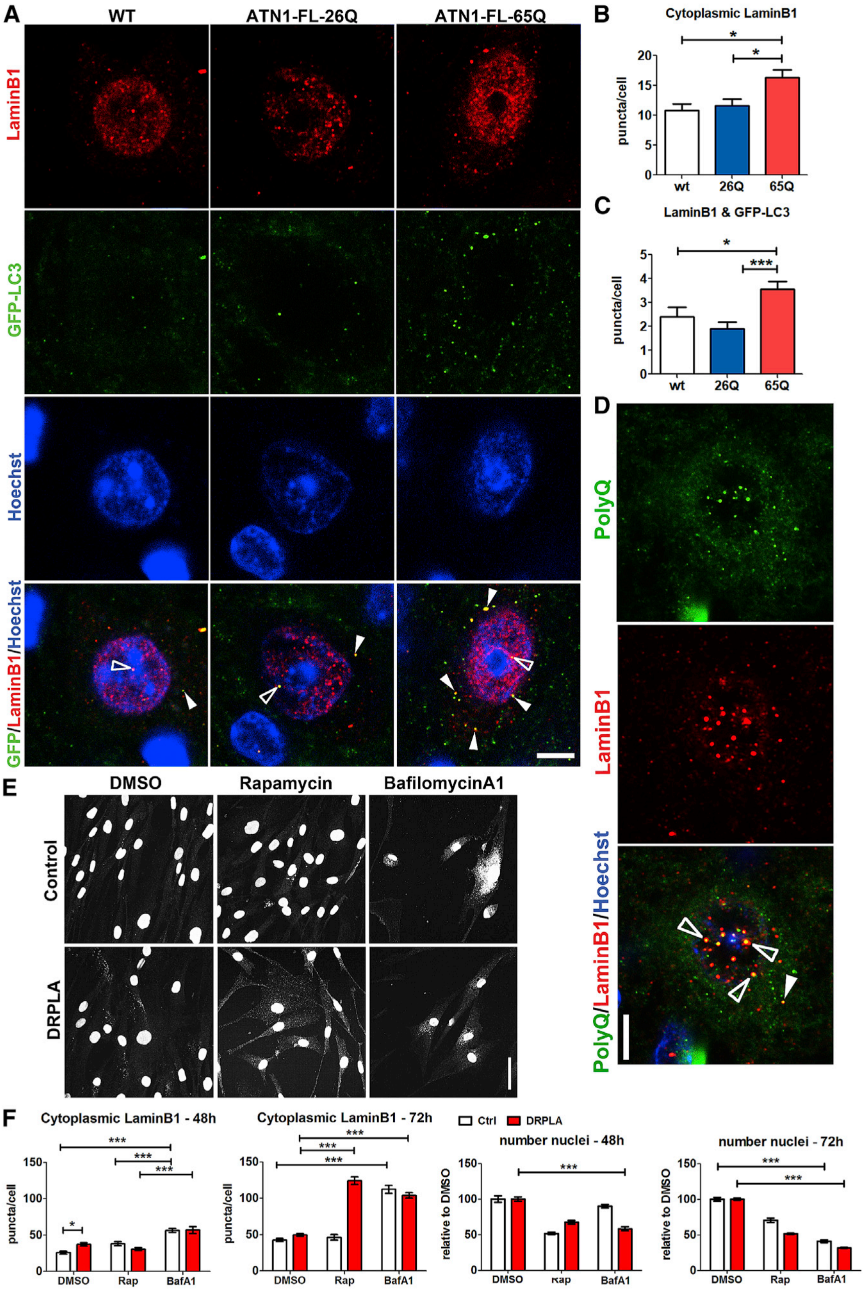

Figure 5. Lamin-Based Nucleophagy Associates with PolyQ Inclusions in DRPLA Mice and Is Aggravated in Human DRPLA Fibroblasts upon Lysosomal Blockage

(A–C) Representative images of dentate nucleus cells from WT;GFP-LC3 (WT, left), ATN1-FL-26Q;GFP-LC3 (middle), and ATN1-FL-65Q;GFP-LC3 (right) end-stage mice show co-localization (yellow) of GFP-LC3 (green) and LaminB1 (red) in the nucleus (framed arrowheads) and, in particular, in the cytoplasm (full arrowheads) of ATN1-FL-65Q cells (A). The nucleus is visualized in blue. Single-plane images were taken with the confocal laser scanning microscope. Quantification indicates a significant increase in the amount of cytoplasmic LaminB1 (B) and of its co-localization with GFP-LC3 (C) in ATN1-FL-65Q;GFP-LC3 mice compared to WT;GFP-LC3 and ATN1-FL-26Q;GFP-LC3. One-way-ANOVA ***$p < 0.001$, *$p < 0.05$. Scale bar, 5 μm.

(D) Some nuclei in ATN1-FL-65Q;GFP-LC3 show strong degeneration reflected in LaminB1 reorganization forming bright intranuclear puncta. Intranuclear (framed arrowheads) and also cytoplasmic (full arrowheads) LaminB1 puncta colocalize with PolyQ positive puncta. Scale bar, 5 μm.

(E and F) Analysis of LaminB1 redistribution into the cytoplasm in DRPLA patient fibroblasts and age-matched control after 48- and 72-hr treatment with Rap and BafA1. Representative images show cells after 72-hr treatment visualized with α-LaminB1 antibody (E). Upon treatment with BafA1 both control (white) and DRPLA (red) fibroblasts show an increase in LaminB1 positive puncta after 48 and 72 hr, while Rap increased cytoplasmic LaminB1 puncta only after 72 hr in DRPLA fibroblasts (F). In line the effect inhibitory effect of mTOR on proliferation, Rap resulted in significant decrease of relative cell number ($p < 0.001$ not shown). BafA1 treatment for 48 hr resulted in decreased cell number only in DRPLA samples in contrast to controls, while after 72 hr there was a significant reduction in cell number in both control and DRPLA samples. Automated quantification was performed using Opera Phenix high-content screening system and Columbus software. Mean ± SEM, two-way-ANOVA ***$p < 0.001$. Scale bar, 50 μm.

See also Figure S6.

cytoplasmic LaminB1 puncta after 48 hr (Figures 5E and 5F). Block of lysosomal clearance with BafA1 strikingly augmented cytoplasmic LaminB1, overpowering any difference between DRPLA and control fibroblasts. Longer culturing for 72 hr led to an overall increase in cytoplasmic LaminB1 eliminating the difference between control and DRPLA fibroblasts, potentially masked by disappearance of more severely affected DRPLA cells. However, 72-hr Rap treatment had an extraordinary effect in DRPLA fibroblasts (not seen in control cells) on the accumulation of cytoplasmic LaminB1 (Figures 5E and 5F). Interestingly, BafA1 treatment had a progressive effect on cell death with control fibroblasts displaying a significant reduction in cell number only after 72 hr (Figure 5F), whereas DRPLA fibroblasts displayed a significant decrease in cell number already at 48 hr (Figure 5F) probably due to exacerbation of an already present blockage in the autophagy-lysosome clearance.

Overall, these data in mice and human cells suggest that a stall in canonical autophagy triggers an accumulation of LaminB1 in the cytoplasm, which correlates with nuclear degeneration in DRPLA mice and cell death in human fibroblasts.

Degradation and Excretion of Cytoplasmic LaminB1 during Cell Degeneration

In Ras-induced cancer models, nucleophagy has been shown to result in the lysosomal degradation of LaminB1 through autophagy [8]. However, given the stall in autophagy in DRPLA or

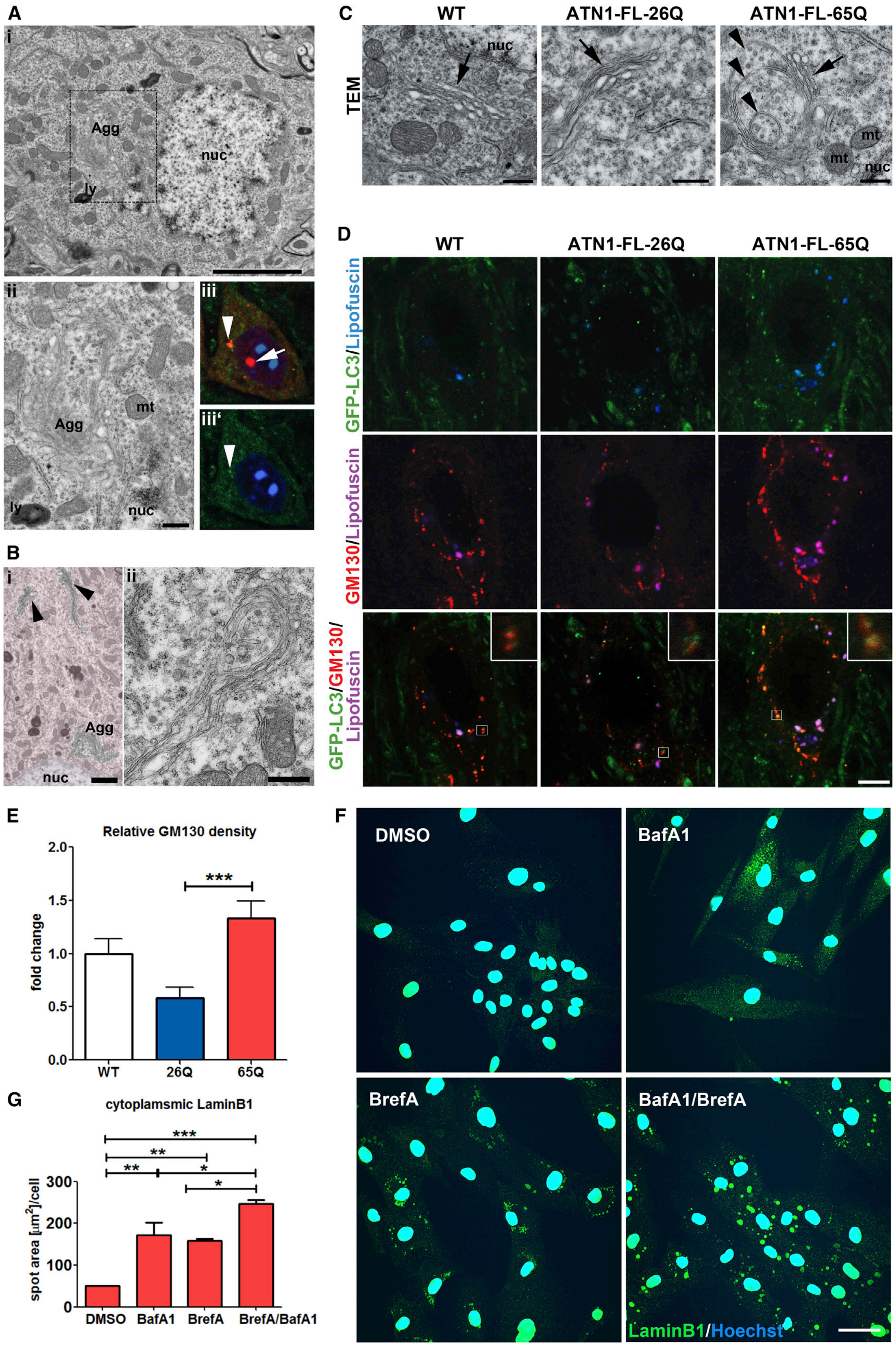

(legend on next page)

following BafA1 treatment, we reasoned that alternative clearance routes may be employed for the LaminB1 accumulated in the cytoplasm.

TEM investigation of ATN1-FL-65Q DN cells also suggests that alternative routes to canonical autophagy may be active in DRPLA. Remarkable perinuclear membranous accumulation (Figure 6A), which surrounded cytoplasmic components enriched with electron dense spots (Figure 6Aii). Similar structures have been referred to as aggresomes [24], which are formed close to the nucleus upon strong autophagy induction in association with p62 and LC3. Indeed, some DN cells formed a perinuclear p62- and GFP-LC3-positive structure (Figure 6Aiii). A general disorganization of intracellular membranes in the ATN1-FL-65Q cells was observed (Figures 2D–2F), also in the distal cytoplasmic regions, where long phagophore-like structures were formed (Figure 6B). Given the abnormal membrane organization, we reasoned that endoplasmatic reticulum (ER) stress could have been induced, also known to induce autophagy [25]. *Bip* and *Chop* are ER-stress-responsive genes that are known to be regulated by all three pathways, *Bip* predominantly by the ATF6 pathway and *Chop* predominantly by the PERK pathway. The splicing of the mRNA encoded by the *Xbp1* gene is selectively regulated by the IRE1 pathway. We find a significant increase of Bip but not spliced Xbp1 and Chop mRNAs in the ATN1-FL-65Q mouse line at pre-symptomatic stages (Figure S7A), but this is not sustained at early symptomatic stages, with the proapoptotic Chop being significantly downregulated (Figure S7B).

We then analyzed at end-stage the other major source of intracellular membranes, the Golgi apparatus, reported to participate in autophagy [26, 27] also as non-canonical Golgi membrane-associated degradation (GOMED) in Atg5- and 7-deficient cells [28, 29]. Relatively large double-membrane vesicular structures, in some cases rather complex with vesicles apparently enclosed in each other (Figure 6C) were evident in the ATN1-FL-65Q line. In addition, the Golgi marker GM130 localized in enlarged tubular structures in the ATN1-FL-65Q DN cells at end-stage, in close proximity to GFP-LC3 puncta (Figure 6D). The whole GM130 compartment displayed an increase in size in end-stage ATN1-FL-65Q DN cells compared to ATN1-FL-26Q and a similar trend toward WT, with a significant shift in the cell distribution toward larger Golgi size (Figures 6E and S7C). This phenotype was not seen at an earlier stage (Figures S7E and S7F); however, the Golgi was significantly, albeit only transiently, more fragmented at 3 weeks in ATN1-FL-26Q and ATN1-FL-65Q mice (Figures S7D, S7E, and S7G). Golgi fragmentation has been associated with induction of non-canonical autophagy [30].

These data indicate an evolution of Golgi pathology from early stages in DRPLA mice. To assess the mechanistic role of the Golgi in the accumulation of cytoplasmic LaminB1, we treated human DRPLA fibroblasts with Brefeldin A (BrefA), which compromises Golgi integrity and function [31]. BrefA treatment per se induced dramatic LaminB1 cytoplasmic accumulation in DRPLA fibroblasts, synergistic to the previously described BafA1 effect (Figures 6F and 6G). Morphologically, in comparison to BafA1, BrefA induced the formation of larger LaminB1 puncta in a circular perinuclear pattern (Figure 6F), suggestive of an organization around cellular organelles rather than diffused cytoplasmic spreading. As in DRPLA, EPG5-deficient VS cells displayed a significant increase in the accumulation of LaminB1 in the cytoplasm with respect to controls in control conditions, as well as under a number of treatments (Figures S7I and S7J). Importantly, a synergic effect of BafA1 with BrefA was observed, while no synergy was displayed between BafA1and Rap.

Overall, these data indicate that the Golgi is involved in the degradation of cytoplasmic LaminB1, through a separate mechanism from that of canonical autophago-lysosomal digestion. Many of these characteristics are shared by the recently described GOMED pathway [29].

However, we find that not all LaminB1 that localizes to the cytoplasm is degraded. In DRPLA fibroblasts, cells rich in cytoplasmic LaminB1 puncta excrete small bodies containing LaminB1 and p62 (Figure 7A). SK-N-BE(2) human neuroblastoma cells, co-transfected with constructs encoding cytoplasmic EGFP and an mCherry-LaminB1 chimeric protein, display similar significant increase in cytoplasmic LaminB1 upon BafA1 treatment (Figures S7K and S7L). In these cells, we were able to visualize LaminB1 exit from the nucleus, trafficking to the cell surface

Figure 6. Aggresome-like Formation and Golgi-Mediated Degradation as Signs of Alternative Autophagy Induction in the Dentate Nucleus of DRPLA Mice

(A) (i) TEM image of a dentate nucleus cell in ATN1-FL-65Q mouse containing a membranous aggresome-like (Agg) accumulation in close proximity to the cell nucleus (nuc). Scale bar, 5 μm. (ii) Higher magnification of the inset in (i) containing the aggresome-like (Agg) membranous formation. Scale bar, 500 nm. ly, lysosome; mt, mitochondrion. (iii and iii') confocal fluorescence image of DN cell in ATN1-FL-65Q;GFP-LC3 mouse brain with p62 positive (red) inclusion inside the nucleus (blue) as well as outside of the nucleus (arrowhead), which also co-localizes with GFP-LC3 (green).

(B) (i) TEM image of a dentate nucleus cell in ATN1-FL-65Q mice; in addition to a large perinuclear aggresome-like (Agg) structure, long membranous formations (arrowhead) with phagophore-like structure are shown at the distal side of the cell soma. Scale bar, 2 μm (note this is a higher-magnification cutout of the image in Figure 2F). (ii) High-magnification TEM image of a similar phagophore-like structure. Scale bar, 300 nm.

(C) Representative TEM images of dentate nucleus cells show double-membrane vesicular structures (arrowheads) in close proximity to the Golgi apparatus (arrow) in ATN1-FL-65Q mice (right), compared to normal Golgi morphology in ATN1-FL-26Q (middle) and wild-type (WT, left) mice. Scale bar, 500 nm

(D and E) Confocal fluorescence microscopy images of dentate nucleus cells showing an enlarged Golgi apparatus (GM130, red) in ATN1-FL-65Q;GFP-LC3 mouse line (left) compared to ATN1-FL-26Q;GFP-LC3 line (middle) and WT;GFP-LC3 (WT, right) (D). Inset in the bottom row shows a higher magnification of a GFP-LC3 (green) positive puncta in close proximity to GM-130 positive structures. Scale bar, 5 μm. Quantification of GM130 signal evidences an increase in the ATN1-FL-65Q;GFP-LC3 mouse line (E). Kruskal-Wallis multiple comparison analysis, mean ± SEM, ***$p < 0.001$

(F and G) Analysis of LaminB1 redistribution into the cytoplasm in DRPLA (17) patient fibroblasts after 48-hr treatment with BafA1 and/or BrefA. BrefA was only added for the last 24 hr. BafA1 and BrefA display an increased localization of LaminB1 into the cytoplasm showing different distribution patterns: diffused after BafA1 treatment only and concentrated in large perinuclear puncta after BrefA treatment. Combined BafA1 and BrefA treatment shows synergistic effect on cytoplasmic LaminB1 localization. Scale bar, 50 μm (F). Automated quantification of area occupied by LaminB1 positive puncta in the cytoplasm was performed using Opera Phenix high-content screening system and Columbus software. Mean ± SEM, two-way ANOVA ***$p < 0.001$, **$p < 0.01$, *$p < 0.05$.

See also Figure S7.

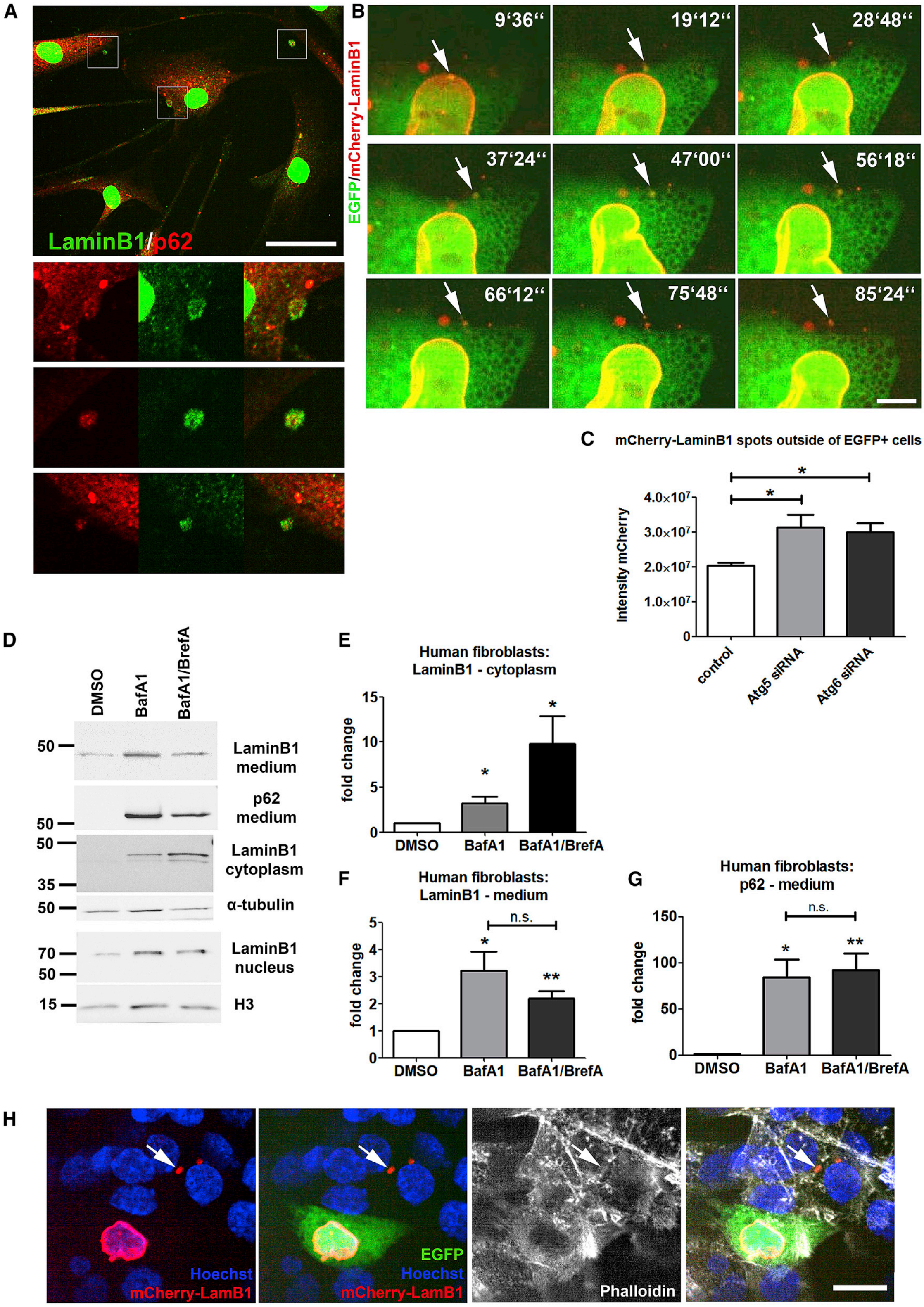

Figure 7. Excretion of LaminB1-Rich Buds following Autophagy and Golgi Impairment

(A) Representative image showing DRPLA (17) fibroblasts after 72 hr Rap treatment showing formation of buds at the plasma membrane containing LaminB1 and p62 puncta. High magnification of insets in the image is shown on the left. Scale bar, 25 μm.

(legend continued on next page)

and expulsion from the cytoplasm (Figure 7B; Movie S3). In this slow excretion process, the LaminB1-containing buds appear to remain in close proximity of the cell for prolonged time after expulsion from the cytoplasm, suggesting a link may be retained (Movie S3). Most interestingly, co-transfection of siRNA against key autophagy genes Atg5 and Atg6 increases the excretion of LaminB1 in these cells (Figure 7C). We subsequently analyzed the culture medium of DRPLA fibroblasts, using ultracentrifugation to further characterize the excretion products. LaminB1 in the medium (and in the cytoplasm) appears as a fragment (Figure 7D), possibly resulting from proteolysis of the full-length LaminB1 upon exit from the nucleus. The excreted LaminB1 fragment also specifically accumulates in the 100,000 × *g* fraction upon ultracentrifugation (Figure S7M), which is known to be enriched in microvescicles and exosomes but not apoptotic bodies. Treatment with BafA1 and BrefA, which significantly increases the amount of LaminB1 in the cytoplasm (Figures 7D and 7E), also facilitates a dramatic increase in LaminB1 and p62 in the culture medium (Figures 7D–7G). BrefA treatment, in combination with BafA1, did not impair significantly the excretion of LaminB1 or p62 (Figure 7F).

Taken together, these data indicate that autophagy inhibition and Golgi impairment increase the amount of LaminB1 and p62 excreted in buds, which share many characteristics of microvesicles.

In neuroblastoma cells in culture, mCherry-LaminB1 puncta can be found inside neighboring non-transfected cells, where they often localize in the perinuclear areas (Figure 7H), reminiscent of the p62 rich aggresome-like structures observed in DRPLA mice (Figure 6A). Thus, adjacent cells might mask the shedding in the complexity of an *in vivo* model.

In conclusion, our data indicate that LaminB1 and associated proteins are exported from the nucleus and then excreted through a mechanism likely to underlie nuclear and cellular atrophy and death upon chronic autophagy inhibition.

DISCUSSION

The final stages of cell dysfunction and death in neurodegenerative diseases and the atrophy of brain tissue remain elusive in most cases. Recently, the nucleus has received much attention as the organelle whose functionality is severely compromised in aggregation-prone neurodegenerative diseases [1, 2]. Mutation in genes encoding nuclear lamina constituents cause Hutchinson-Gilford progeria [32, 33], an accelerated aging syndrome and have been associated with autophagic degradation of nuclear components [5].

The failure of autophagy in neurodegenerative diseases has been extensively studied and proved to be of paramount importance in most cases [6]. Our laboratory has previously developed DRPLA *Drosophila* models, in which we have reported defects in autophagic clearance [14] and transcription of the *fat* cadherin [15]. We have confirmed these defects in DRPLA mice, including transcription of all mouse Fat genes (data not shown). Here, we show accumulation of undigested autophagosomes and autolysosomes (visualized by TEM), as well as lipofuscin, p62, and GFP cleavage product in the ATN1-FL-65Q mice, indicating insufficient autophagic clearance. Progressive accumulation of p62 in the nucleus acted as a faithful biomarker of cell pathology in the different brain areas (data not shown). However, differently than in *Drosophila*, at the behavioral level we observed gender dimorphism in DRPLA mice, with males being affected more strongly. Furthermore, we provide evidence of a significant effect in terms of activity and exploration due to overexpression of a WT form of human ATN1, a possible gain of function effect, which is absent in the 65Q version and in *Drosophila* models. This highlights the importance of the ATN1-FL-26Q line as an additional control in our analysis, which is in accordance with previous transcriptional analysis reporting 19% of gene expression changes in the ATN1-FL-26Q to be in common with the ATN1-FL-65Q mice but not with a mouse models for Huntington's disease [34]. Unsurprisingly, we observe several mild phenotypes in ATN1-FL-26Q mice, reflected in increased GFP-LC3 turnover in absence of p62 accumulation and in the reorganization of LaminB1 in the cerebellar cortex. Significant differences between the two ATN1 lines, such as increased accumulation of lipofuscin, of LC3 puncta at end-stage and cleaved GFP in combination with decreased Tfeb expression and that of two of its targets, however, demonstrate that the stall in canonical autophagy flux are solely attributed to the polyQ expansion in the ATN1. Golgi enlargement at end-stage, increase in the senescence marker γH2AX, nuclear shape alterations, and cytoplasmic LC3-associated accumulation of LaminB1 are all specific signs of alternative autophagy induction and subsequent

(B) Still frames (taken form Movie S3) of confocal live imaging of a SK-N-BE(3) neuroblastoma cell expressing EGFP and mCherry-LaminB1 treated with BafA1 for 48 hr. Arrow points at a LaminB1 punctum detaching from the nucleus and getting slowly excreted over time. Note the misshapen nucleus and LaminB1 infolding. Scale bar, 5 μm.

(C) Analysis of extracellular LaminB1 in SK-N-BE(2) neuroblastoma cells co-expressing EGFP and mCherry-LaminB1 as well as siRNAs against *ATG5* or *ATG6,* genes participating in autophagy and ATG-dependent secretion. Extracellular LaminB1-mCherry puncta were enriched in the surrounding region to EGFP positive cells void of EGFP signal. Automated quantification of mCherry signal intensity was performed using Opera Phenix high-content screening system and Columbus software. Mean ± SEM, one-way-ANOVA *p < 0.05.

(D–G) Analysis of excretion of cellular component in the medium of human DRPLA (17) fibroblasts treated with BafA1 and BrefA as in Figure 6F. The medium supernatant was collected and subjected to ultracentrifugation, while the cells were lysed separating nuclear and cytoplasmic fractions. BafA1 and combined BafA1/BrefA treatment increase the amount of LaminB1 in the cytoplasmic fraction (D and E). BafA1 increased significantly the amount of LaminB1 (D and F) and p62 (D and G) in the medium supernatant fraction collected after 100,000 × *g* ultracentrifugation; addition of BrefA did not significantly reduce the amount of LaminB1 or p62 found in this fraction. Note that the LaminB1 band in cytoplasmic and medium supernatant fraction runs at a lower molecular weight of ~45 kDa as opposed to the expected ~70 kDa in the nuclear fraction, compatible with proteolytic cleavage. Densitometry of medium supernatant fractions was standardized over cytoplasmic tubulin representing the amount of cells and cytoplasm present in the culture. One sample t test, mean ± SEM, **p < 0.01, *p < 0.05.

(H) Representative image of SK-N-BE(2) neuroblastoma cells expressing EGFP, mCherry-LaminB1, and Atg6 siRNA shows the uptake of LaminB1 puncta by neighboring cells. Arrow points at mCherry-LaminB1 dots found inside non-transfected cells. Phalloidin is used to mark the cytoplasmic area of all cells. Scale bar, 10 μm.

See also Figure S7.

nuclear degeneration in ATN1-FL-65Q animals, absent in ATN1-FL-26Q controls.

The analysis of mouse models and human DRPLA cells has additionally revealed that decreased autophagic degradation is accompanied by alterations in canonical autophagy signaling, which suggests a stall in the formation of new autophagosomes, perhaps resulting from a negative feedback loop. Whereas mTor activity displays a tendency toward inhibition, and thus autophagy induction, the phosphorylation of Atg13 by ULK1, the formation of Atg5-12 conjugates, and the rate of LC3-I/II conversion are all downregulated. This is further compounded by downregulation of Tfeb and at least two of its direct targets. Interestingly, human TFEB has been shown to be involved in auto-regulatory feedback loops in starvation-induced autophagy [35]. Human DRPLA fibroblasts also failed to induce formation and maturation of autophagosomes following autophagy modulating treatment, consistently with the recently reported defects in Beclin-1-dependent autophagy induction [36].

In mice, however, the autophagy stall takes place after an initial mild increase in autophagosomes and autolysosomes, possibly driven also by the early moderate ER-stress and Golgi fragmentation. Altogether, our current findings in mice support a model in which neuronal cells challenged by polyQ ATN1 show an overall attempt to induce autophagy but, later on, shut down canonical autophagy signaling, possibly as a result of the inability to progress toward clearance. This suggests that new autophagosomes are not formed through canonical signaling at end-stage, and that the significant accumulation of GFP-LC3 puncta in post-mitotic neurons *in vivo* rather results from lack of digestion and/or alternative routes, including nucleophagy. Human DRPLA fibroblasts *in vitro* do not display the same accumulation of LC3 puncta, presumably because of cell division and of the reduced time window for cell-culture observations.

Recently, alternative autophagy routes, involving some variation of the canonical autophagy steps and signaling, have been discovered, but their role in neurodegenerative diseases has not been described yet.

Here, we detected alternative clearance pathways in DRPLA mice. At end-stage, we observed formation of aggresome-like structures and Golgi enlargement, both cellular correlates for pathological induction of alternative autophagy routes. The aggresomes have been proposed as a cellular pathway for degradation of polyQ [37] and other aggregation-prone proteins that escape canonical autophagy [38]. However, since DN neurons lack a proper microtubule organizing center (data not shown), it is unclear whether these structures also meet the functional definition of aggresomes. The Golgi apparatus, and its trafficking with the ER, is disrupted in several examples of neurodegeneration. However, ER-stress markers, in particular, Chop, are downregulated at later stages in DRPLA, perhaps in an attempt to protect cells from apoptotic responses [39] or as a consequence of lost membrane integrity, incompatible with any further ER-stress induction. Conversely, we provide here the first *in vivo* evidence for GOMED in an autophagy-challenged neuropathology. The Golgi has been reported *in vitro* to engage specifically with autophagy in certain contexts [27, 40], to be a possible source for autophagic membranes in yeast [26] and mammals [41], and to localize in close proximity to phagophores [42]. The involvement of the Golgi in autophagy is, thus, another indication for extreme autophagy stress in the cerebellar nuclei of DRPLA mice. The downregulation of GFP-LC3II conversion and formation of Golgi-associated autophagic structures in DRPLA mice strikingly resemble the alternative autophagy routes described in *Atg5*- and *Atg7*-defective cells and mice [28, 29], consistent with a block in canonical autophagy also in DRPLA models.

We thus propose that, upon stalling of the canonical autophagy machinery, cells attempt to maintain homeostasis via alternative routes like GOMED-like degradation and excretion. This, however, leads to irreversible damage, nuclear breakdown, and to terminal cell atrophy and degeneration.

Here, we report the first *in vivo* evidence of a nucleophagy-based mechanism in neurodegeneration, reflected in the association of nuclear LaminB1 with GFP-LC3 in the nucleus and in the cytoplasm, the cytoplasmic accumulation of LaminB1, its degradation and excretion. This process may be responsible for the collapse of normal nuclear structure, which correlates with the progressive accumulation of p62 in the nucleus and the increase in DNA damage and senescence. Because of the lack of substantial co-localization of the LaminB1 puncta with p62, we interpret the p62 accumulation in the nucleus, as a separate event, which occurs either in parallel or as a consequence of the loss of nuclear integrity, and constitutes a reliable biomarker for cellular pathology (unpublished data). TEM pictures revealed irregular nuclear shapes and morphological changes of nuclear membrane, which may be indications for instability of, and leakage from, the nuclear envelope. This was further backed by the alteration of LaminB structure in both fly and mouse models for DRPLA and in human fibroblasts from DRPLA and VS patients, as well as in human neuroblastoma cells with inhibited autophagy. The export to the cytoplasm of nuclear LaminB1 is likely to underlie the severe defects in nuclear shape, causing disruption of nuclear integrity, which can be sensibly connected to large-scale transcriptional aberrations, nucleo-cytoplasmic transport, and terminal cell degeneration, all key features of human neurodegeneration.

We speculate that in normal cells a low degree of nucleophagy and of LaminB1 excretion might be an attempt at maintaining nuclear health by rejuvenating nuclear lamina and perhaps disposing of other nuclear proteins through autophagy and excretion. In DRPLA, this may be an attempt to counteract the effect of polyQ ATN1. This protein has been reported to interact with nuclear matrix components at discrete locations [43], and we observe a striking co-localization between LaminB1 and polyQ aggregates both in the nucleus and in the cytoplasm.

Once LaminB1 is exported into the cytoplasm, it is cleaved and degraded through two independent pathways, the autophagy-lysosome and GOMED-like systems. A small portion is excreted also in healthy cells. However, when the canonical autophagy-lysosome pathway is impaired, the cytoplasmic LaminB1 is increasingly channeled for excretion, together with p62 in large microvesicles or exophers [44].

We hypothesize that this excretion process is a clearance mechanism for the cell, alternative to lysosomal and Golgi-mediated degradation; however, with the potential consequence of causing cell and tissue atrophy. While the function of the basal excretion of LaminB1-rich buds remains to be understood, the

exacerbated excretion under autophagy and Golgi impairment represents a novel mechanism for cell atrophy and death. In absence of effective degradation and recycling of the basic components, this process depletes the cell of material, damaging its nucleus and cytoplasm and leaving behind cell corpses with a fragile nucleus and thin cytoplasmic layer. In our experiments, *in vivo* and *in vitro*, cytoplasmic accumulation and excretion of LaminB1 correlated with an increase in cell death, misshapen nuclei, and cell atrophy.

The common factor underling this mechanism of cell atrophy and death is a persistent block in autophagy. Since chronic autophagy inhibition has been reported in an increasing number of rare and common human neurodegenerative pathologies, the mechanism here reported may therefore be of great relevance in human disease.

STAR★METHODS

Detailed methods are provided in the online version of this paper and include the following:

- KEY RESOURCES TABLE
- CONTACT FOR REAGENT AND RESOURCE SHARING
- EXPERIMENTAL MODELS AND SUBJECT DETAILS
  - Drosophila
  - Animals
  - Cell culture
- METHOD DETAILS
  - Behavioral analysis
  - Transmission electron microscopy
  - Plasmids and siRNAs
  - Cell culture treatments and autophagy flux analysis
  - Perfusion and tissue processing
  - Immunohistochemistry and cytochemistry
  - Western blot analysis
  - RNA extraction and quantitative RT-PCR
- QUANTIFICATION AND STATISTICAL ANALYSIS
  - Morphometric analysis
  - Statistical analysis

SUPPLEMENTAL INFORMATION

Supplemental Information includes seven figures, one table, and three movies and can be found with this article online at https://doi.org/10.1016/j.cub.2017.10.054.

AUTHOR CONTRIBUTIONS

O.B. helped design the project, designed the experiments, performed the experimental work, collected and analyzed the data, assembled the figures, and wrote the manuscript. A.B. and A.N. performed the experimental work, collected and analyzed the data, and assembled some figures. C.D. and M.S. performed experimental work. I.R. and G.V.-B. helped with experimental work. G.P.B., W.S., H.J., and R.A.F. helped design the experiments and write the manuscript. M.F. designed the project, helped designing the experiments, analyzed the data, and wrote the manuscript.

ACKNOWLEDGMENTS

We thank Fazal Oozeer, Fabian Bangel and Camille Errecart for technical help; Ivo Lieberam, Patrice Codogno, and Deepak Srivastava for reagents and antibodies; Noboru Mizushima for the GFP:LC3 mouse strain; the Wohl Cellular Imaging Centre at King's College London for help with light microscopy; and all members of the Fanto lab for comments and discussions. This work was supported by grants from the Henry Smith Charity (20121109), Ataxia UK (A43), the US National Ataxia Foundation, the Medical Research Council (NIRG-G1002186), and the NC3R (NC/L000199/1) to M.F.; Action Medical Research (2446) to H.J. and M.F.; and the Medical Research Council (G0800846) to G.P.B. This paper is dedicated to the memory of Gaby Schilling.

Received: November 7, 2016
Revised: September 19, 2017
Accepted: October 20, 2017
Published: November 22, 2017

REFERENCES

1. Freibaum, B.D., Lu, Y., Lopez-Gonzalez, R., Kim, N.C., Almeida, S., Lee, K.H., Badders, N., Valentine, M., Miller, B.L., Wong, P.C., et al. (2015). GGGGCC repeat expansion in C9orf72 compromises nucleocytoplasmic transport. Nature *525*, 129–133.
2. Zhang, K., Donnelly, C.J., Haeusler, A.R., Grima, J.C., Machamer, J.B., Steinwald, P., Daley, E.L., Miller, S.J., Cunningham, K.M., Vidensky, S., et al. (2015). The C9orf72 repeat expansion disrupts nucleocytoplasmic transport. Nature *525*, 56–61.
3. Frost, B., Bardai, F.H., and Feany, M.B. (2016). Lamin dysfunction mediates neurodegeneration in tauopathies. Curr. Biol. *26*, 129–136.
4. Chapple, J.P., Bros-Facer, V., Butler, R., and Gallo, J.M. (2008). Focal distortion of the nuclear envelope by huntingtin aggregates revealed by lamin immunostaining. Neurosci. Lett. *447*, 172–174.
5. Park, Y.E., Hayashi, Y.K., Bonne, G., Arimura, T., Noguchi, S., Nonaka, I., and Nishino, I. (2009). Autophagic degradation of nuclear components in mammalian cells. Autophagy *5*, 795–804.
6. Frake, R.A., Ricketts, T., Menzies, F.M., and Rubinsztein, D.C. (2015). Autophagy and neurodegeneration. J. Clin. Invest. *125*, 65–74.
7. Mizushima, N., Levine, B., Cuervo, A.M., and Klionsky, D.J. (2008). Autophagy fights disease through cellular self-digestion. Nature *451*, 1069–1075.
8. Dou, Z., Xu, C., Donahue, G., Shimi, T., Pan, J.A., Zhu, J., Ivanov, A., Capell, B.C., Drake, A.M., Shah, P.P., et al. (2015). Autophagy mediates degradation of nuclear lamina. Nature *527*, 105–109.
9. Nagafuchi, S., Yanagisawa, H., Ohsaki, E., Shirayama, T., Tadokoro, K., Inoue, T., and Yamada, M. (1994). Structure and expression of the gene responsible for the triplet repeat disorder, dentatorubral and pallidoluysian atrophy (DRPLA). Nat. Genet. *8*, 177–182.
10. Kanazawa, I. (1998). Dentatorubral-pallidoluysian atrophy or Naito-Oyanagi disease. Neurogenetics *2*, 1–17.
11. Sato, T., Miura, M., Yamada, M., Yoshida, T., Wood, J.D., Yazawa, I., Masuda, M., Suzuki, T., Shin, R.M., Yau, H.J., et al. (2009). Severe neurological phenotypes of Q129 DRPLA transgenic mice serendipitously created by en masse expansion of CAG repeats in Q76 DRPLA mice. Hum. Mol. Genet. *18*, 723–736.
12. Schilling, G., Wood, J.D., Duan, K., Slunt, H.H., Gonzales, V., Yamada, M., Cooper, J.K., Margolis, R.L., Jenkins, N.A., Copeland, N.G., et al. (1999). Nuclear accumulation of truncated atrophin-1 fragments in a transgenic mouse model of DRPLA. Neuron *24*, 275–286.
13. Ying, M., Xu, R., Wu, X., Zhu, H., Zhuang, Y., Han, M., and Xu, T. (2006). Sodium butyrate ameliorates histone hypoacetylation and neurodegenerative phenotypes in a mouse model for DRPLA. J. Biol. Chem. *281*, 12580–12586.
14. Nisoli, I., Chauvin, J.P., Napoletano, F., Calamita, P., Zanin, V., Fanto, M., and Charroux, B. (2010). Neurodegeneration by polyglutamine Atrophin is not rescued by induction of autophagy. Cell Death Differ. *17*, 1577–1587.
15. Napoletano, F., Occhi, S., Calamita, P., Volpi, V., Blanc, E., Charroux, B., Royet, J., and Fanto, M. (2011). Polyglutamine Atrophin provokes neurodegeneration in *Drosophila* by repressing fat. EMBO J. *30*, 945–958.

16. Simon, P., Dupuis, R., and Costentin, J. (1994). Thigmotaxis as an index of anxiety in mice. Influence of dopaminergic transmissions. Behav. Brain Res. *61*, 59–64.

17. Jalanko, A., and Braulke, T. (2009). Neuronal ceroid lipofuscinoses. Biochim. Biophys. Acta *1793*, 697–709.

18. Mizushima, N., Yamamoto, A., Matsui, M., Yoshimori, T., and Ohsumi, Y. (2004). In vivo analysis of autophagy in response to nutrient starvation using transgenic mice expressing a fluorescent autophagosome marker. Mol. Biol. Cell *15*, 1101–1111.

19. Settembre, C., Di Malta, C., Polito, V.A., Garcia Arencibia, M., Vetrini, F., Erdin, S., Erdin, S.U., Huynh, T., Medina, D., Colella, P., et al. (2011). TFEB links autophagy to lysosomal biogenesis. Science *332*, 1429–1433.

20. Byrne, S., Jansen, L., U-King-Im, J.M., Siddiqui, A., Lidov, H.G., Bodi, I., Smith, L., Mein, R., Cullup, T., Dionisi-Vici, C., et al. (2016). EPG5-related Vici syndrome: A paradigm of neurodevelopmental disorders with defective autophagy. Brain *139*, 765–781.

21. Cullup, T., Kho, A.L., Dionisi-Vici, C., Brandmeier, B., Smith, F., Urry, Z., Simpson, M.A., Yau, S., Bertini, E., McClelland, V., et al. (2013). Recessive mutations in EPG5 cause Vici syndrome, a multisystem disorder with defective autophagy. Nat. Genet. *45*, 83–87.

22. Wang, Z., Miao, G., Xue, X., Guo, X., Yuan, C., Wang, Z., Zhang, G., Chen, Y., Feng, D., Hu, J., and Zhang, H. (2016). The Vici syndrome protein EPG5 is a Rab7 effector that determines the fusion specificity of autophagosomes with late endosomes/lysosomes. Mol. Cell *63*, 781–795.

23. Sedelnikova, O.A., Horikawa, I., Zimonjic, D.B., Popescu, N.C., Bonner, W.M., and Barrett, J.C. (2004). Senescing human cells and ageing mice accumulate DNA lesions with unrepairable double-strand breaks. Nat. Cell Biol. *6*, 168–170.

24. Pankiv, S., Clausen, T.H., Lamark, T., Brech, A., Bruun, J.A., Outzen, H., Øvervatn, A., Bjørkøy, G., and Johansen, T. (2007). p62/SQSTM1 binds directly to Atg8/LC3 to facilitate degradation of ubiquitinated protein aggregates by autophagy. J. Biol. Chem. *282*, 24131–24145.

25. Ogata, M., Hino, S., Saito, A., Morikawa, K., Kondo, S., Kanemoto, S., Murakami, T., Taniguchi, M., Tanii, I., Yoshinaga, K., et al. (2006). Autophagy is activated for cell survival after endoplasmic reticulum stress. Mol. Cell. Biol. *26*, 9220–9231.

26. van der Vaart, A., Griffith, J., and Reggiori, F. (2010). Exit from the Golgi is required for the expansion of the autophagosomal phagophore in yeast Saccharomyces cerevisiae. Mol. Biol. Cell *21*, 2270–2284.

27. Niso-Santano, M., Malik, S.A., Pietrocola, F., Bravo-San Pedro, J.M., Mariño, G., Cianfanelli, V., Ben-Younès, A., Troncoso, R., Markaki, M., Sica, V., et al. (2015). Unsaturated fatty acids induce non-canonical autophagy. EMBO J. *34*, 1025–1041.

28. Nishida, Y., Arakawa, S., Fujitani, K., Yamaguchi, H., Mizuta, T., Kanaseki, T., Komatsu, M., Otsu, K., Tsujimoto, Y., and Shimizu, S. (2009). Discovery of Atg5/Atg7-independent alternative macroautophagy. Nature *461*, 654–658.

29. Yamaguchi, H., Arakawa, S., Kanaseki, T., Miyatsuka, T., Fujitani, Y., Watada, H., Tsujimoto, Y., and Shimizu, S. (2016). Golgi membrane-associated degradation pathway in yeast and mammals. EMBO J. *35*, 1991–2007.

30. Naydenov, N.G., Harris, G., Morales, V., and Ivanov, A.I. (2012). Loss of a membrane trafficking protein αSNAP induces non-canonical autophagy in human epithelia. Cell Cycle *11*, 4613–4625.

31. Klausner, R.D., Donaldson, J.G., and Lippincott-Schwartz, J. (1992). Brefeldin A: Insights into the control of membrane traffic and organelle structure. J. Cell Biol. *116*, 1071–1080.

32. De Sandre-Giovannoli, A., Bernard, R., Cau, P., Navarro, C., Amiel, J., Boccaccio, I., Lyonnet, S., Stewart, C.L., Munnich, A., Le Merrer, M., and Lévy, N. (2003). Lamin a truncation in Hutchinson-Gilford progeria. Science *300*, 2055.

33. Eriksson, M., Brown, W.T., Gordon, L.B., Glynn, M.W., Singer, J., Scott, L., Erdos, M.R., Robbins, C.M., Moses, T.Y., Berglund, P., et al. (2003). Recurrent de novo point mutations in lamin A cause Hutchinson-Gilford progeria syndrome. Nature *423*, 293–298.

34. Luthi-Carter, R., Strand, A.D., Hanson, S.A., Kooperberg, C., Schilling, G., La Spada, A.R., Merry, D.E., Young, A.B., Ross, C.A., Borchelt, D.R., and Olson, J.M. (2002). Polyglutamine and transcription: Gene expression changes shared by DRPLA and Huntington's disease mouse models reveal context-independent effects. Hum. Mol. Genet. *11*, 1927–1937.

35. Settembre, C., De Cegli, R., Mansueto, G., Saha, P.K., Vetrini, F., Visvikis, O., Huynh, T., Carissimo, A., Palmer, D., Klisch, T.J., et al. (2013). TFEB controls cellular lipid metabolism through a starvation-induced autoregulatory loop. Nat. Cell Biol. *15*, 647–658.

36. Ashkenazi, A., Bento, C.F., Ricketts, T., Vicinanza, M., Siddiqi, F., Pavel, M., Squitieri, F., Hardenberg, M.C., Imarisio, S., Menzies, F.M., and Rubinsztein, D.C. (2017). Polyglutamine tracts regulate beclin 1-dependent autophagy. Nature *545*, 108–111.

37. Waelter, S., Boeddrich, A., Lurz, R., Scherzinger, E., Lueder, G., Lehrach, H., and Wanker, E.E. (2001). Accumulation of mutant huntingtin fragments in aggresome-like inclusion bodies as a result of insufficient protein degradation. Mol. Biol. Cell *12*, 1393–1407.

38. Wong, E., Bejarano, E., Rakshit, M., Lee, K., Hanson, H.H., Zaarur, N., Phillips, G.R., Sherman, M.Y., and Cuervo, A.M. (2012). Molecular determinants of selective clearance of protein inclusions by autophagy. Nat. Commun. *3*, 1240.

39. Penas, C., Font-Nieves, M., Forés, J., Petegnief, V., Planas, A., Navarro, X., and Casas, C. (2011). Autophagy, and BiP level decrease are early key events in retrograde degeneration of motoneurons. Cell Death Differ. *18*, 1617–1627.

40. Joachim, J., Jefferies, H.B., Razi, M., Frith, D., Snijders, A.P., Chakravarty, P., Judith, D., and Tooze, S.A. (2015). Activation of ULK Kinase and Autophagy by GABARAP Trafficking from the Centrosome Is Regulated by WAC and GM130. Mol. Cell *60*, 899–913.

41. Ge, L., Melville, D., Zhang, M., and Schekman, R. (2013). The ER-Golgi intermediate compartment is a key membrane source for the LC3 lipidation step of autophagosome biogenesis. eLife *2*, e00947.

42. Biazik, J., Ylä-Anttila, P., Vihinen, H., Jokitalo, E., and Eskelinen, E.L. (2015). Ultrastructural relationship of the phagophore with surrounding organelles. Autophagy *11*, 439–451.

43. Wood, J.D., Nucifora, F.C., Jr., Duan, K., Zhang, C., Wang, J., Kim, Y., Schilling, G., Sacchi, N., Liu, J.M., and Ross, C.A. (2000). Atrophin-1, the dentato-rubral and pallido-luysian atrophy gene product, interacts with ETO/MTG8 in the nuclear matrix and represses transcription. J. Cell Biol. *150*, 939–948.

44. Melentijevic, I., Toth, M.L., Arnold, M.L., Guasp, R.J., Harinath, G., Nguyen, K.C., Taub, D., Parker, J.A., Neri, C., Gabel, C.V., et al. (2017). C. elegans neurons jettison protein aggregates and mitochondria under neurotoxic stress. Nature *542*, 367–371.

45. Horikawa, I., Fujita, K., Jenkins, L.M., Hiyoshi, Y., Mondal, A.M., Vojtesek, B., Lane, D.P., Appella, E., and Harris, C.C. (2014). Autophagic degradation of the inhibitory p53 isoform Δ133p53α as a regulatory mechanism for p53-mediated senescence. Nat. Commun. *5*, 4706.

46. Joffre, C., Dupont, N., Hoa, L., Gomez, V., Pardo, R., Gonçalves-Pimentel, C., Achard, P., Bettoun, A., Meunier, B., Bauvy, C., et al. (2015). the pro-apoptotic STK38 kinase is a new beclin1 partner positively regulating autophagy. Curr. Biol. *25*, 2479–2492.

47. Pampliega, O., Orhon, I., Patel, B., Sridhar, S., Díaz-Carretero, A., Beau, I., Codogno, P., Satir, B.H., Satir, P., and Cuervo, A.M. (2013). Functional interaction between autophagy and ciliogenesis. Nature *502*, 194–200.

48. Kilkenny, C., Browne, W.J., Cuthill, I.C., Emerson, M., and Altman, D.G. (2010). Improving bioscience research reporting: The ARRIVE guidelines for reporting animal research. PLoS Biol. *8*, e1000412.

49. Mangiarini, L., Sathasivam, K., Seller, M., Cozens, B., Harper, A., Hetherington, C., Lawton, M., Trottier, Y., Lehrach, H., Davies, S.W., and

Bates, G.P. (1996). Exon 1 of the HD gene with an expanded CAG repeat is sufficient to cause a progressive neurological phenotype in transgenic mice. Cell *87*, 493–506.

50. Rattray, I., Smith, E., Gale, R., Matsumoto, K., Bates, G.P., and Modo, M. (2013). Correlations of behavioral deficits with brain pathology assessed through longitudinal MRI and histopathology in the R6/2 mouse model of HD. PLoS ONE *8*, e60012.

51. Rattray, I., Smith, E.J., Crum, W.R., Walker, T.A., Gale, R., Bates, G.P., and Modo, M. (2013). Correlations of behavioral deficits with brain pathology assessed through longitudinal MRI and histopathology in the R6/1 mouse model of Huntington's disease. PLoS ONE *8*, e84726.

52. Schnell, S.A., Staines, W.A., and Wessendorf, M.W. (1999). Reduction of lipofuscin-like autofluorescence in fluorescently labeled tissue. J. Histochem. Cytochem. *47*, 719–730.

53. Xu, R., Greening, D.W., Rai, A., Ji, H., and Simpson, R.J. (2015). Highly-purified exosomes and shed microvesicles isolated from the human colon cancer cell line LIM1863 by sequential centrifugal ultrafiltration are biochemically and functionally distinct. Methods *87*, 11–25.

54. Del Campo, M., Oliveira, C.R., Scheper, W., Zwart, R., Korth, C., Müller-Schiffmann, A., Kostallas, G., Biverstal, H., Presto, J., Johansson, J., et al. (2015). BRI2 ectodomain affects Aβ42 fibrillation and tau truncation in human neuroblastoma cells. Cell. Mol. Life Sci. *72*, 1599–1611.

STAR★METHODS

KEY RESOURCES TABLE

REAGENT or RESOURCE	SOURCE	IDENTIFIER
Antibodies		
rabbit α-Lamp2a	Abcam	Cat#ab18528; RRID: AB_775981
mouse α-p62	Abnova	Cat#H00008878-M01; RRID: AB_548364
rabbit α-LaminB1	Abcam	Cat#ab16048; RRID: AB_10107828
mouse α-GM130	BD Transduction Lab,	Cat#010823; RRID: AB_398142
mouse α-PolyQ	Chemicon	Cat#MAB1574; RRID: AB_94263
mouse α-dmLaminB	DSHB	Cat#ADL67.10; RRID: AB_528336
mouse α-γ-H2AX	Millipore	Cat#05-636; RRID: AB_309864
rabbit α-TFEB	Bethyl Labs	Cat#A303-673A; RRID: AB_11204751
rabbit α-Atg5	Novus Biological	Cat#NB110-53818; RRID: AB_828587
rabbit α-Atg13-P-S318	Rockland	Cat#600-401-C49S (Lot 27919); RRID: AB_11181153
rabbit α-phospho-p70S6K	Cell Signaling	Cat#9205S; RRID: AB_330944
mouse α–α-tubulin	SIGMA	Cat#T9026; RRID: AB_477593
chicken α-MAP2	Abcam	Cat#ab5392; RRID: AB_2138153
Chemicals, Peptides, and Recombinant Proteins		
Bafilomycin A1	SIGMA	Cat#B1793
Rapamycin	Calbiochem	Cat#553210
Brefeldin A	SIGMA	Cat#B7651
Lipofectamine3000 reagent	Invitrogen	Cat#L3000-008
TRI Reagent	Invitrogen	Cat#T9424
SuperScript III Reverse Transcriptase	Invitrogen	Cat#18080-051
Critical Commercial Assays		
UPL library	Roche	N/A
TaqMan Universal PCR Master mix	ThermoFisher Scientific	Cat#4304437
SuperSignal West Pico Chemiluminescent Substrate	ThermoFisher Scientific	Cat#34080
Experimental Models: Cell Lines		
SK-N-BE(2)	ATCC	ATTC: CRL-2271
Experimental Models: Organisms/Strains		
Mouse: C3;B6-Tg(Prnp-ATN1)84Dbo/Mmmh	MMRRC repository	RRID: MMRRC_000396-MU
Mouse: C3;B6-Tg(Prnp-ATN1)150Dbo/Mmmh	MMRRC repository	RRID: MMRRC_000398-MU
Mouse: B6CBAF1/OlaHsd	Harlan Olac, Bicester, UK	N/A
Mouse: B6.Tg(CAG-GFP-LC3)	[18]	MTA G. Bates
D. melanogaster: Elav-Gal4;Repo-Gal4,ubi-Gal80^{ts}	This paper	N/A
D. melanogaster: UAS-LacZ	BDSC	#8529
D. melanogaster: UAS-sAtro^{75QN}.	[14]	N/A
Epg5 p.Phe1604Glyfs*20 fibroblasts	[21]	N/A
Human fibroblasts DRPLA17	Corriell	Coriell: GM13717
Human fibroblasts DRPLA16	Corriell	Coriell: GM13716
Human fibroblasts control, m, 51	MRC CNMD Biobank London	UN3373
Human fibroblasts control, m, 3	[21]	N/A
Oligonucleotides		
Atg5 siRNA (5′-GGU UUG GAC GAA UUC CAA CUU GUU U-3′)	Eurofins Genomix; [45]	N/A
Atg6 siRNA (5′-ACA GUG AAU UUA AAC GAC AGC AGC U-3′)	Eurofins Genomix; [45]	N/A

(*Continued on next page*)

Continued

REAGENT or RESOURCE	SOURCE	IDENTIFIER
non-specific control siRNA (5′-AGG UAG UGU AAU CGC CUU G-3′, 47%CG)	Eurofins Genomix	N/A
Primer: *Hprt1* Forward: cctcctcagaccgcttttt Reverse: aacctggttcatcat cgctaa UPL: 95	This paper	N/A
Primer: *β-actin* Forward: aaggccaaccgtgaaaagat Reverse: gtggtacgac cagaggcatac UPL: 56	This paper	N/A
Primer: *Tfeb* Forward: gagctgggaatgctgatcc Reverse: gggacttctgca ggtcctt UPL: 22	This paper	N/A
Primer: *Hprt1* Forward: tgatagatccattcctatgactgtaga Reverse: aagacat tctttccagttaaagttgag UPL: 22	This paper	N/A
Primer: *Ctsb* Forward: cttgctgtggtatccagtgtg Reverse: cacctgaaacca ggcctttt UPL: 50	This paper	N/A
Primer: *Prkag* Forward: ctgtcagacatcctgcaagc Reverse: ctacattcacgg cggtcat UPL: 62	This paper	N/A
Primer: *Bip* Forward: gccaactgttgtaacaatcaaggtct Reverse: tgacttcaatct ggggaactc	This paper	N/A
Primer: *Chop* Forward: tccgcagcaggtgcag Reverse: tcctcataccaggctt cca	This paper	N/A
Primer: *Xbp1* Forward: gccaactgttgtaacaatcaaggtct Reverse: ccaacttg tccagaatgccc	This paper	N/A
Recombinant DNA		
p(RFP)-EGFP-LC3B	[46]	N/A
mCherry-LaminB1-10	gift from Michael Davidson	Addgene plasmid: #*55069*
Software and Algorithms		
EthoVision 7XT	Noldus, Netherlands	N/A
Green and Red Puncta Colocalization macro, ImageJ	[47]	http://imagejdocu.tudor.lu/doku.php?id=plugin:analysis:colocalization_analysis_macro_for_red_and_green_puncta:start
Columbus	PerkinElmer, Hamburg	N/A
Image Studio Lite	Li-Cor	https://www.licor.com/bio/products/software/image_studio_lite/
GraphPad Prism	GraphPad Software	https://www.graphpad.com/how-to-buy/

CONTACT FOR REAGENT AND RESOURCE SHARING

Further information and requests for resources and reagents should be directed to and will be fulfilled by the Lead Contact, Manolis Fanto (manolis.fanto@kcl.ac.uk).

EXPERIMENTAL MODELS AND SUBJECT DETAILS

Drosophila

The following mutant fly stocks were used: *Elav-Gal4, Repo-Gal4, ubi-Gal80^{ts}, UAS-LacZ, UAS-sAtro^{75QN}*. *Elav-Gal4;Repo-Gal4,ubi-Gal80^{ts}* virgin females were crossed to males from the *UAS-LacZ* and *UAS-sAtro^{75QN}* stocks. After development at 18°C the F1 progeny was transferred to 29°C. 14 days old females were used for immunofluorescence.

Animals

All experimental procedures involving mice were carried out under a license from the Home Office according to regulations set by the Animals (Scientific Procedures) Act 1986 (ASPA). Reporting of animal experiments is in accordance to the ARRIVE guidelines [48]. The two DRPLA mouse strains C3;B6-Tg(Prnp-ATN1)84Dbo/Mmmh (26Q) and C3;B6-Tg(Prnp-ATN1)150Dbo/Mmmh (65Q) [12] were recovered from the MMRRC repository and maintained by backcrossing to (CBA/Ca x C57BL/6J)F1 animals, B6CBAF1/OlaHsd (Harlan Olac, Bicester, UK), often used to analyze behavior in the R6/2 HD mouse models [49]. The double mutants heterozygous for

GFP-LC3 and DRPLA were achieved by crossing ATN1-FL-26Q-84 and ATN1-FL-65Q-150 lines to the B6.Tg(CAG-GFP-LC3) [18] autophagy reporter line. Genotyping was performed by PCR using ear biopsies. Primers: PrP- FW: 5′-CTCTTTGTGACTATGTG GACTGATGTCGG-3′, PrP-RV: 5′-GTGGATACCCCCTCCCCCAGCCTAGACC-3′, At-3818: 5′-GGTGGGGAGGTGGCGAGGAT-3′, GFP(LC3): 5′-TCCTGCTGGAGTTCGTGACCG-3′, and LC3*rc3: 5′-TTGCGAATTCTCAGCCGTCTTCATCTCTCTCTCGC-3′. PCR conditions were 95°C for 30 s, 58°C for 30 s, and 72°C for 60 s for a total of 34 cycles. Animals were housed under 12 h light/ 12 h dark cycle (7am to 7pm light), with unlimited access to water and food (Special Diet Service, Witham, UK) in a conventional unit. All animals were immunocompetent and drug naive. Cages were environmentally enriched with a cardboard hut, tube, and bale of shredded paper. The wild-type, ATN1-FL-26Q, and ATN1-FL-65Q were kept in separate cages with 4-6 animals per cage. 6 groups of mice with 8-10 animals per genotype and gender were exposed to repetitive behavioral testing from four weeks of age in the hours 13:00–17:00 on same days of the week. The double-, single- and non-transgenic animals were selected from the same litters, hereby preferably selecting the wild-type control littermates from the heterozygous ATN1-FL-65Q to GFP-LC3 crosses. Grip strength and rotarod performance were tested every four weeks. Open-field experiments were performed at 10 weeks for both sexes as well as at 14 weeks for females only. The mice were separated in two rounds consisting of 13 experimental units for rotarod performance. Females were tested prior to males. For open field the mice were randomly distributed into the four experimental arenas with all three genotypes represented at a time. The behaviorally assessed animals were repurposed for further phenotyping procedures, such as morphological and biochemical analysis after reaching the end-stage (Supplemental table S1). The end-stage was defined as 20 weeks for males and 24 weeks for females using the distress scale.

Cell culture

Human fibroblasts DRPLA17 (Corriell GM13717, male, age 47, 65Q), DRPLA16 (Corriell GM13716, male, age 15, 68Q), age matched control to DRPLA17 (male, age 51), Vici syndrome fibroblasts (Epg5 p.Phe1604Glyfs*20, male, age 3), age matched controls to Vici syndrome (male, age 3) [21] are derived from skin biopsies. Human neuroblastoma cell line SK-N-BE(2) was purchased from ATTC (CRL-2271). This brain neuroblasts are derived originally from a bone marrow metastasis of 2 year old male individual. All cells were cultured in DMEM high glucose with 10% (v/v) FCS, 1 mM sodium-pyruvate, penicillin/ streptomycin and L-Glutamine.

METHOD DETAILS

Behavioral analysis

Rotarod performance

Motor coordination was tested on an accelerating rotarod, by placing mice individually on an accelerating, rotating beam (4-40 rpm) for a maximum of five min as previously described [50]. Latency to fall was recorded as time (sec) the mouse was able to remain on the beam. Mice were exposed consecutively to three trials per day with two habituation and two experimental days at four weeks age and one habituation and two experimental days for following stages. The mice were separated in two rounds consisting of 13 experimental units. Animals of each unit were of the same genotype and age housed in one numbered cage. The experimenter was blind to the genotype of the experimental animals and units coded by numbers. Females were tested prior to males. The apparatus was thoroughly cleaned using 70% ethanol after each trial. The averages from replicates from three experimental trials and two experimental days were calculated for each animal and subjected to statistical analysis (n = 8-10).

Grip strength

Grip strength capacity was assessed as previously described on the habituation day prior to the rotarod performance analysis [51]. Three independent measurements of the strength of the forelimbs only, as well as fore- and hind limbs taken together were averaged as technical replicates. Mice were guided along wire-mesh grid attached to a grip strength monitor (Bioseb *In Vivo* Research Instruments) by holding them at the base of the tail. Mice were either allowed to grip with forelimbs only, or fore- and hind limbs together. The maximum tension was recorded (g) by gently pulling the mouse away from the apparatus. Females were tested prior to males. The experimenter was blind to the genotype of the experimental animals coded by numbers. The averages from replicates of the three experimental trials were calculated for each animal and subjected to statistical analysis (n = 8-10).

Open field

The locomotive behavior was analyzed by placing mice individually into square, plain white open field arenas (50 × 50 × 50 cm, Engineering & Design Plastics, Cambridge, UK) for 30 min. The spatiotemporal position of the individual animal was recorded through a video camera positioned above the apparatus and respective individual trails were tracked and later analyzed using EthoVision 7XT software (Noldus, Netherlands) as previously described [51]. Exploratory activity in a novel, unfamiliar environment was assessed as the distance traveled (cm) over the total area in the first five min after introduction to the open field. Thigmotaxis, the time (s) spent in the peripheral, outer-zone of an open field is indicative of an anxiety-like behavior [16], and was analyzed likewise during the first five min after introduction to the novel environment. The open field arena was divided into two square zones, with outer zone 50 × 50 cm and inner zone with 40 × 40 cm. The general activity was analyzed between five and 25 min after the introduction to the open field as a distance traveled (cm) during the given 20 min interval. The mice were randomly distributed into the four experimental arenas with all three genotypes represented at a time. The open field arena was thoroughly cleaned using 70% ethanol after each trial.

Gait analysis

For the gait analysis non-toxic finger paint was applied on the front (blue) and back (red) paws of the mice and the mice were allowed to walk through a transparent corridor (5 cm x 1 m) toward a dark chamber.

Transmission electron microscopy

The single transgenic DRPLA mice were transcardially perfused with physiological saline solution followed by filtered 2.5% (v/v) EM grade glutaraldehyde and 2% (v/v) EM grade paraformaldehyde in 0.1cM sodium cacodylate buffer. The dissected brains were post-fixed for 24 h and kept at 4°C in 0.1cM sodium cacodylate buffer until further processing. 120 μm thick vibratome slices were post-fixed in 1% (v/v) osmium tetroxide in 0.1cM sodium cacodylate buffer, followed by en bloc contrasting in 1% (w/v) uranyl acetate for 45 min. The coronal brain slices were gradually dehydrated in ethanol and propylene oxide and finally infiltrated and embedded in TAAB embedding resin premix (T028H). The resin embedded slices were trimmed to expose the anatomic region corresponding to the dentate nucleus and processed at the microtome to retrieve gold sections (~90 nm). The ultrathin sections were additionally contrasted with uranyl acetate and lead citrate and viewed on a FEI Tecnai T12 electron microscope operated at 120kV. Images were acquired with an AMT 16000M camera.

Plasmids and siRNAs

p(RFP)-EGFP-LC3B (gift from P. Codogno), *pEGFP-N1*, *mCherry-LaminB1-10 (Addgene, #55069)*. The previously published [45] Atg5 siRNA (5′-GGU UUG GAC GAA UUC CAA CUU GUU U-3), Atg6 siRNA (5′-ACA GUG AAU UUA AAC GAC AGC AGC U-3′) and non-specific control siRNA (5′-AGG UAG UGU AAU CGC CUU G-3′, 47%CG) were obtained from Eurofins Genomix and lyophilized in supplied RNAmax buffer.

Cell culture treatments and autophagy flux analysis

Treatment with 1μM Rap, 10 nM BafA1 and 0.1% of solvent DMSO, respectively, were performed for 6, 24, 48 or72 hr for assessment of autophagy flux. For analysis of LaminB1 accumulation and end excretion cells were treated for 24 hr with DMSO or BafA1, followed by replacement of the medium with DMSO, BafA1, 1 μM BrefA or BafA1/BrefA combination after rinsing with PBS. Cells were fixed in 4% paraformaldehyde for 15 min and washed in PBS. The immunocytochemical evaluation was performed in 96-well plate format using Opera Phenix system and Columbus software. Cells were transiently transfected using Lipofectamine3000 reagent (Invitrogen) according to the manufactures protocol. For assessment of autophagy flux fibroblasts were starved in HBSS or treated with 10 nM BafA1 for 3 hr and analyzed with life imaging using Nikon Spining disc confocal microscope.

Perfusion and tissue processing

The wild-type, single transgenic or double transgenic mice (n = 6 per group) were transcardially perfused with ice cold physiological saline solution followed by filtered 4% (w/v) paraformaldehyde in phosphate buffered saline (PBS). The dissected brains were post-fixed overnight, cryopreserved in 30% (w/v) sucrose in PBS, and embedded in Tissue-Tek O.C.T. Compound (Sakura Finetek). Serial coronal free floating cryosections were collected at 30 μm thickness using Microm cryostat (Thermo Scientific), and stored in anti-freeze solution (40% (v/v) PBS, 30% (v/v) Glycerol and 30% (v/v) Ethylenglycol) at −20°C until further processing.

Immunohistochemistry and cytochemistry

To avoid nonspecific binding and for permeabilization the sections or fixed cells were incubated in blocking solution (5% (v/v) normal goat serum, 1% (w/v) bovine serum albumin (BSA), 0.5% (v/v) Triton X-100 in PBS). For some mouse antibodies additional blocking of endogenous mouse IgGs with 0.1 mg/ml of α-mouse IgG Fab fragments (Zenon Kit, Invitrogen) was necessary. The antibodies were incubated in carrier solution (1% (v/v) normal goat serum, 1% (w/v) bovine serum albumin (BSA), 0.5% (v/v) Triton X-100 in PBS). Incubation of primary antibodies was performed for 48 hr at 4°C on an orbital shaker. Secondary antibodies were applied for 4 hr at room temperature. Primary antibodies: mouse α-GFP (1:500, Roche), rabbit α-GFP (1:5000, Life technologies, A11122), rabbit α-Lamp2a (1:2000, Abcam, ab18528), mouse α-p62 (1:2000, Abnova, H00008878-M01), rabbit α-LaminB1 (1:1000, Abcam, ab16048), mouse α-GM130 (BD Transduction Lab, 010823), mouse α-PolyQ (1:1000, Chemicon, IC2), mouse α-dmLaminB (1:500, DSHB), chicken α-MAP2 (1:1000, Abcam, ab5392), mouse α-γH2AX (1:1000, Millipore), rabbit α-TFEB (1:200, Bethyl Labs). A555,A488 or A633 conjugated goat α-mouse, α-rabbit or α-chicken secondary antibodies (1:500, Invitrogen) were used. To remove lipofuscin-like autofluorescence the stained sections were bleached with 10 nm $CuSO_4$ in ammonium acetate buffer (pH = 4.8) for 20 min [52] without affecting the specific antibody staining (Figure S2). To reveal the cell nuclei fluorescent dye Hoechst 33342 was applied in a concentration of 0.1 mg/ml in PBS for five min. Slices were mounted on microscopic slides in fluorescent mounting medium (DAKO). Confocal images of 40 to 90 cells were taken from 3 to 6 animals and analyzed morphometrically with experimenter being blinded to the genotype. Cells were imaged directly in multiwell plates in PBS and subjected to automated morphometric analysis.

Western blot analysis

In vivo brain lysates

Double transgenic mice (three per genotype and stage) were sacrificed by cervical dislocation followed by decapitation. The brains were dissected in ice cold PBS under a stereomicroscope. The two forebrain hemispheres were separated by medio-sagittal incision and detached form the diencephalon by an inclined cut to separate connecting fibers. The cerebellum was separated by disruption of cerebellar peduncles. The remaining parts comprising diencephalon, midbrain, pons, and formatio reticularis are referred to as the brainstem. The tissue was deep frozen in liquid nitrogen and stored at −80°C. The tissue was homogenized in modified RIPA buffer (137 mM NaCl, 20 mM Tris-HCl pH 7.5, 25 mM sodium glycerophosphate, 2 mM EDTA, 1 mM sodium-orthovanadate, 1%

(v/v) IGEPAL CA-630, 1% (w/v) deoxycholate, supplemented with Complete protease inhibitor cocktail (Roche)) using plastic pestles followed by three times repeated freezing in liquid nitrogen and thawing on ice. The supernatant was retrieved by 15 min centrifugation at full speed in Haereus Biofuge fresco (40474211, Kendro, rotor # 3328) cooled to 4°C. The pellet consisting of white top unconstrained fraction and transparent agglutinated bottom fraction was resuspended in homogenization buffer and homogenized by sonication (Brandelin Sonoplus UW2070).

Whole-cell lysates

Human fibroblast cultures were rinsed with PBS, lysed in modified RIPA buffer, scraped from the Petri dish incubated for 15 min on ice and centrifuged for 15 min at 4°C. Supernatants were subjected to analysis. The experiment was repeated three times on three independent days (n = 3). The lysates from the three independent experiments were loaded on one SDS-PAGE gel and densitometric evaluation of the western blot analysis was performed on the resulting triplicates.

Excretion analysis

Extracellular vesicle isolation via gradual ultracentrifugation and cytoplasmic and nuclear preparation were performed as described in [53]. Medium was removed from the cells and subjected to three centrifugation steps at 300 g, 10,000 g and 100,000 g at 4°C. The final pellet was washed in PBS and lysed in 1x Laemmli buffer. The results of from five independent experiments were subjected to statistical analysis.

Nuclear and cytoplasmic preparations

after aspiration of the medium the cells were washed in ice-cold PBS, detached with Trypsin-EDTA, washed in PBS and harvested in hypoosmotic homogenization buffer (10 mM HEPES, 10 mM KCl, 0.1 mM EDTA, 0.1 mM EGTA, 2 mM DTT, 25 mM NaF, 1 mM$NaVO_3$, PhosStop (Roche Applied Science), Complete protease inhibitor mixture (Roche Applied Science)). After incubation on ice for 15 min, the cells were lysed by the addition of 0.6% (v/v) Igepal CA-630 and vigorous vortexing. After centrifugation, the supernatant representing the cytoplasmic fraction was saved. The nuclear pellet was washed twice in homogenization buffer containing 0.6% (v/v) Igepal. The supernatant of the two washes was added to the cytoplasmic fraction. The nuclear pellet was dissolved by sonication in modified RIPA buffer. The results from five independent experiments subjected to western blot analysis were evaluated statistically.

SDS-PAGE and western blot assay

Protein concentration was determined using BCA assay kit (ThermoFischer Scientific) according to the manufacturer's protocol using a microplate reader at 495 nm. The protein lysates were denaturated in Laemmli buffer for 5 min at 95°C, followed by separation using SDS-PAGE by loading 100 μg of supernatant protein and 50 μg of pellet protein, respectively, and transferred onto nitrocellulose membrane (ECL, 0.2 μm pore size, Amersham) using BioRad Mini-Protean and Mini-Trans-Cell systems. Unspecific binding was blocked with 5% (w/v) low-fat milk powder or 5% (w/v) BSA in Tris-buffered saline supplemented with 0.1% (v/v) tween (TBST). The primary antibodies were incubated in blocking solution overnight at 4°C. Primary antibodies: mouse α-GFP (1:1000, Roche), rabbit α-LC3 (1:5000, MBL, PD014), mouse α-p62 (1:5000, Abnova, H00008878-M01), rabbit α-phospho-p70S6K (1:1000, Cell Signaling, 9205S), rabbit α-Atg5 (1:500, Novus Biological), rabbit α-Atg13-P-S318 (1:2000, Rockland) and mouse α–α-tubulin (1:20000, SIGMA, T9026). The HRP conjugated secondary α-mouse and α-rabbit antibodies (1:2500, Calbiochem). The α-LC3 antibody recognized the classic doublet for GFP-LC3 in the supernatant, but only the lower band, corresponding to LC3-II, was detected in the pellet of in-vivo tissue lysates (Figure S4B). The mouse α-GFP antibody only recognized this lower band in both supernatant and pellet of *in vivo* tissue lysates (Figure S4B). Secondary antibodies were incubated in 5% (w/v) low-fat milk powder in TBST for 1 h at room temperature. All specific signals were detected at the predicted molecular weight according to the molecular weight marker using SuperSignal West Pico Chemiluminescent Substrate (ThermoFisher Scientific). The densitometric analysis was performed using Image Studio Lite software (Li-Cor).

RNA extraction and quantitative RT-PCR

The cerebellum was dissected from wild-type, 26Q and 65Q mice (n = 6). The tissue was snap frozen in liquid nitrogen and stored at −80°C. The phenol/chlorophorm RNA extraction was performed using TRI Reagent (Invitrogen) according to the manufacturer's protocol. cDNA was generated using SuperScript III Reverse Transcriptase (Invitrogen). To quantify expression levels of *Tfeb*, cDNA template was amplified using UPL-based (UPL library, Roche) qPCR in combination with TaqMan Universal PCR Master mix on ABI 7900HT real-time PCR system (Applied Biosystems). For quantification (averages from triplicates) of expression levels of ER stress response and Tfeb target genes real-time qPCR was performed using the Light Cycler 480 system (Roche Applied Science, Indianapolis, IN, USA) as previously described [54]. All sequences used for the quantification are listed in the Key Resources Table.

QUANTIFICATION AND STATISTICAL ANALYSIS

Morphometric analysis

For Golgi size, H2AX, LaminB1, GFP-LC3, Lamp2a and GFP-RFP-LC3 puncta quantification was performed using ImageJ software. Confocal images of 40 to 90 cells from 3 to 6 animals were subjected to background subtraction, automatic thresholding (default and rely entropy, respectively) and particle analysis. For particle area, size, and number, 'analyze particles' command on binary images was applied. For intensity values gray level images were assessed after background subtraction. Colocalization analysis performed using images from the 'Green and Red Puncta Colocalization' macro [47]. Lipofuscin quantification was performed manually using the cell counter tool in ImageJ. The automated quantification of LaminB1 inclusions in the cytoplasm was performed using Columbus

software. The cellular regions were defined using functions such as find nuclei (Hoechst), find cytoplasm (LaminB1 background or EGFP) and find surrounding region for extracellular LaminB1 spots. Select population was applied to analyze whole cells only or to separate EGFP-positive from untransfected non-EGFP positive cells. The nuclear shape was analyzed using STAR shape analysis (area, roundness). Find spots function was employed for detection of LaminB1 or p62 specifically in the cytoplasm region as puncta per cell or, in cases of varied puncta size, area occupied by puncta per cell. The analysis was performed in at least 3 or 5 replicates including random selection of 20-40 imaging fields with multiple cells.

Statistical analysis

All quantifications were done manually in blind, unless otherwise stated. Statistical analysis was performed using Microsoft Excel and GraphPad Prism software. The data were tested for normality and equal variances using two-sided Z-test and F-test function in Excel as well as column statistics and Kolmogorov-Smirnov test in GraphPad prism. Subsequent comparison of differences between groups (genotypes, treatments) was assessed with adequate parametric and non-parametric tests for two group (Student's t test, Mann-Whittney) or multiple group comparisons (one-way ANOVA, Kruskal-Wallis). For comparison of two variables, e.g., genotype and treatment, at a time (variable1 = v1, variable2 = v2) two-way ANOVA was employed.

For animal experiments power calculations were not performed due to the exploratory nature of this study. Behavioral data were tested for significant differences using repeated-measures two-way ANOVA, thereby separating males ($n \geq 8$) and females ($n \geq 8$), since the values of control animals varied significantly between sexes. No significant differences were detected between the animals from the two rounds behavioral analysis, nor animals expressing GFP-LC3 in addition to ATN1 transgene. In the histological and qPCR analysis no differences between males (n = 3) and females (n = 3) were evident. Therefore statistical significances were analyzed between mixed gender groups using one-way ANOVA, unless otherwise stated. For western blot assays the densitometric data (males, n = 3) was analyzed using unpaired Student's t test, unless otherwise stated.

Current Biology, Volume *27*

Supplemental Information

Stall in Canonical Autophagy-Lysosome Pathways Prompts Nucleophagy-Based Nuclear Breakdown in Neurodegeneration

Olga Baron, Adel Boudi, Catarina Dias, Michael Schilling, Anna Nölle, Gema Vizcay-Barrena, Ivan Rattray, Heinz Jungbluth, Wiep Scheper, Roland A. Fleck, Gillian P. Bates, and Manolis Fanto

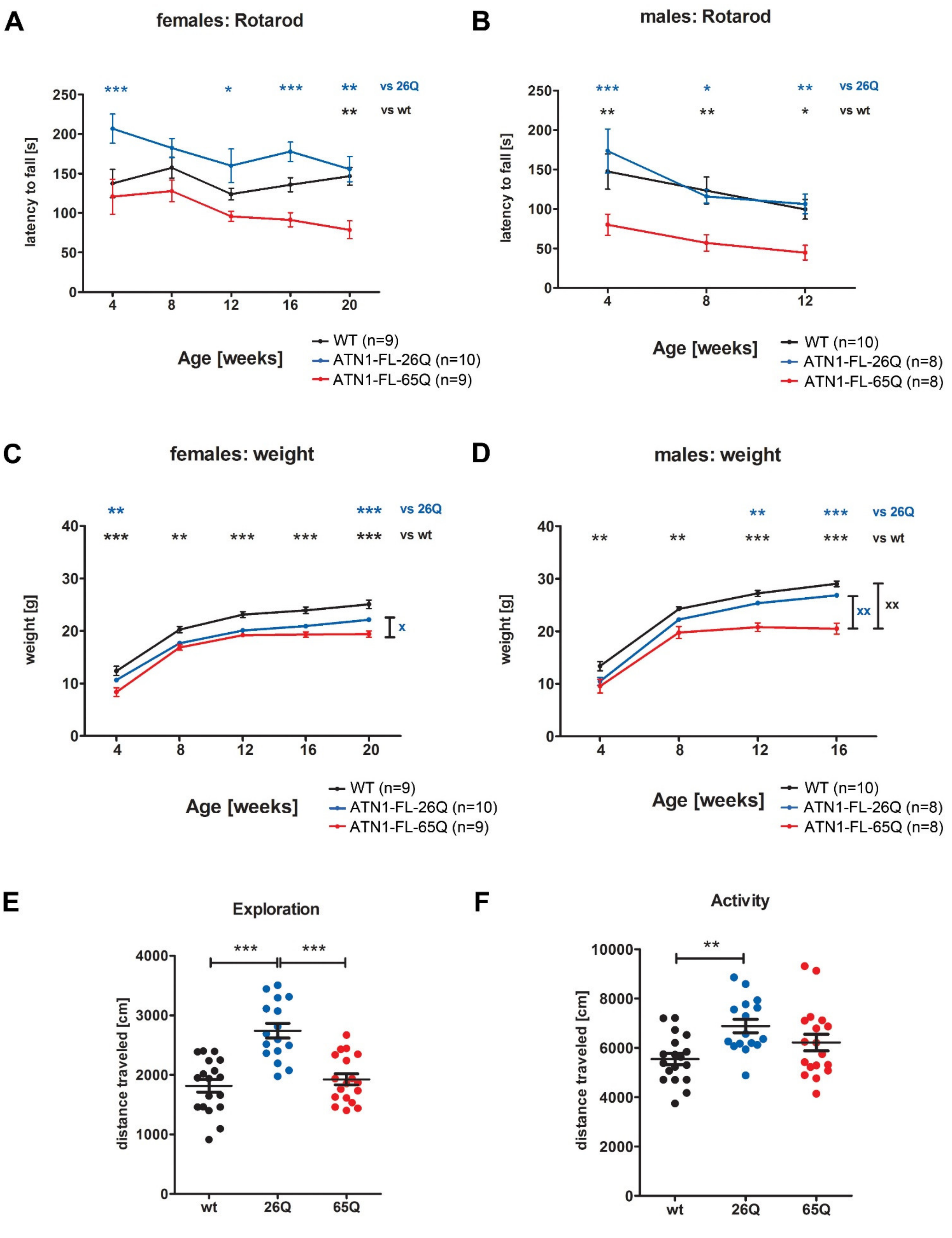

Figure S1. Related to Figure 1 – Behavioral assessment of DRPLA mice

(A – B) Rotarod performance was assed every four weeks starting at four weeks of age. Average values for two experimental days with three trial each were analysed per animal (n) at the given time point. Statistical analysis using repeated measures two-way ANOVA did not show any interaction between genotype (v2) over time (v1) assigning no progressive decrease of rotarod performance. The individual values for females (A) and males (B) at each time point are given as mean ± SEM, showing significantly decreased rotarod performance for the ATN1-FL-65Q line (red) compared to ATN1-FL-26Q (blue) and wild type (WT, black): *$p<0.05$, **$p<0.01$, ***$p<0.001$; comparison ATN1-FL-65Q versus wild type (vs wt, black), and ATN1-FL-65Q vs ATN1-FL-26Q (vs 26Q, blue).

(C – D) Weight was assessed every four weeks starting at four weeks of age. Females (C) ATN1-FL-65Q mice (black) showed progressive lack of weight gain only compared to ATN1-FL-26Q (26Q; blue). Male (D) ATN1-FL-65Q mice (black) showed a progressive lack of weight gain compared to ATN1-FL-26Q (26Q; blue) line and wild type mice (red). Repeated measures two-way ANOVA: interaction between genotypes (v2) with age (v1): $^{X}p<0.05$, $^{XX}p<0.01$. The individual values for females (C) and males (D) at each time point are given as mean ± SEM. **$p<0.01$, ***$p<0.001$; comparison ATN1-FL-65Q versus wild type (vs wt, black), and ATN1-FL-65Q vs ATN1-FL-26Q (vs 26Q, blue).

(E) Explorative behaviour was assessed in males and females at the age 10 weeks by measuring the activity for the first 5 min after introduction to the open field. ATN1-FL-26Q (26Q; blue) was significantly more active compared to wild type mice (wt; black) and the ATN1-FL-65Q line (65Q; red). The ATN1-FL-65Q mice showed no significant differences compared to their wt littermates. Mean ± SEM, One-way ANOVA, ***$p<0.001$.

(F) General activity for females and males showing the total distance travelled over the interval from 5 to 25 min after introduction to the open field. ATN1-FL-26Q (26Q; blue) was significantly more active compared to wild type mice (wt; black) and unchanged compared to ATN1-FL-65Q (65Q, red) at 10 weeks. Mean ± SEM, one way ANOVA, **$p<0.01$.

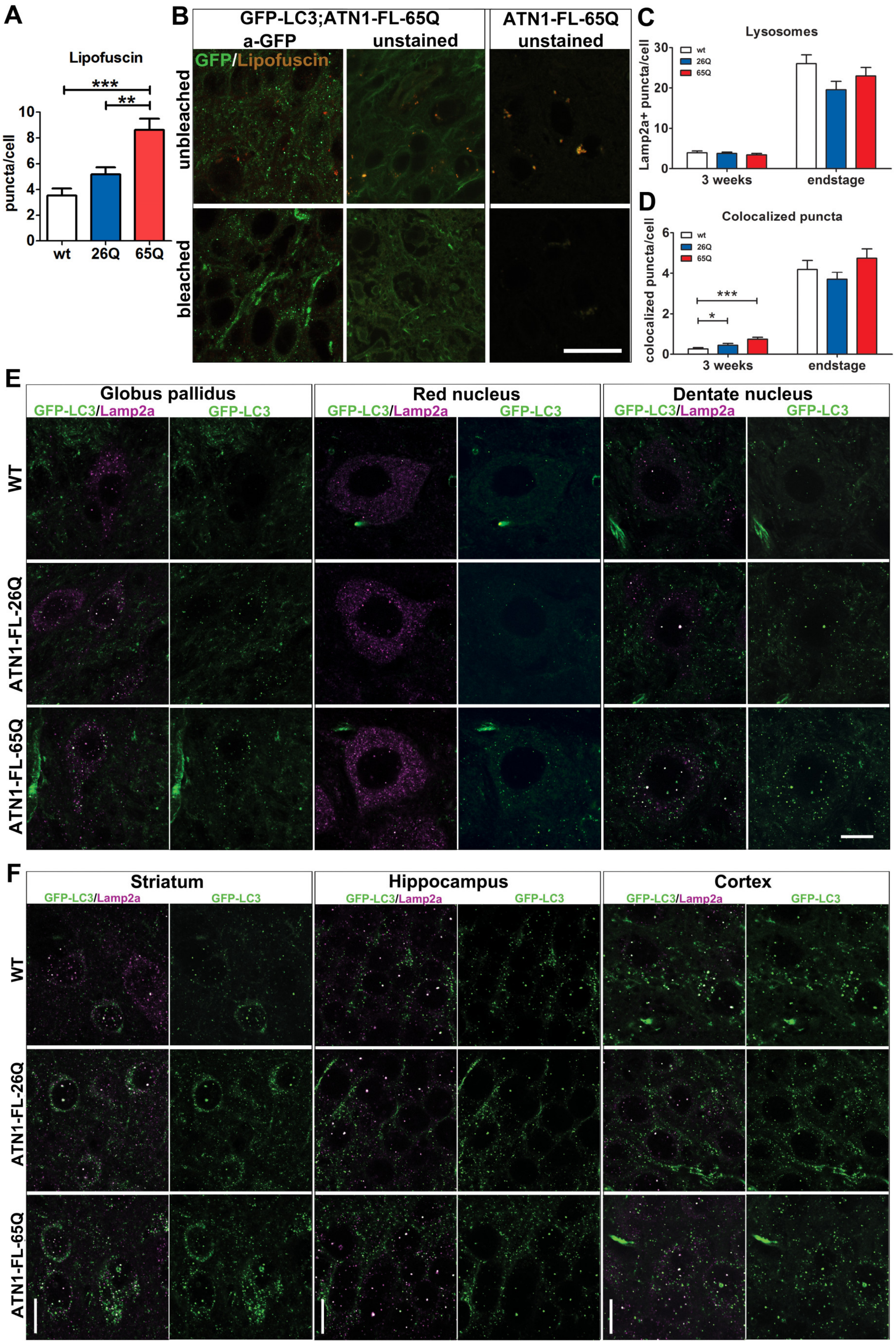

A
Lipofuscin
***
**
puncta/cell
0
2
4
6
8
10
wt
26Q
65Q
B
GFP-LC3;ATN1-FL-65Q
a-GFP
unstained
ATN1-FL-65Q
unstained
GFP/Lipofuscin
unbleached
bleached
C
Lysosomes
Lamp2a+ puncta/cell
0
10
20
30
wt
26Q
65Q
3 weeks
endstage
D
Colocalized puncta
colocalized puncta/cell
0
2
4
6
***
*
3 weeks
endstage
E
Globus pallidus
Red nucleus
Dentate nucleus
GFP-LC3/Lamp2a
GFP-LC3
WT
ATN1-FL-26Q
ATN1-FL-65Q
F
Striatum
Hippocampus
Cortex
GFP-LC3/Lamp2a
GFP-LC3
WT
ATN1-FL-26Q
ATN1-FL-65Q

Figure S2. Related to Figure2 – Anatomical representation of autophagic markers in the brains of DRPLA mice.

(A) Quantification of autofluorescent lipofuscin accumulation in DN neurons in endstage mice. ATN1-FL-65Q mice displayed a significant increase of lipofuscin dots in comparison to ATN1-FL-26Q and WT controls. Mean ± SEM, One-way ANOVA, ***$p<0.001$, **$p<0.01$.

(B) Bleaching efficiency of lipofuscin autofluorescence shown on the example of cortical slices form the ATN1-FL-65Q line. Confocal images of lipofuscin-like fluorescence shown in two different channels (red+green=orange) before (top panel) and after (bottom panel) bleaching in non-GFP ATN1-FL-65Q line (right); as well as ATN1-FL-65Q;GFP-LC3 double mutant line in unstained (middle) with native GFP (green) signal and after the staining with mouse α-GFP antibody (left). Scale bar 10 µm.

(C) Statistical analysis of LAMP2a positive puncta in dentate nucleus cells of wt;GFP-LC3 (wt), ATN1-FL-26Q;GFP-LC3 (26Q) and ATN1-FL-65Q;GFP-LC3 (65Q) mice at the presymptomatic stage of 3 weeks (wt, (n=83 cells,3 animals), 26Q (n=77 cells,3 animals), 65Q (n=96 cells,4 animals) and endstage (wt (n=53 cells,3 animals), 26Q (n=49 cells,3 animals), 65Q (n=52 cells, 3 animals). One-way ANOVA, mean ± SEM, not significant.

(D) Statistical analysis of GFP-LC3 positive puncta co-localised with LAMP2a positive puncta in dentate nucleus cells of wt;GFP-LC3 (wt), ATN1-FL-26Q;GFP-LC3 (26Q) and ATN1-FL-65Q;GFP-LC3 (65Q) mice at the presymptomatic stage of 3 weeks (wt (n=83 cells,3 animals), 26Q (n=77 cells,3 animals), 65Q (n=96 cells,4 animals) and endstage (wt (n=53 cells,3 animals), 26Q (n=49 cells,3 animals), 65Q (n=52 cells, 3 animals). One-way ANOVA, mean ± SEM, *$p<0.05$, ***$p<0.001$.

(E) Representative images of the three main regions that degenerate in DRPLA patients – globus pallidus (left), red nucleus (middle) and dentate nucleus (right) - from wt;GFP-LC3 (WT, top), ATN1-FL-26Q;GFP-LC3 (middle) and ATN1-FL-65Q;GFP-LC3 (bottom) endstage mice evaluated for GFP-LC3 (green) and LAMP2a (magenta) positive puncta as well as co-localised (white) puncta. Images were taken with the confocal laser scanning microscope. Scale 5 µm.

(F) Representative images of 3 forebrain regions – striatum (left), dentate gyrus of the hippocampus (middle) and cortex (right) - from wt;GFP-LC3 (top), ATN1-FL-26Q;GFP-LC3 (middle) and ATN1-FL-65Q;GFP-LC3 (bottom) endstage mice evaluated for GFP-LC3 (green) and LAMP2A (magenta) positive puncta as well as co-localised (white) puncta. Images were taken with the confocal laser scanning microscope. Scale 5 μm.

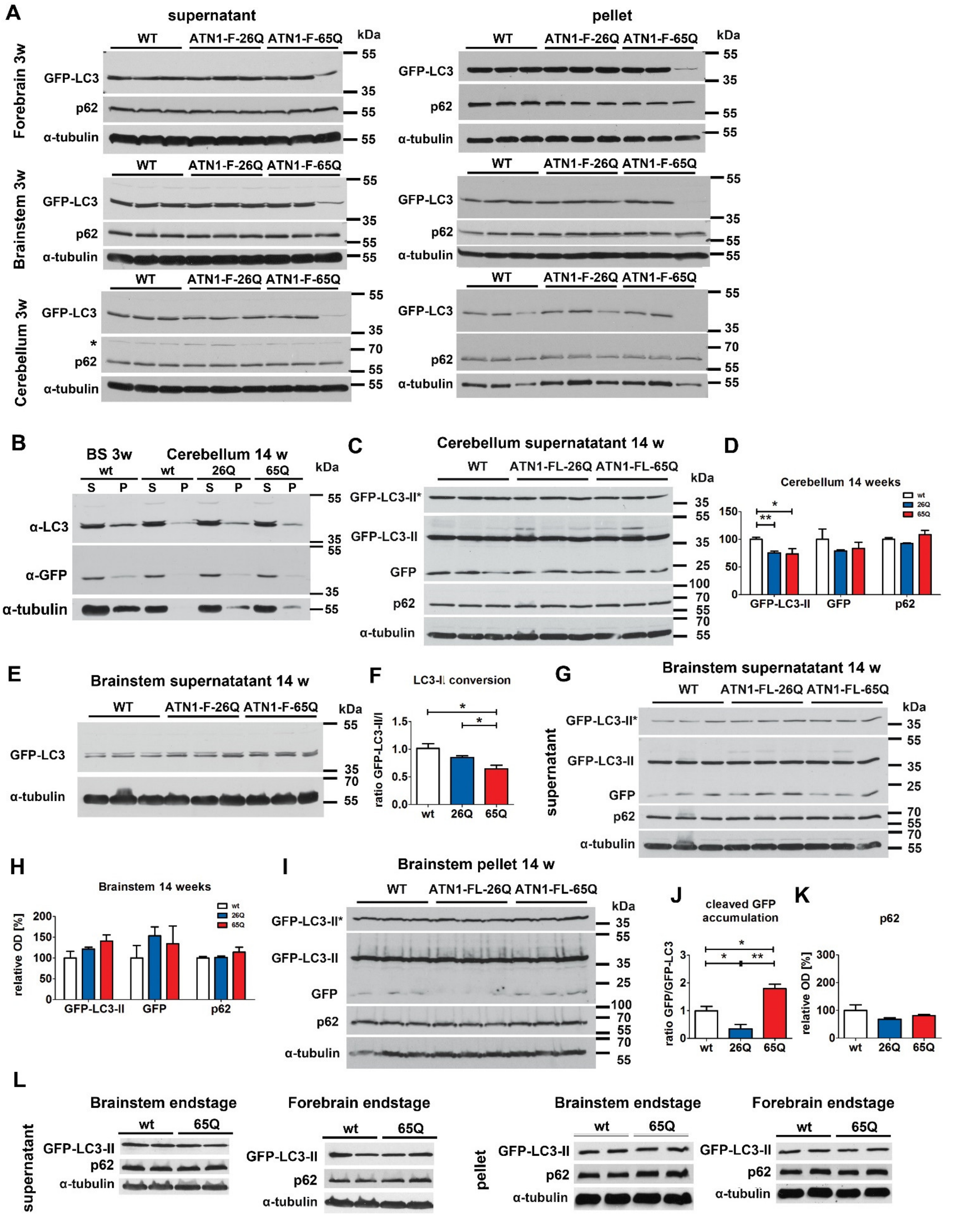

A
supernatant
pellet
Forebrain 3w
Brainstem 3w
Cerebellum 3w
WT
ATN1-F-26Q
ATN1-F-65Q
kDa
GFP-LC3
p62
α-tubulin
B
BS 3w
Cerebellum 14 w
wt
26Q
65Q
S
P
α-LC3
α-GFP
C
Cerebellum supernatatant 14 w
ATN1-FL-26Q
ATN1-FL-65Q
GFP-LC3-II*
GFP-LC3-II
GFP
D
Cerebellum 14 weeks
E
Brainstem supernatatant 14 w
F
LC3-II conversion
ratio GFP-LC3-II/I
G
Brainstem supernatatant 14 w
supernatant
H
Brainstem 14 weeks
relative OD [%]
I
Brainstem pellet 14 w
J
cleaved GFP accumulation
ratio GFP/GFP-LC3
K
L
Brainstem endstage
Forebrain endstage
pellet

Figure S3. Related to Figure 3 – Autophagy flux analysis in DRPLA mice and patient fibroblasts.

(A) No differences in GFP-LC3 (α-LC3 antibody), and p62 were observed in supernatant (left panel) or pellet (right panel) fractions of forebrain (top), brainstem (middle) and cerebellum (bottom) lysates from three weeks old wt;GFP-LC3, ATN1-FL-26Q;GFP-LC3 and ATN1-FL-65Q;GFP-LC3 mice, except for one outlier sample for ATN1-FL-65Q (last lane), which behaved differently as compared to the eight sister samples. α-tubulin was used as a loading control. * indicates an additional p62 band running at about ~85 kDa besides the expected band at ~62 kDa in the supernatant fraction of the cerebellum only. Molecular weight in kDa (left).

(B) Western blot analysis of GFP-LC3 in supernatant (S) and pellet (P) fractions of the wt;GFP-LC3 brainstem (BS) at three weeks (3w) as well as cerebellum at 14 weeks from wt;GFP-LC3 (wt), ATN1-FL-;GFP-LC3 (26Q) and ATN1-FL-65Q;GFP-LC3 (65Q) mice. α-tubulin was used as a loading control showing a lower abundance in the pellet fractions. The anti-LC3 antibody recognised a GFP-LC3 doublet (GFP-LC3-I corresponds to the upper band, GFP-LC3-II to the lower band) in the supernatant fraction, while only one band appeared in the pellet fraction corresponding to the lower GFP-LC3-II band. The mouse anti GFP antibody recognised only the lower band GFP-LC3-II in both supernatant and pellet fraction.

(C – D) Western blot analysis of GFP-LC3-II, cleaved GFP and p62 in the supernatant fraction of the cerebellar lysates at 14 weeks. Anti-GFP antibody recognises GFP-LC3II and cleaved GFP after longer exposure. *- shorter exposure of anti-GFP-LC3 signal (D). Densitometric analysis of relative abundance of GFP-LC3-II, cleaved GFP and p62 normalised to α-tubulin in supernatant of cerebellum in wt;GFP-LC3 mice (wt), ATN1-FL-26Q;GFP-LC3 (26Q) and ATN1-FL-65Q;GFP-LC3 (65Q) mice. Student's t-test, mean ± SEM, *$p<0.05$, **$p<0.01$ (E).

(E - F) Western blot analysis of full-length GFP-LC3 in the supernatant fraction of the brainstem lysates at 14w of age (E). Densitometric analysis shows decreased relative abundance of cleaved GFP-LC3-II to full-length GFP-LC3-I (F) in ATN1-FL-65Q;GFP-LC3 (65Q) compared to wt;GFP-LC3 (wt) and ATN1-FL-26Q;GFP-LC3 (26Q) mice. Student's t-test, mean ± SEM, *$p<0.05$.

(G – H) Western blot analysis of GFP-LC3II, cleaved GFP and p62 in the supernatant fraction of the brainstem lysates at 14 weeks of age. Anti-GFP antibody recognises GFP-LC3-II and cleaved

GFP after longer exposure. *- shorter exposure of anti-GFP-LC3 signal (G). Densitometric analysis of the relative abundance of GFP-LC3-II, cleaved GFP and p62 relative to α-tubulin in supernatant of brainstem in wt;GFP-LC3 (wt), ATN1-FL-26Q;GFP-LC3 (26Q) and ATN1-FL-65Q;GFP-LC3 (65Q) mice. Student's t-test, mean ± SEM, *p<0.05, **p<0.01 (H).

(I – K) Western blot analysis of GFP-LC3-II, cleaved GFP and p62 in the pellet fraction of the brainstem lysates at 14w (I). Densitometric analysis of relative abundance of cleaved GFP to GFP-LC3-II (J) revealed an increase in ATN1-FL-65Q;GFP-LC3 (65Q) mice compared to wt;GFP-LC3 (wt) and ATN1-FL-26Q;GFP-LC3 The abundance of p62 relative to α-tubulin was unchanged (K). Student's t-test, mean ± SEM, *p<0.05, **p<0.01.

(L) Western blot analysis of GFP-LC3-II and p62 in the supernatant (top) or pellet (bottom) fractions of brainstem (left) and forebrain (right) lysates from endstage wt;GFP-LC3 (wt) and. α-tubulin was used as a loading control.

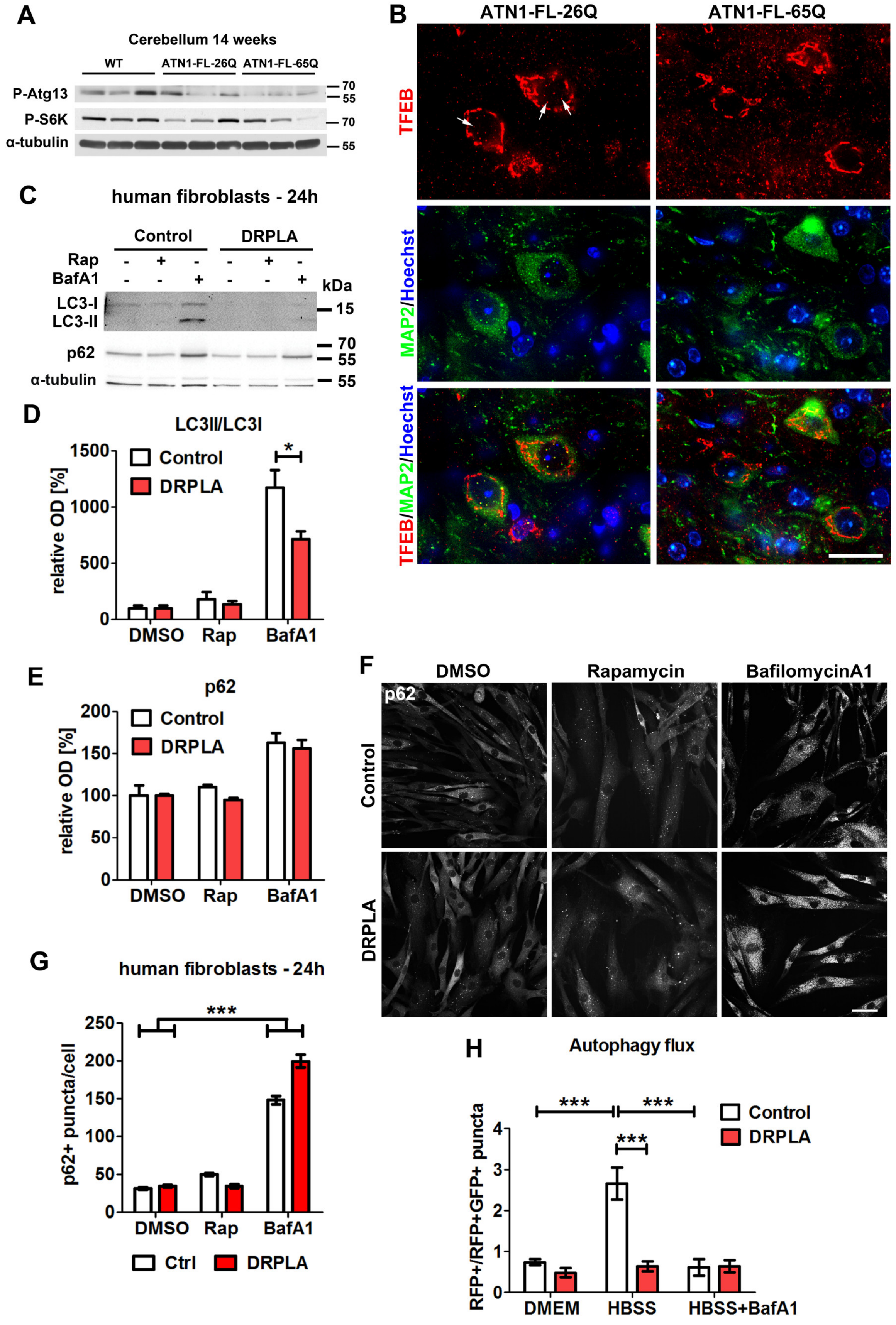
A
Cerebellum 14 weeks
WT
ATN1-FL-26Q
ATN1-FL-65Q
P-Atg13
P-S6K
α-tubulin
70
55
70
55
B
ATN1-FL-26Q
ATN1-FL-65Q
TFEB
MAP2/Hoechst
TFEB/MAP2/Hoechst
C
human fibroblasts - 24h
Control
DRPLA
Rap
BafA1
kDa
LC3-I
LC3-II
p62
α-tubulin
15
70
55
55
D
LC3II/LC3I
Control
DRPLA
relative OD [%]
1500
1000
500
0
DMSO
Rap
BafA1
E
p62
Control
DRPLA
relative OD [%]
200
150
100
50
0
DMSO
Rap
BafA1
F
DMSO
Rapamycin
BafilomycinA1
p62
Control
DRPLA
G
human fibroblasts - 24h
p62+ puncta/cell
250
200
150
100
50
0
DMSO
Rap
BafA1
Ctrl
DRPLA
H
Autophagy flux
Control
DRPLA
RFP+/RFP+GFP+ puncta
4
3
2
1
0
DMEM
HBSS
HBSS+BafA1

Figure S4. Related to Figure 3. – Autophagy flux analysis in DRPLA patients' fibroblasts

(A) Western blot analysis of the phosphorylation status of Atg13 at S318 and p70S6K (phospho-S6K), a target of mTOR kinase in the supernatant fraction of the cerebellar lysates from 14 weeks old wt;GFP-LC3, ATN1-FL-26Q;GFP-LC3 and ATN1-FL-65Q;GFP-LC3 mice. The ATN1-FL-65Q;GFP-LC3 mice show a reduction compared to wt;GFP-LC3. α-tubulin was used as a loading control. Molecular weight in kDa (left).

(B) Representative images of Tfeb staining in DN neurons from endstage mice. MAP2 marks specifically neuronal cells. Tfeb is present in discrete puncta inside the nucleus in ATN1-FL-26Q;GFP-LC3 in contrast to atrophic Map2-positive DN cells in ATN1-FL-65Q;GFP-LC3 mice. Scale bar 20 μm.

(C – E) Supernatants from human fibroblasts from DRPLA (17) patients and age matched healthy control were subjected to western blot analysis for endogenous LC3I and LC3II as well as p62. Block of autophagy with BafA1 for 24h resulted in increase of LC3II as well as p62 compared to DMSO control conditions in control fibroblasts. Densitometry reveals that DRPLA patient samples showed a mild response with a decrease significant decrease in LC3I to II conversion when treated with BafA1 (D). Student's t-test, mean ± SEM, *$p<0.05$, **$p<0.01$.

(F – G) Analysis of p62 puncta in the cytoplasm in DRPLA (17) patient fibroblasts and age matched control after 24 hrs treatment with Rap and BafA1. Representative images show cells after 24 hrs treatment stained with anti-p62 antibody. Scale bar 50 μm. (F). Upon BafA1 treatment both control and DRPLA fibroblasts show an increase in p62 positive puncta, hereby significantly more in DRPLA cells ($p<0.001$). Automated quantification of nuber of p62-positive puncta in the cytoplasm was performed using Opera Phenix high content screening system and Columbus software. Mean ± SEM, two way-ANOVA ***$p<0.001$, v1 – genotype, v2 – treatment.(G).

(H) Ratio of autolysosomes (RFP+) to autophagosomes (GFP+RFP+) as a measure of autophagy flux progression in control and DRPLA fibroblasts transfected with the tandem RFP-GFP-LC3B reporter (shown in Fig 3. M,N). Acute starvation of control cells in HBSS for 2 hours induces a significantly greater transition towards autolysosomes, which is blocked by BafA1 treatment. DRPLA fibroblasts do not display similar formation and maturation of autophagosomes reflected

in significant difference from controls in HBSS. Two-way ANOVA, v1 – genotype, v2 – treatment, mean ± SEM, ***$p<0.001$.

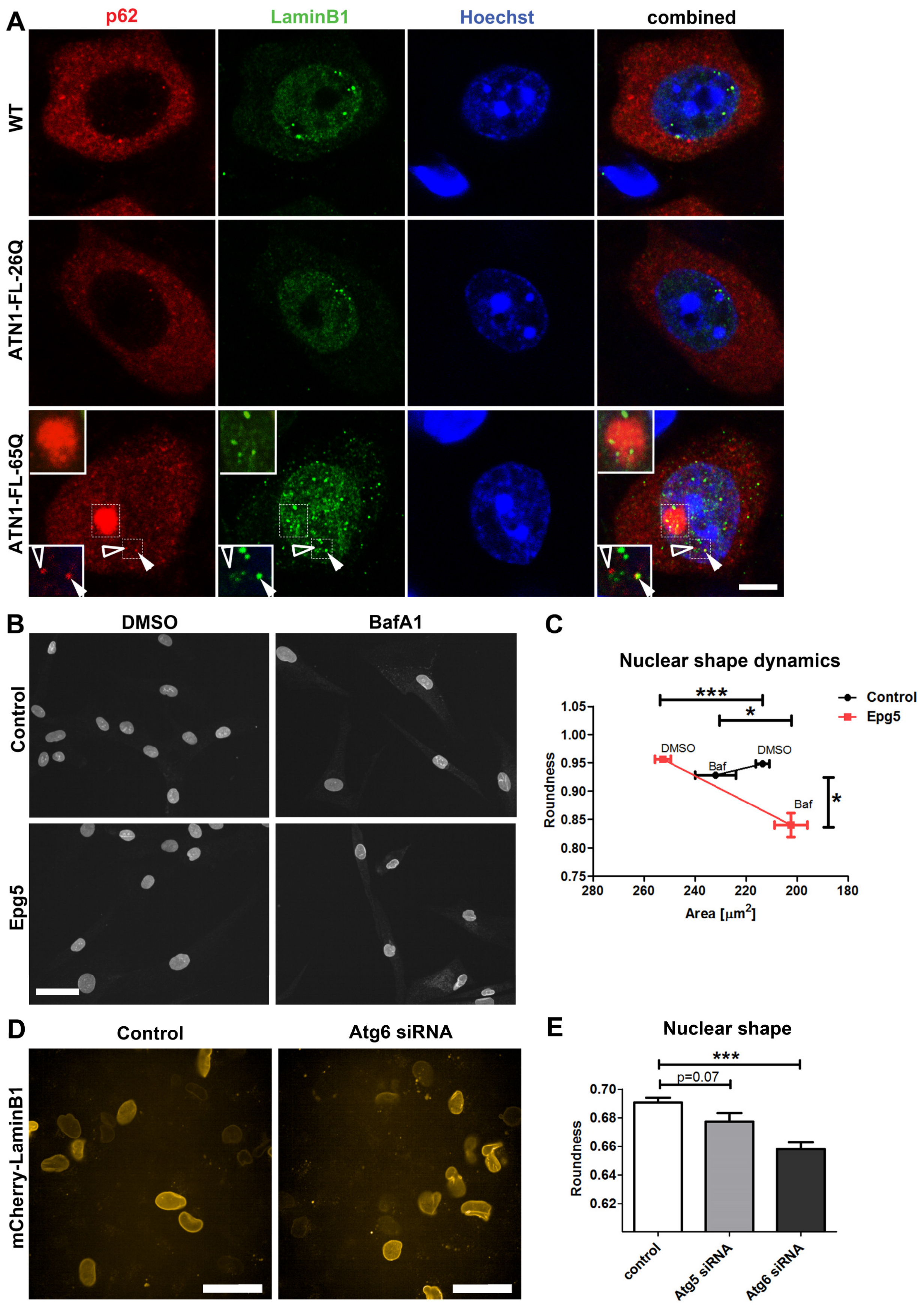
A
p62
LaminB1
Hoechst
combined
WT
ATN1-FL-26Q
ATN1-FL-65Q
B
DMSO
BafA1
Control
Epg5
C
Nuclear shape dynamics
***
*
Control
Epg5
DMSO
Baf
Roundness
Area [μm2]
D
Control
Atg6 siRNA
mCherry-LaminB1
E
Nuclear shape
p=0.07
Roundness
control
Atg5 siRNA
Atg6 siRNA

Figure S5. Related to Figure 4 – Nuclear shape disruption upon defective autophagy flux.

(A) Confocal fluorescence microscopy images of the dentate nucleus from endstage wild type (WT), ATN1-FL-26Q and ATN1-FL-65Q mice. Diffuse nuclear Lamin-B1 staining (green) is observed along with speckled staining which is more pronounced in ATN1-FL-65Q mice in the nucleus as well as in the cytoplasm. Lamin-B1 is in close proximity of (framed arrowhead), or co-localises with, p62 (red) puncta (full arrowhead) and the p62 inclusion (inserts). Scale bar 5 µm.

(B – C) Analysis of nuclear shape dynamics in fibroblasts from Vici patient with mutation in autophagy gene EPG5 (p.Phe1604Glyfs*20) and age matched healthy individual. Representative images show nuclei revealed by the LaminB1 antibody upon treatment with BafA1 or DMSO as a control for 48 hrs (B). BafA1 treatment resulted in structural changes in cells carrying mutation in Epg5 gene, reflected by a significant decrease in nuclear size and roundness (D). The plot of roundness versus area shows an opposite trend and convergence of control and Epg5 defective nuclei upon BafA1 treatment (C). Automated quantification was performed using Opera Phenix high content screening system and Columbus software. Mean ± SEM, n=5, two-way-ANOVA ***p<0.001, * p<0.05, v1 – genotype, v2 – treatment. Scale bar 50 µm.

(D – E) Representative images of nuclei in SK-N-BE(2) human neuroblastoma cells expressing mCherry-LaminB1 and Atg6 siRNA or non-specific control siRNA (A). Quantification of nuclear roundness marked by mCherry-LaminB1 reveals a significantly increased irregularity of the nuclear shape in cells overexpressing Atg6 siRNA (B). Student's t-test, mean ± SEM, ***p<0.001. Scale bar 20 µm.

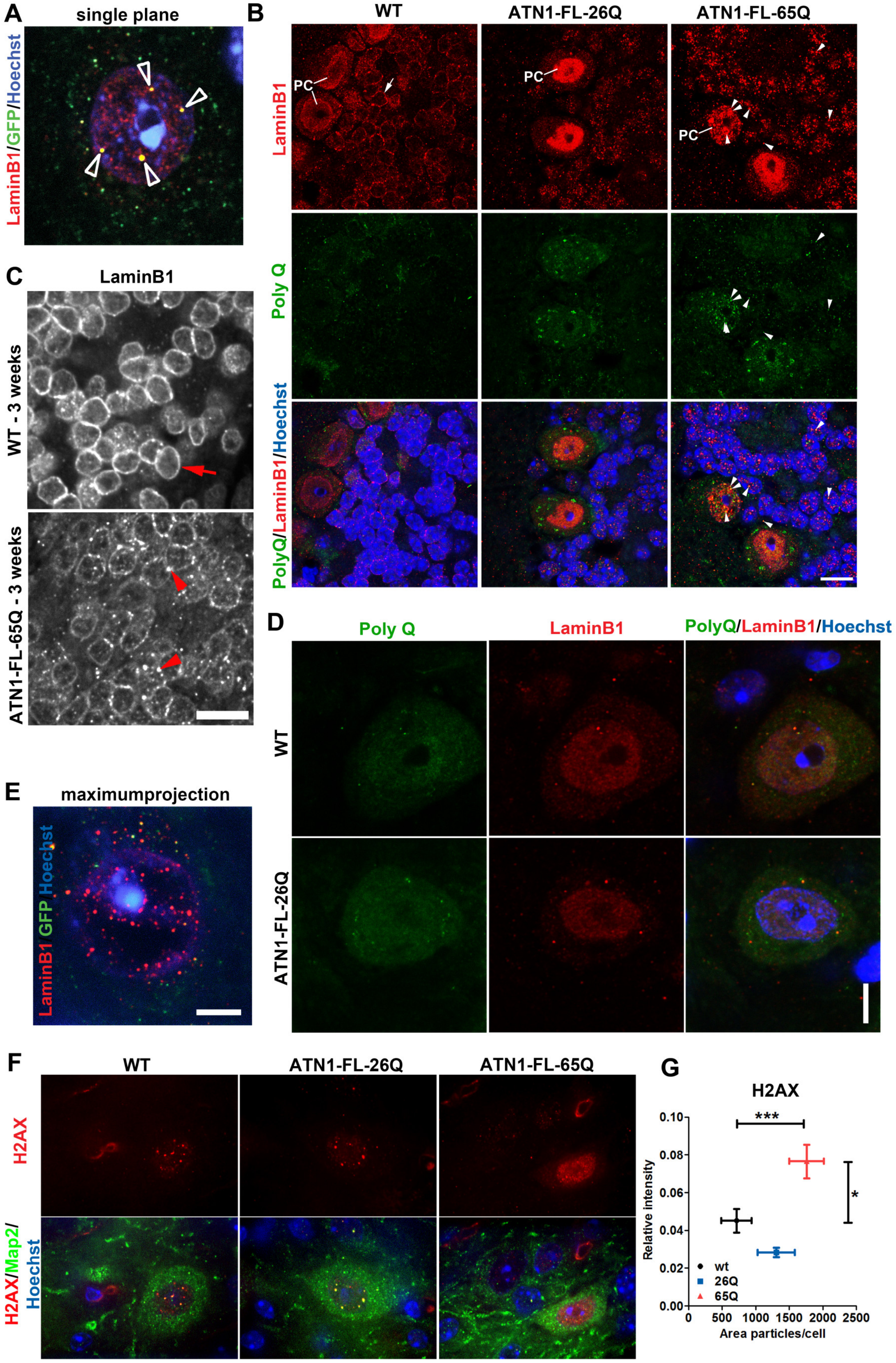

A
single plane
LaminB1/GFP/Hoechst
B
WT
ATN1-FL-26Q
ATN1-FL-65Q
LaminB1
PC
Poly Q
PolyQ/LaminB1/Hoechst
C
LaminB1
WT - 3 weeks
ATN1-FL-65Q - 3 weeks
D
Poly Q
LaminB1
PolyQ/LaminB1/Hoechst
WT
ATN1-FL-26Q
E
maximumprojection
LaminB1 GFP Hoechst
F
WT
ATN1-FL-26Q
ATN1-FL-65Q
H2AX
H2AX/Map2/Hoechst
G
H2AX
Relative intensity
Area particles/cell
***
*
wt
26Q
65Q
0.10
0.08
0.06
0.04
0.02
0.00
0
500
1000
1500
2000
2500

Figure S6. Related to Figure 5 – Nuclear LaminB1 pathology, LaminB1 association with native PolyQ and terminal stage for nuclei in DRPLA mice.

(A) LaminB1 (red) shows a remarkable co-localisation (arrow heads) with GFP-LC3 (green) inside the nucleus (blue) of ATN1-FL-65Q;GFP-LC3 Dentate Nucleus cells. Scale bar 5 μm.

(B) LaminB1 shows a classical perinuclear ring structure (arrow) representing the nuclear lamina cortex in the cerebellar granule cells in wild type (WT) mice, while the Purkinje cells (PC) show, in addition to the lamina ring, a diffuse intranuclear matrix staining. ATN1-FL-26Q mice show reorganization of LaminB1 into speckled matrix in the granule cell nuclei and mostly diffused intranuclear staining in PC. This is more pronounced in the cerebellar cortex of ATN1-FL-65Q mice, in which large LaminB1 speckles form in both cell types colocalise with PolyQ inclusions (arrowheads) in particular in PC. Scale bar 5 μm.

(C) At 3 weeks LaminB1 shows a classical perinuclear ring structure (red arrow) in granule cells of ATN1-FL-65Q mice similarly as in wild type (WT), albeit there is tendency to forming dense LaminB1 punctae in the periphery of the nuclear cortex in ATN1-FL-65Q granule cells (red arrowhead). Scale bar 10 μm.

(D) The α-PolyQ IC2 antibody immunized against polyQ fragment of human TBP also recognises native PolyQ in wild type (WT) and ATN1-FL-26Q mice as distinct focal puncta in the nuclear periphery and cytoplasm. Scale bar 5 μm.

(E) Some of the dentate nucleus cells in ATN1-FL-65Q;GFP-LC3 endstage mice show a severely disrupted nuclear morphology with weak and partially disrupted nuclear staining (blue,), devoid of LaminB1. Maximum projection from confocal z-Stack. Scale bar 5 μm.

(F-G) Analysis of γH2AX levels in DN neurons of endstage mice. Map2 is used to mark neuronal cells. In representative images (F) atrophic cells of ATN1-FL-65Q;GFP-LC3 endstage mice γH2AX is strongly upregulated throughout the nucleus of Map2-positive neurons (green). Scale bar 10 μm. Quantification (G) displays a significant increase in both the area occupied by γH2AX foci and their intensity in ATN1-FL-65Q;GFP-LC3 mice compared to ATN1-FL-26Q;GFP-LC3 and wt;GFP-LC3 controls. The most degenerated DN cells, with hollow nucleus (as in panel B) were not included in the quantification. Kruskal-Wallis multiple comparison analysis, mean ± SEM, ***$p<0.001$, *$p<0.05$.

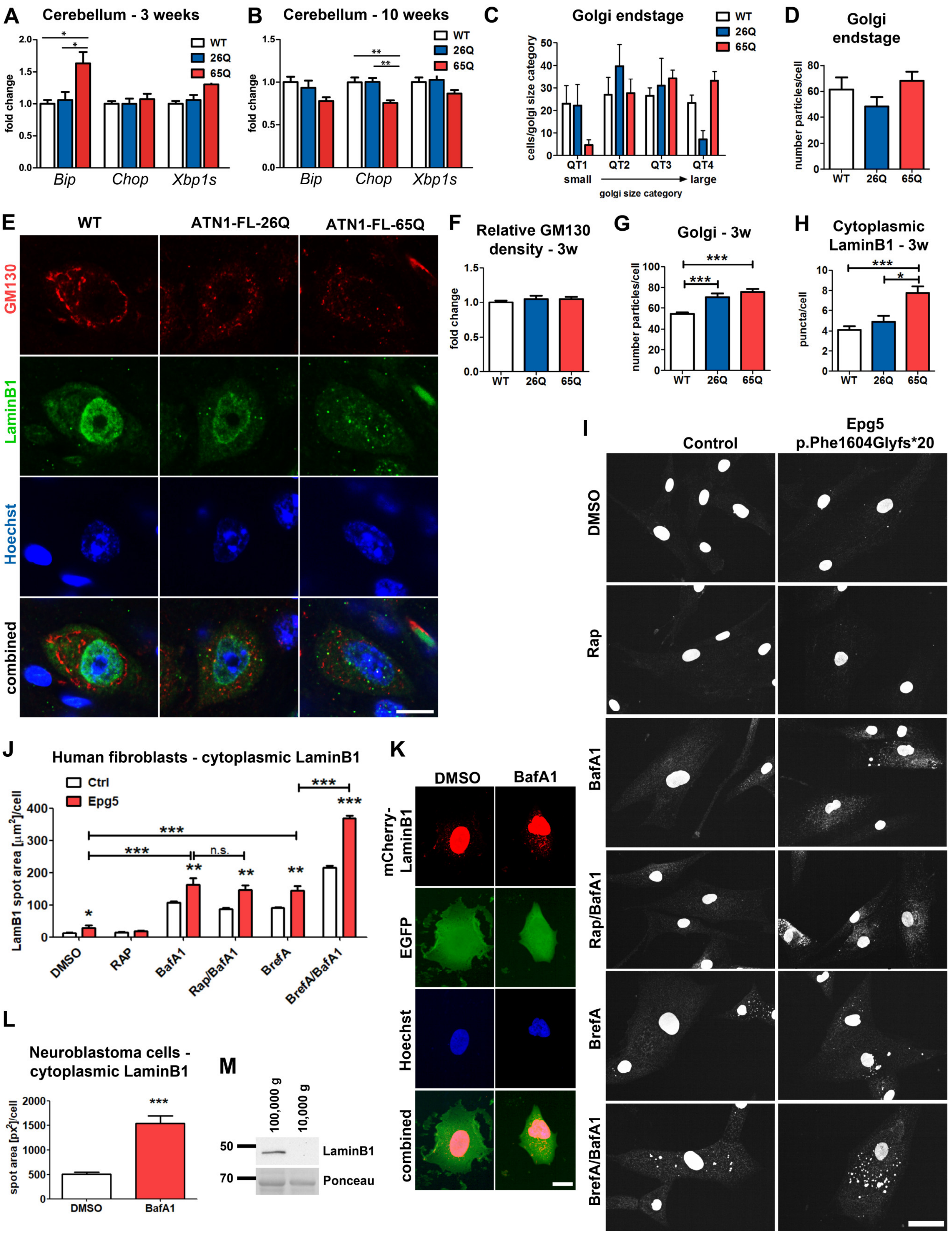

A
Cerebellum - 3 weeks
WT
26Q
65Q
fold change
Bip
Chop
Xbp1s
B
Cerebellum - 10 weeks
C
Golgi endstage
cells/golgi size category
QT1
QT2
QT3
QT4
small
large
golgi size category
D
Golgi endstage
number particles/cell
E
WT
ATN1-FL-26Q
ATN1-FL-65Q
GM130
LaminB1
Hoechst
combined
F
Relative GM130 density - 3w
G
Golgi - 3w
H
Cytoplasmic LaminB1 - 3w
puncta/cell
I
Control
Epg5 p.Phe1604Glyfs*20
DMSO
Rap
BafA1
Rap/BafA1
BrefA
BrefA/BafA1
J
Human fibroblasts - cytoplasmic LaminB1
Ctrl
Epg5
LamB1 spot area [µm²]/cell
n.s.
DMSO
RAP
BafA1
Rap/BafA1
BrefA
BrefA/BafA1
K
DMSO
BafA1
mCherry-LaminB1
EGFP
Hoechst
combined
L
Neuroblastoma cells - cytoplasmic LaminB1
spot area [px²]/cell
DMSO
BafA1
M
100,000 g
10,000 g
50
70
LaminB1
Ponceau

Figure S7. Related to Figure 6 and 7. – Transient ER stress activation correlates with differentially progressing structural Golgi alterations in DRPLA mice. Golgi dependent LaminB1 degradation.

(A – B) ER stress activation was assessed in the cerebellum of 3 weeks (A) and 10 weeks (B) old ATN1-FL-65Q mouse (65Q, red), ATN1-FL-26Q (26Q, blue) and wild type (wt; white) mice. The levels of the UPR target mRNAs Bip, Chop and Xbp1s were determined by qPCR. *Hprt1* was used as reference gene. Values were normalised to wt and statistically analysed by one-way ANOVA (*$p<0.05$ and **$p<0.01$). Graph represents mean values ± SEM (n = 6 animals).

(C – D) Quantification of Golgi size in endstage mice according to average size of GM130-positive structures in the cells distributed in 4 categories QT1 to QT4 (small to large) defined according to values at 25‰ intervals in WT mice). ATN1-FL-65Q mice show more cells in the DN which have on average larger GM130-positive structures, while ATN1-FL-26Q show a shift towards on average smaller GM130-positive structures (C). On average, the number of GM130 positive structures was unchanged. Kruskal-Wallis multiple comparison anlalysis, Mean ± SEM, not significant.

(E-H) Representative spinning disc confocal images of GM130-positive Golgi structures and LaminB1 immunoreactivity in the DN cells of 3 weeks old animals. Scale bar 10 µm (E). Quantification of GM130-positive structures showed no significant changes with respect to the total area occupied by GM130-positive staining in the cells between genotypes (F). The significantly increased number of GM130-positive particles (G) correlates well with decreased average size of GM130-positive structures (data not shown) in ATN1-FL-26Q and ATN1-FL-65Q mice, indicating Golgi fragmentation at an early presymptomatic stage of 3 weeks. DN cells ATN1-FL-65Q mice show an increased number of LaminB1 positive puncta, some colocalising with GM130, in the cytoplasm already at presymptomatic stage of 3 weeks compared to wild type and ATN1-FL-26Q mice. Kruskal-Wallis multiple comparison analysis, Mean ± SEM, *$p<0.05$, **$p<0.01$ and ***$p<0.001$.

(I-J) Analysis of LaminB1 redistribution into the cytoplasm in fibroblasts from healthy controls and Vici Syndrom patients carrying mutations in the autophagy gene Epg5. Cells were treated for 48 hours with DSMO as a control or Rap, BafA1, BrefA, as well as combination of BafA1/Rap, and BafA1/BrefA. BrefA was only added for the last 24 hrs. BafA1 and BrefA treatment stimulate localisation of LaminB1 into the cytoplasm. Scale bar 50 µm (I). Automated quantification of area occupied by LaminB1 positive punctae

in the cytoplasm was performed using Opera Phenix system and Columbus softwarec (J). Fibroblasts with Epg5 mutation showed increased cytoplasmic LaminB1 levels in control condition and were more sensitive to all treatments with respects to control. Significance levels above the column, mean ± SEM, two-way-ANOVA ***$p<0.001$, **$p<0.01$, v1 – genotype, v2 - treatment. Treatment with BafA1/BrefA combination resulted in synergistic effect showing a significant increase compared to BafA1 and BrefA. In contrast BafA/Rap did not increase cytoplasmic LaminB1 levels in comparison to the BafA treatment alone: significance levels shown above the horizontal bars were determined by one-way-ANOVA ***$p<0.001$.

(K – L) Representative images of SK-N-BE(2) human neuroblastoma cells expressing EGFP and mCherry-LaminB1 and treated with BafA1 or DMSO as a control for 48 hrs (K). Automated quantification of area occupied by mCherry positive punctae in the cytoplasm was performed using Opera Phenix system and Columbus software BafA1 induced significant cytoplasmic accumulation of ectopically expressed mCherry-LaminB1 in the cytoplasm of EGFP positive cells (L). Note also the misshapen nucleus in the BafA1 treated cell compared to the control. Student's t-test, mean ± SEM, ***$p<0.001$. Scale bar 5 µm.

(M) Medium supernatant from DRPLA fibroblasts fractionated by ultracentrifugation. Truncated LaminB1 is specifically found in the fraction collected after ultracentrifucgation at 100,000g, known to contain extracellular vesicles. No LaminB1 is found in the fraction collected upon 10,000g ultracentrifugation, known to contain apoptotic bodies. A ponceau ban is used as a control for loading. Its molecular weight is consistent with that of BSA, a major component of the medium supernatant.
